# ANN-based swarm intelligence for predicting expansive soil swell pressure and compression strength

**DOI:** 10.1038/s41598-024-65547-7

**Published:** 2024-06-25

**Authors:** Fazal E. Jalal, Mudassir Iqbal, Waseem Akhtar Khan, Arshad Jamal, Kennedy Onyelowe, Lekhraj

**Affiliations:** 1 State Key Laboratory of Intelligent Geotechnics and Tunnelling, College of Civil and Transportation Engineering, Shenzhen University, Shenzhen, 518060 Guangdong China; 2 Key Laboratory of Coastal Urban Resilient Infrastructures (Shenzhen University), Ministry of Education, Shenzhen, China; 3 Department of Civil Engineering, University of Engineering and Technology Peshawar, Peshawar, Pakistan; 4 Department of Civil Engineering, University of Louisiana at Lafayette, Lafayette, LA 70503 USA; 5 Department of Civil Engineering, College of Engineering, Qassim University, Buraydah, 51452 Saudi Arabia; 6 Department of Civil Engineering, Kampala International University, Kampala, Uganda; 7 Department of Computer Engineering and Applications, GLA University, Mathura, 281406 India

**Keywords:** Expansive soils, ANN, Metaheuristics, PSO, SMA, GWO, MPA, Civil engineering, Software

## Abstract

This research suggests a robust integration of artificial neural networks (ANN) for predicting swell pressure and the unconfined compression strength of expansive soils (*P*_s_*UCS*-ES). Four novel ANN-based models, namely ANN-PSO (i.e., particle swarm optimization), ANN-GWO (i.e., grey wolf optimization), ANN-SMA (i.e., slime mould algorithm) alongside ANN-MPA (i.e., marine predators’ algorithm) were deployed to assess the *P*_s_*UCS*-ES. The models were trained using the nine most influential parameters affecting *P*_s_*UCS*-ES, collected from a broader range of 145 published papers. The observed results were compared with the predictions made by the ANN-based metaheuristics models. The efficacy of all these formulated models was evaluated by utilizing mean absolute error (MAE), Nash–Sutcliffe (NS) efficiency, performance index *ρ*, regression coefficient (*R*^2^), root mean square error (RMSE), ratio of RMSE to standard deviation of actual observations (RSR), variance account for (VAF), Willmott’s index of agreement (WI), and weighted mean absolute percentage error (WMAPE). All the developed models for *P*_s_-ES had an *R* significantly > 0.8 for the overall dataset. However, ANN-MPA excelled in yielding high *R* values for training dataset (*TrD*), testing dataset (*TsD*), and validation dataset (*VdD*). This model also exhibited the lowest MAE of 5.63%, 5.68%, and 5.48% for *TrD*, *TsD*, and *VdD*, respectively. The results of the *UCS* model’s performance revealed that *R* exceeded 0.9 in the *TrD*. However, *R* decreased for *TsD* and *VdD*. Also, the ANN-MPA model yielded higher *R* values (0.89, 0.93, and 0.94) and comparatively low MAE values (5.11%, 5.67, and 3.61%) in the case of PSO, GWO, and SMA, respectively. The *UCS* models witnessed an overfitting problem because the aforementioned *R* values of the metaheuristics were 0.62, 0.56, and 0.58 (*TsD*), respectively. On the contrary, no significant observation was recorded in the *VdD* of *UCS* models. All the ANN-base models were also tested using the a-20 index. For all the formulated models, maximum points were recorded to lie within ± 20% error. The results of sensitivity as well as monotonicity analyses depicted trending results that corroborate the existing literature. Therefore, it can be inferred that the recently built swarm-based ANN models, particularly ANN-MPA, can solve the complexities of tuning the hyperparameters of the ANN-predicted *P*_s_*UCS*-ES that can be replicated in practical scenarios of geoenvironmental engineering.

## Introduction

Expansive behaviour of swelling clay is a complicated process as prominent clay minerals, for instance, kaolinite, illite, montmorillonite etc. are present, which leads to higher swell-shrink as the moisture fluctuates. The physicochemical properties of the expansive soils (ES) are immensely perplexed. Their volume change behaviour is attributed to a typical S-shaped swelling characteristics curve in the form of a three-phase swelling which can be further compartmentalized as preliminary, primary and secondary swelling stages^1–8^.

Firstly, the larger stresses in the form of swelling pressures (*P*_s_), ASTM D4546, are generated when the volume change is blocked. The swelling pressure of ES (*P*_s_-ES) is a fundamental parameter in estimating the behaviour of soft clays as well as an imperative characteristic of designing geotechnical structures^7,9,10^. According to Meshram et al.^11^, it offers comparatively better correlations using mineralogical, geotechnical and microfabric characteristics. Several direct and indirect techniques are available to predict the *P*_s_-ES such that the latter methods are based on experimental results and engineering judgement. Furthermore, Du et al.^12^ and Yin et al.^13^ suggested that to characterize the *P*_s_ under various conditions, numerous predictive models have been developed, such as Gouy–Chapman diffused double layer models, heat-driven/energy-related models, and data-driven/hybrid models are the three types of existent models^14^. Secondly, the unconfined compressive strength of ES (*UCS*-ES) is a desideratum for various parameters used in road design, primarily for highway construction^15–17^. Also, the brittle behaviour of the ES yields low tensile strength thus leading to lesser *UCS*, and ASTM D2166, which could be improved by soil stabilization^18^. For instance, the *UCS* of lime-treated expansive soil increases at higher CaO content for various conditions, and additionally, the other engineering properties are also enhanced^15,19,20^. The highest *UCS* of CaO-stabilized ES was recorded for the samples compacted at their optimum moisture content (*OMC*)^15^. While evaluating the *UCS* for various drying-wetting cycles, Wu et al.^21^ reported that the *UCS*-ES decreased by around 50% after the first drying-wetting cycle (i.e., *UCS* is inversely related to the drying-wetting cycles), whereas it perpetually increased at extended curing periods.

A rich amount of literature exists on the influence of the ES characteristics, (such as distribution of the grain sizes, consistency limits, compaction characteristics, and swelling, among others) on their mechanical properties. For instance, the plasticity index (*PI*) increases at higher montmorillonite content which ultimately increases the *P*_s_*UCS*-ES. This cohesive nature can be associated with the low specific surface area (*SSA*) with higher cation exchange capacity (*CEC*) value of the smectites in the ES^22^. Similarly, maximum dry density (*MDD*) is another major indicator of the compressibility of the ES, and its high value depicts larger *UCS* and lesser P_s_, whereas the *OMC* behaves vice-versa^23^. Additionally, the natural water content (*w*_n_) also substantially impacts the swell-strength characteristics of various ES. At high values of the *w*_n_, more water enters the clay minerals which increases the swelling thereby leading to higher *P*_s_ and lesser values of the *UCS*-ES^24,25^.

Various machine learning (ML) algorithms approaches have been widely considered in the recent past that are capable of accurately predicting many real-world problems^26–29^. The recently developed AI techniques include artificial neural networks (ANNs)^30^, genetic-based programming^31^, eXtreme gradient boosting (XGBoost)^32,33^, multivariate adaptive regression splines (MARS)^34^, alternate decision trees (ADTs), logistic regression (LR), M5 model trees, genetic algorithm (GA) among others^35,36^. Giustolisi et al.^37^ classified the mathematical models, i.e., white, black, and grey box models (WBM, BBM, and GBM, respectively), such that the WBMs exhibit parameters based on physical laws which form accurate physical associations, but their hidden mechanism has not been fully understood. The BBMs incorporate regressive data-driven systems wherein the active associations are not known and require to be predicted. While the GBMs are methodical systems wherein a mathematical framework efficaciously determines the overall behaviour. In this regard, the ANN is classified as ‘BBM’ due to lesser transparency and their inability to form closed-form prediction equations^38,39^. The ML models are deployed to compute the *P*_s_*UCS*-ES, which are imperative for designing foundations as well as constructing pavements resting on swelling soils. In addition, these laboratory tests are time-consuming, whereas the problematic soils are found in over 40 countries across the globe^31^. The main advantage of the ANN approach to calculate *P*_s_*UCS*-ES is the capability to model complex, non-linear relationships between input variables and ES characteristics which lead to robust predictions compared to conventional methods The PSO is advantageous because of its rapid convergence ability, requiring only fewer parameters to adjust thus proving to be efficacious in dealing optimization problems^40^. GWO is advantageous owing to its balanced exploration and exploitation techniques that lead to enhanced convergence speed. Moreover, this algorithm is simple to implement and understand which renders it accessible to researchers and practitioners. SMA exhibits various merits because it is easy to implement, adaptable, and bio-inspired, and it explores the search space efficiently by simulating the growth and foraging behaviour of slime moulds. Finally, inspired by the hunting behaviour of marine predators the MPA is advantageous because of its diversity in maintenance, adaptive strategy, and efficient convergence which makes it suitable for various real-world applications^40–44^.

ANNs are computer programs which are used to estimate and categorize issues related to the information handling of the data^45,46^. They are inspired by the biological structure of our brain as well as the nervous system which directly captures the association between inputs and outputs, however, there is no empirical formulation yielded^47,48^. The formulated ANN model depicted that soil biochar composite having 5% biochar replacement yielded excellent results in lessening soil erosion. The ANN-based model forecasted the soil water characteristics curves reasonably well^49^. On the contrary, it was found by Das et al.^50^ that the SVM model outclassed the developed ANN models. In yet another study on *P*_s_-ES and *UCS*-ES (known as *P*_s_*UCS*-ES), the results of ANN modelling yielded the most satisfactory values in terms of *R-value* in the case of training as well as testing datasets (*TrD* and *TsD*, respectively). The comparison results showed that both the GEP and ANN are efficient and robust methods to determine the *P*_s_*UCS*-ES^31,51^. Therefore this study incorporates five advanced optimization methods, such as PSO^52^, GWO^53^, SMA^54^, MPA^43^ alongside the ANN modelling to enhance the predictive capability. Ikizler et al.^55^ formulated an ANN model to estimate the horizontal and vertical *P*_s_-ES. The ANN formulation decreases the number of laboratory tests thereby attaining cost-effectiveness and robustness. Kumar et al.^32,56^ used hybrid ANNs and deep learning-based simulation models (on 81 case histories of static pile load tests conducted in various regions of Vietnam) facilitating the safe and economical designs of eco-friendly piles. In a variety of geotechnical engineering systems, a lesser number of easily calculated input factors were used to model the unsaturated ES for the sake of predicting their mechanical behaviour^57^. In another finding, the modelling results of ANN estimated the mechanical properties of pond ash stabilized ES impressively (with a coefficient of correlation R ≈ 0.96)^58^. Recently, new empirical prediction models were developed by Jalal et al.^31^ for the determination of *P*_s_*UCS*-ES by deploying neural networks, i.e., ANN, adaptive neuro-fuzzy inference system (ANFIS), and genetic programming approach, i.e., GEP^59,60^. The results revealed that both the GEP as well as ANN are efficient methods to accurately compute *P*_s_*UCS*-ES. Furthermore, they suggested reliable and easy-to-use GEP equations for the prediction of *P*_s_*UCS*-ES are given in Eqs. (1) and (2), respectively.

1 $$ P_{s} = CF - \left( {\left( {\frac{7.25}{{G_{s} }}} \right)(OMC - SP + 0.91)} \right) + \left( {\left( {\frac{1}{3.71 + OMC} \times (MDD + 0.72)PI} \right) + OMC} \right) + \left( {\frac{1}{{\left( {\frac{1}{silt} - 52} \right)}} \times ( - 0.43\rho_{d\max }^{2} )} \right) $$

2 $$ UCS = \left( {\frac{sand(OMC - CF)}{{2w_{n} - OMC + G_{s} }}} \right) + \left( {sand + \rho_{d\max } + 0.19} \right)(\rho_{d\max } - 9.86) - \left( {\frac{silt}{{CF}}} \right) + (CF - (3 \times sand - 2G_{s} + SP)) $$

where *CF* is clay fraction, *G*_s_ is specific gravity, *MDD* is maximum dry density, *OMC* is optimum moisture content, *PI* is plasticity index, *SP* is the swell percent, and *w*_n_ is the natural moisture content.

The determination of *P*_s_-ES is time-consuming while the prediction of the *UCS*-ES is also cumbersome from the standpoint of time and cost. Previously, the *P*_s_*UCS*-ES have been determined by developing a variety of correlations using traditional statistical analyses (including GEP and ANN) wherein smaller R-values were recorded and the results were also not optimized^31,38^. However, Jumaa and Yousif^61^ found that the ANN outclassed the GEP model by yielding comparatively accurate performance. From the standpoint of these uncertainties, the existing research utilizes ANN in conjunction with PSO, GWO, SMA, and MPA to improve the past models to determine *P*_s_*UCS*-ES. Hyperparameter optimization is critical in ML model development which ensures optimal efficiency by fine-tuning parameters such as learning rates and regularization strengths. It is noteworthy to mention that the hyperparameter optimization review often highlights its role in improving model robustness while addressing issues such as computational complexity and overfitting. They also take into consideration the emerging methods, for example, Bayesian optimization and evolutionary algorithms, that encompass more efficacious exploration of hyperparameter spaces for better model generalization as well as robustness. Furthermore, the ANN-optimized models developed in the current study by using easily determinable geomechanical properties corroborated by past research^31,62–64^. Note that, *P*_s_ and *UCS* were the two output predictor variables. The motive of the research was to optimize the ANN models using recently developed algorithms, and to compare the performance of the developed models, such as (i) ANN-PSO, (ii) ANN-GWO, (iii) ANN-SMA, and (iv) ANN-MPA, for the estimation of *P*_s_*UCS*-ES by deploying simple geotechnical tests.

## Methodology

### ANN

These are simple yet dependable algorithmic models^40,41^. To accomplish particular tasks, the ANNs try to mimic how the human nervous system and brain work. Their use has significantly increased in recent years across several technical disciplines. In addition, they have also been applied in evaluating different characteristics of the ES^31^. Their structure as well as functioning, such that a distinctive ANN structure comprises many processing elements (i.e., nodes) which have been arranged in layers (like input, output, and hidden layer/s) has been previously described. Note that the best-hidden layer can be found through the trial and error method^65^. The input value of the preceding layer $({x}_{i})$ over every node is multiplied with the help of varying connection weight $\left({w}_{ji}\right).$ The addition process of weighted input signals took place on every node alongside the addition of a threshold value $\left({\phi }_{j}\right)$, too. After that, a non-linear transfer function $\left(f((.\right))$ is used over the joint input $\left({I}_{j}\right)$ for generating node output $\left({y}_{j}\right).$ It is important to state that the transfer functions commonly employed are linear and/or sigmoidal^66^.

3 $$ I_{ij} = \sum\limits_{i = 1}^{n} {w_{ji} + \phi_{j} } $$

4 $$ y_{j} = f(I_{j} ) $$

**Figure 1:**
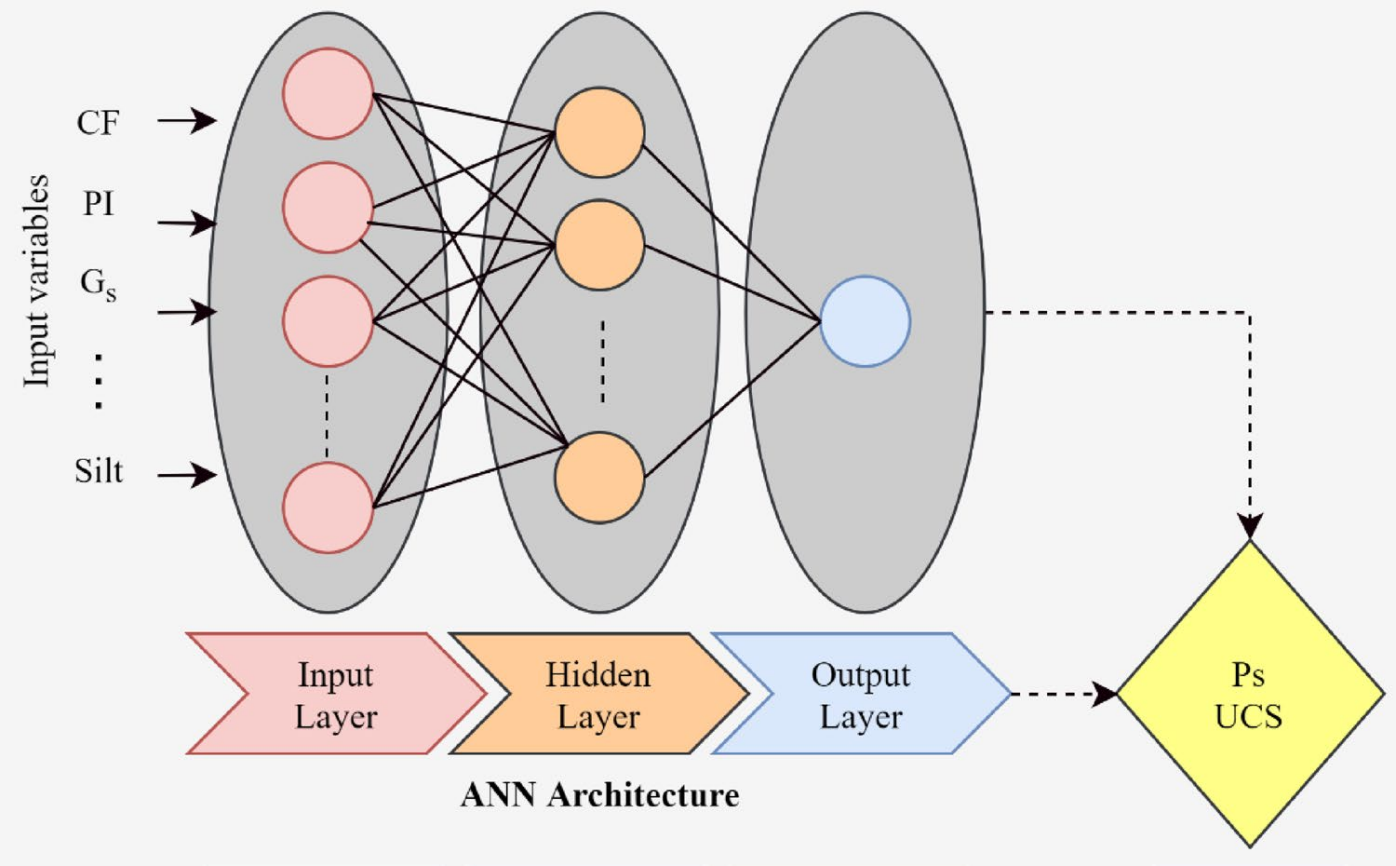
Architecture of developed ANN Model for estimation of *P*_*s*_*UCS*-ES in this study.

The output of a layer acts as input at nodes in subsequent layers whereas this procedure is iteratively repeated. The entire process is given in Fig. 1 whereas the pertaining formulae are expressed in Eqs. (3) and (4). The data is induced to the input layer after which the system weights must be attuned iteratively according to set guidelines for determining the best combination of weights via a ‘training’ procedure with the help of deploying Levenberg–Marquardt backpropagation approach. Finally, after sufficient training, the model is terminated when the changes in resulting error are minimal. Moreover, the entire data is divided into three distinct sets,

i.e., *TrD*, *TsD* and *VdD*. It is important to state that the ANNs use the training set to identify patterns in the data. Also, the network training evaluates the combination of weights ${w}_{ji}$ among different neurons for yielding a global minimum of the error function by(Eq. 5). Furthermore, the main objective of *TsD* aims at assessing the robustness of the trained network bt finally evaluating the *VdD*.

5 $$ y_{k}^{j} = f\left( {\sum\nolimits_{j = 1}^{nk - 1} {w_{ji}^{k} } + y_{j}^{n - 1} } \right) $$

More information about the ANN algorithm and accompanying mechanism can be found in available literature^22,41,47,64^.

### PSO

It is another evolutionary programming approach that is influenced by the flocking habits of birds as well as fish. This concept was given by Kennedy and Eberhart^67^ for the first time. The algorithm exhibits its roots in social psychology and artificial lifespan as well as engineering. Like other population-based metaheuristics, PSO has a “population of particles” that fly through the hyperspace solution via set velocities. Note that the velocities of each particle can be stochastically updated at each iteration based on the historical best location. A defined fitness function is used to derive both the particle as well as the best positions in the neighbourhood^68^.

**Figure 2:**
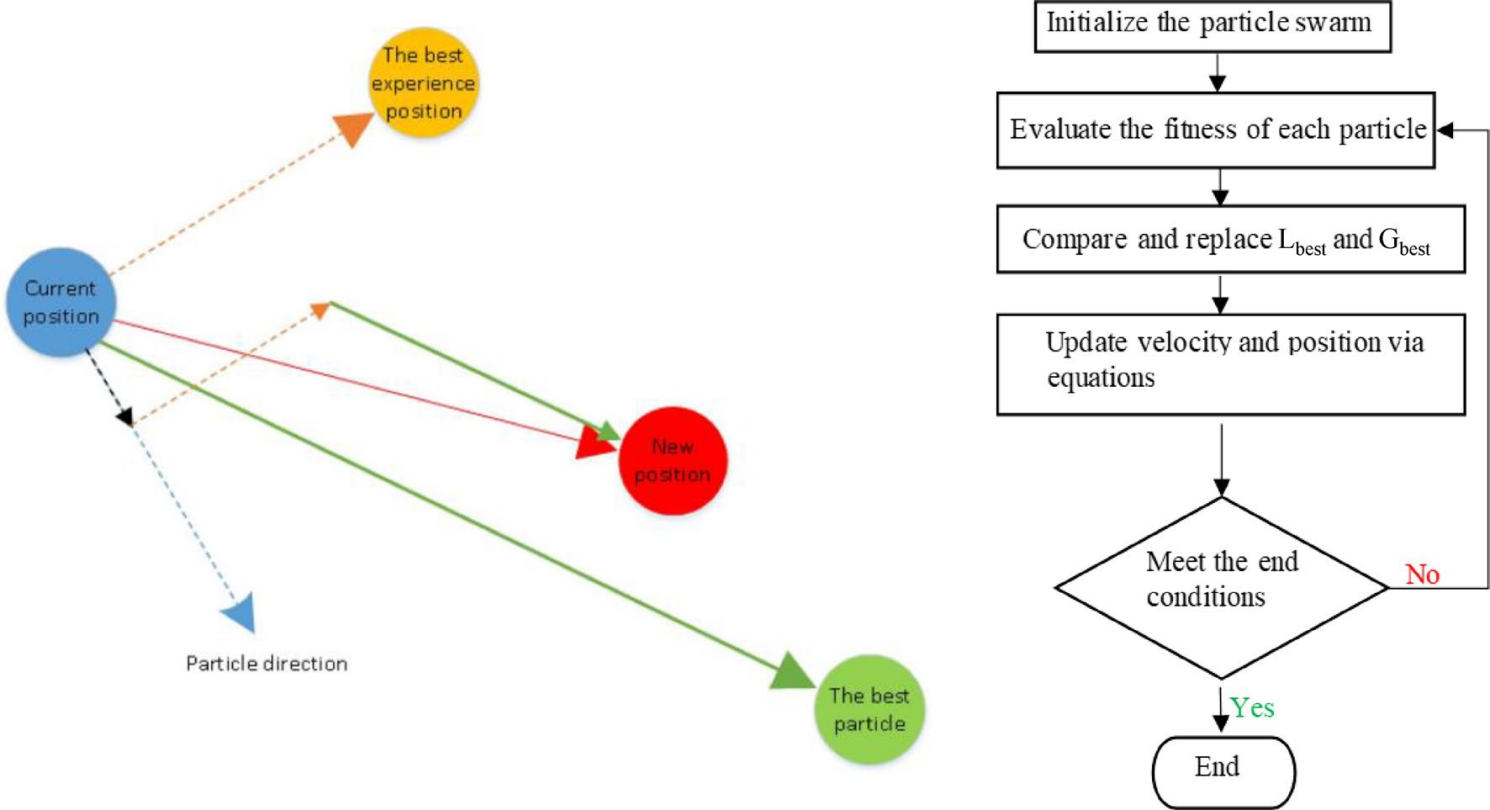
Schematic diagram of particle swarm optimization (PSO) algorithm (Modified after^69,70^).

In addition, each particle's motion naturally progresses towards the optimal or nearly optimal solution. At each iteration, the position of an individual particle can be adjusted accordingly. After that, the next generation swarm is produced based on revised particle locations seeing their individual best location (${L}_{best}$) and the entire swarm’s best position (${G}_{best}$) as depicted in Fig. 2. The positions of the particles and their velocities are computed by Eqs. (6) and (7):

6 $$ V{}_{i}^{t + 1} = wV_{t}^{t} + m_{1} n_{1} (L_{best,i}^{t} - Y_{i}^{t} ) + m_{2} n_{2} (G_{best,i}^{t} - Y_{i}^{t} ) $$

7 $$ Y{}_{i}^{t + 1} = Y_{i}^{t} + V{}_{i}^{t + 1} $$

where, ${V}_{i}^{t+1}$ and ${V}_{i}^{t}$ represent the particle $i$ velocities in the case of iterations *t* + *1* as well as *t*, respectively. Similarly, ${Y}_{i}^{t+1}$ and ${Y}_{i}^{t}$ denote the i^th^ positions in the case of iterations *t* + *1* and *t*, respectively. The parameters $w,$ indicates the cognitive social effects, ${m}_{1} and {m}_{2}$ denote the inertial parameters, and ${n}_{1} and {n}_{2}$ correspond to the matrix of arbitrary numbers with range [0,1]. The ${L}_{best}$ and the ${G}_{best}$ in the following generation is obtained using Eqs. (8) and (9):

8 $$ L_{best,i}^{t + 1} = \left\{ \begin{gathered} Y_{i}^{t + 1} ,h(Y_{i}^{t + 1} ) < h(L_{best,i}^{t} ) \hfill \\ L_{best,i}^{t} ,h(Y_{i}^{t + 1} ) \ge h(L_{best,i}^{t} ) \hfill \\ \end{gathered} \right. $$

9 $$ G_{best,i}^{t + 1} = \arg \min \{ h(L_{best,0}^{t + 1} ), \ldots ,h(L_{best,ns}^{t + 1} ),h $$

where ${n}_{s}$ represents the summation of particles in the swarm.

As the exploration for optimum solution progresses, the random and irregular movement of particles (swarm) in search space now closely replicates the swarm of mosquitoes. The main strength of adopting the PSO for complex real-life problems is that it is not largely influenced by non-linearity. Furthermore, PSO can exhibit better and faster convergence to optimum solutions in a variety of scenarios. It is computationally more exhaustive and robust than a variety of exact mathematical methods. However, like other metaheuristics, a key issue in applying PSO is to establish a reasonable trade-off between intensification (exploitation) as well as diversification (exploration). In recent years the algorithm has witnessed widespread applications such as power systems, traffic control^71^, geotechnical investigation^72^ and, rainfall-runoff modelling^73^.

### GWO

Mirjalili et al.^74^ floated the concept of this swarm intelligence optimization approach (metaheuristic algorithm) for the first time. The GWO draws inspiration from the cooperative hunting behaviour observed in grey wolves^75^. Metaheuristic algorithms are designed to generate high-quality solutions from a random population. The generation takes inspiration from natural system behaviours and continues until a specific termination condition is fulfilled^76^. GWO is based on three key steps i.e., surrounding prey, hunting, and *sand* attacking prey. To mathematically simulate wolf leadership order, assume the finest solution is alpha (α), the preceding one is beta (β), and finally it is the delta (δ). All other possible solutions can be assumed as omega (ω).

During the hunt the grey wolves encircle prey; the following equations (Eqs. 10 and 11) are given to numerically simulate grey wolf encircling behaviour.

10 $$ \vec{D} = \left| {C.\vec{X}_{prey(t)} - \vec{X}_{wolf} (t)} \right| $$

11 $$ \vec{X}_{wolf} (t + 1) = \vec{X}_{prey} (t) - \vec{A}.\vec{D} $$

$\overrightarrow{A}$ and $\overrightarrow{\text{C}}$ are the coefficient vectors, *t* represents existing iterations, and the prey position vector is ${\overrightarrow{\text{X}}}_{\text{prey}}$, and the grey wolf position vector is ${\overrightarrow{\text{X}}}_{\text{wolf}}$. The calculation of vectors $\overrightarrow{A}$ and $\overrightarrow{\text{C}}$ is according to Eqs. (12) and (13);

12 $$ \vec{A} = 2ar_{1} - a $$

13 $$ \vec{C} = 2r_{2} $$

where *r*_*1*_ and r_2_ are random vectors in the interval [0, 1], whereas *a* is linearly lowered from 2 to 0 throughout iterations.

Alpha (α) has usually guided the hunt, whereas, β as well as δ may take part in hunting occasionally. To mathematically model grey wolf hunting behaviour^77^, the first three optimal solutions are preserved, while ω are required to relocate by Eqs. (14) to (20).

14 $$ \vec{D}_{alpha} = \left| {C_{1} \cdot \vec{X}_{alpha} - \vec{X}} \right| $$

15 $$ \vec{D}_{beta} = \left| {C_{2} \cdot \vec{X}_{beta} - \vec{X}} \right| $$

16 $$ \vec{D}_{delta} = \left| {C_{3} \cdot \vec{X}_{delta} - \vec{X}} \right| $$

17 $$ \vec{X}_{1} = \vec{X}_{alpha} - A_{1} \cdot \vec{D}_{alpha} $$

18 $$ \vec{X}_{2} = \vec{X}_{beta} - A_{2} \cdot \vec{D}_{beta} $$

19 $$ \vec{X}_{3} = \vec{X}_{delta} - A_{3} \cdot \vec{D}_{delta} $$

20 $$ \vec{X}(t + 1) = \frac{{\vec{X}_{1} + \vec{X}_{2} + \vec{X}_{3} }}{3} $$

**Figure 3:**
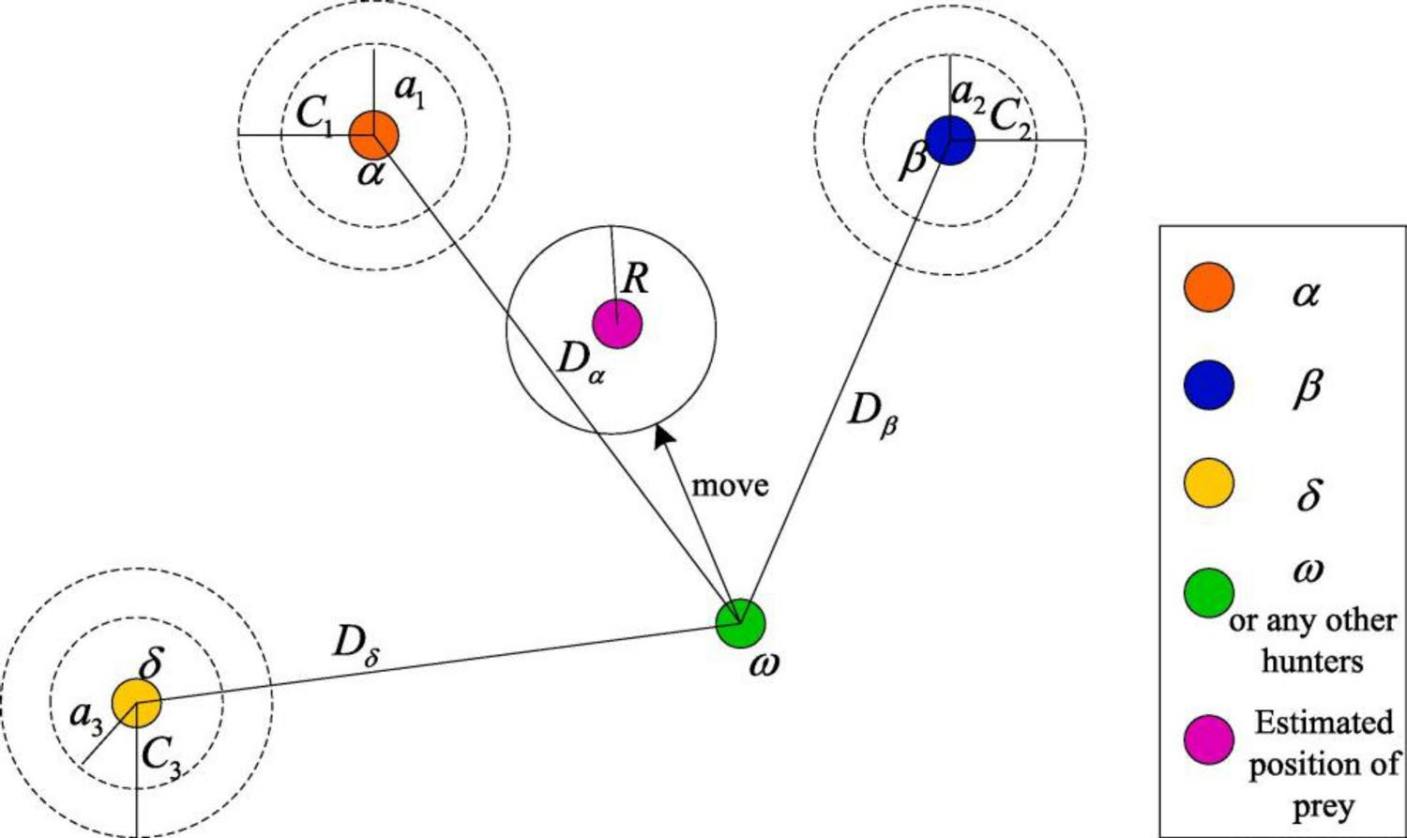
Position updating in Gray wolf optimization GWO, Adopted from^42^.

The new solution appears to be positioned at random within α, β, and δ. It is to say that, the new solution position can be evaluated using these three best solutions. The position updating in GWO is presented in Fig. 3. GWO is advantageous to optimize problems because of its viable properties in contrast to other metaheuristics^78^. This metaheuristic algorithm is also known for its simplicity, scalability, and special capability to keep the appropriate balance between diversification and intensification. In recent years, GWO has been employed for numerous engineering implications^79–82^.

### SMA

Li et al.^54^ introduced a modified stochastic optimization method, i.e., SMA, that entirely relies on the oscillating behaviour of slime mould (SM). The SMA independently follows the oscillation method, replicating the Physarum polycephalum activation and morphological changes of SM. This is done during exploration, searching and foraging all without finishing the lifespan. The SMA method incorporates highly customized and adaptive weights for modelling and generating true and false responses to the reaction of the SM. Thus, it creates the optimum path to link food using improved space exploration skills and great exploitation tendencies^44,54,83^.

**Figure 4:**
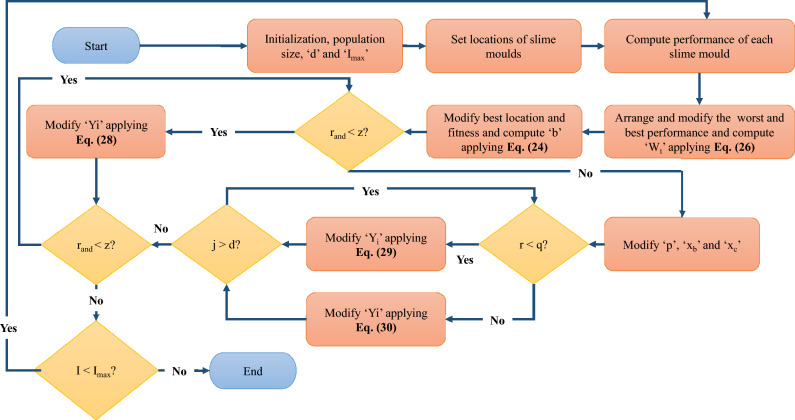
A pseudo-code-driven comprehensive flowchart of the slime mould algorithm (SMA).

The SMA optimization process operates in three distinct phases; (a) searching and approaching food using smell, (b) try wrapping the food as per the quality and composition of food, and (c) swinging and oscillating to seek a superior location^54,84^. The comprehensive mathematical explanation of every phase is examined in this section and is given in Fig. 4.

#### 1st Phase (Searching and approaching food)

In the first phase, the SM seek and approach food owing to its odour in the atmosphere as mathematically expressed by Eqs. (21) to (22).

When, $$r<q$$, then;

21 $$ Y_{i} = Y_{b} (t) + x_{b} [W_{t} .Y_{A} (t) - Y_{B} (t)] $$

When, $$r\ge q$$, then;

22 $$ Y_{i} = (x_{c} .Y_{i} ) $$

where, ${Y}_{i}$ refers to the location and orientation of the SM in the current cycle ($t$). ${Y}_{A}$ and ${Y}_{B}$ are two arbitrarily selected SM entities with weight (${W}_{t}$). ${Y}_{b}$ depicts the position of an entity with maximum saturation and concentration of odour. ${x}_{c}$ is the factor which lowers down linearly from 1 to 0. The other additional parameters such as $q$, ${x}_{b}$, and $b$ are specified in Eqs. (23) to (25).

23 $$ x_{b} = [ - b,b] $$

24 $$ b = \arctan h\left\{ { - \left( {\frac{t}{{t_{\max } }}} \right) + 1} \right\} $$

25 $$ q = \tanh \left| {S_{i} - F} \right|;i = 1,2, \ldots ,m $$

where, ${S}_{i}$ as well as $F$ indicate fitness of ${Y}_{i}$ and best performance among the total iterations completed, respectively. The ${W}_{t}$ can be explicitly stated in Eq. (26).

26 $$ W_{t} (smellindex(i)) = \left\{ {\begin{array}{*{20}c} {1 + r \cdot \log \left( {\frac{{BF - S_{i} }}{BF - WF} + 1} \right);Conditions} \\ {1 - r \cdot \log \left( {\frac{{BF - S_{i} }}{BF - WF} + 1} \right);Others} \\ \end{array} } \right. $$

27 $$ Smell - index = Sort(S) $$

where, $r$ shows the randomized variable between 0 and 1. $WF$ and $BF$ indicate the worst and optimum fitness within the latest iteration or cycle. The $smell index$ shows the arranged collection of best fittest scores, given as Eq. (27).

#### 2nd Phase (Wrapping food as per quality)

In the second phase, the vascular tissues of SM are squeezed. The ${W}_{t}$ of the space is regulated. The exploration and research of additional locations are conducted in this phase. When the bio-oscillator produces stronger and greater waves the cytoplasm starts travelling faster and the thicker and bigger vein receives the heavily saturated, concentrated and healthy food. With the rise of highly concentrated food, the ${W}_{t}$ of the search space rises and it is reduced owing to the low concentration. The algebraic interpretation of this phase is provided in the form of Eqs. (28) to (30).

28 $$ Y^{*} = r_{and} \cdot (V_{\max } - V_{\min } ) + V_{\min } ;r{}_{and} < z $$

29 $$ Y^{*} = Y_{b} + x_{b} \cdot \{ W_{t} \cdot (Y_{A} - Y_{B} )\} ;r < q $$

30 $$ Y^{*} = x_{b} \cdot Y;r \ge q $$

where, ${V}_{min}$ and ${V}_{max}$ show the searching region from minimum to maximum value, respectively.

#### 3rd Phase (waving and oscillation)

The SM depends completely on the propagation and amplification of waves produced during biological activity, such as changing the cytoplasmic flow in the veins. The ${x}_{b}$ varies in the range [− b, b]. It gradually approaches zero with the progression of the algorithm, as the number of iterations increases. While ${x}_{c}$ oscillates between [− 1,1] and it also eventually approaches zero.

#### Total net level of complexity of SMA:

The total net level of complexity of SMA comprises the complexity of the initialization process, performance assessment, strength or weight transformation and positioning^54^. Mathematically it can be provided in Eq. (31).

31 $$ SMA_{OverallNetComplexity} = C[d + t_{\max } .m.\{ 1 + \log (m) + d\} ] $$

where, $m$ and $d$ denote the maximum cells in the SM and the dimensionality of features, respectively.

The absence of an acceleration and mutation strategy may limit the wide-scale adoption of the SMA^85^. Furthermore, it also lacks in offering feature extraction when performed in the binary versions of algorithms.

### MPA

**Figure 5:**
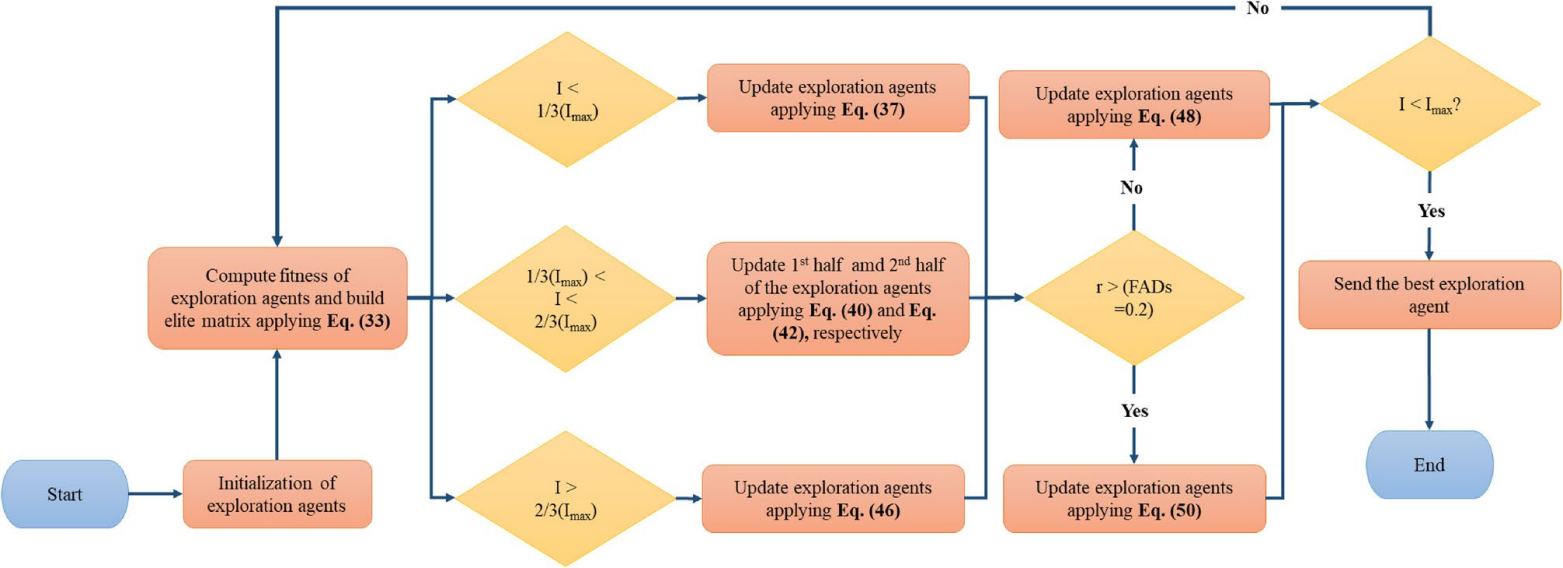
A pseudo-code-driven comprehensive flowchart of the marine predator algorithm (MPA).

Faramarzi et al.^43^ presented a novel marine predator algorithm (MPA), which works on the effective swarm-inspired metaheuristic. Unlike other evolution algorithms, swarm-inspired algorithms adapt and generate new approaches which are differentiated mainly by their ability to search across many networks for the best response^86^. As shown in Fig. 5, MPA pertains to general foraging tactics of aquatic and marine creatures, like Brownian motion and Levy flight of prey and predator (inspired organisms). It is followed by the optimal encounter rate strategy of biological predator–prey interactions^43^. The predator forages and eats, whilst the prey gets eaten. The ease and simplicity of the velocity-based MPA approach, along with its excellent performance make it a viable substitute for traditional optimization algorithms^43^.

Similar to the vast number of population-based metaheuristic algorithms, MPA is initialized with uniform allocation of the objective function and initial response in a search space, as expressed in Eq. (32).

32 $$ Y_{i,j} = V_{\min ,j} + \{ R \times (V_{\max ,j} - V_{\min ,j} )\} \;\;\;i = 1,{ }2,{ } \ldots ,{ }m{ }\;and\;j = 1,{ }2,{ } \ldots ,{ }d $$

$R$ is the evenly distributed vector on a random basis with a value ranging from 0 to 1 with ${V}_{min,j}$ and ${V}_{max,j}$ representing the minimum and maximum limits of the variable value to be assessed, respectively. In a search space, $d$ and $m$ indicate the highest dimension and total agents, respectively. ${Y}_{i,j}$ indicates the randomized matrix of the solution set *PI*cked randomly having $m\times d$ dimensional space.

As per the existence of the fittest concept, the best predators who are better at exploring, foraging and searching for prey are permitted to assemble an elite matrix to record cost-function data, as shown in Eq. (33).

33 $$ E_{lite} = \left[ {\begin{array}{*{20}c} {Y_{11}^{1} } & {Y_{12}^{1} ....} & {Y_{1\dim }^{1} } \\ \vdots & \ddots & \vdots \\ {Y_{n1}^{1} } & {Y_{n2}^{1} ....} & {Y_{n\dim }^{1} } \\ \end{array} } \right] $$

Both prey and predators are working as search agents, simultaneously. When the predators explore their prey, the prey simultaneously looks for its feed. Thus, the ${E}_{lite}$ is revised in the end stage of each loop if a leading predator is substituted with a healthier one.

Prey is a separate distinct matrix, equal in dimension to the Elite, that predators have access to change their positions. In short, the initiation of the algorithm produces the first prey, with the finest (predator) evolving into the Elite. Thus, another Eq. (34) is used to describe the prey matrix.

34 $$ P_{ry} = Y_{ij} = \left[ {\begin{array}{*{20}c} {Y_{11} } & {Y_{12} ....} & {Y_{1\dim } } \\ \vdots & \ddots & \vdots \\ {Y_{n1} } & {Y_{n1} ....} & {Y_{n\dim } } \\ \end{array} } \right] $$

The optimization of MPA includes three phases for revising, modifying and updating the original response with the search space, which are closely linked to the two foregoing matrices. All three phases are evaluated by the predator–prey velocity ratio. The first, second and third phase refers to a high, unit as well as low-velocity ratio, respectively. The comprehensive mathematical explanation of each stage is given below:

#### 1st Phase (exploration with high velocity)

After the completion of one-third of the total iterations, the predators explore and switch locations quicker than the prey with a high-velocity ratio. Following Eq. (35), the mathematical expression for the exploration can be written as Eqs. (36) and (37).

When;

35 $$ I < \frac{1}{3}(t_{\max } ) $$

Then;

36 $$ S_{i} = R_{b} \otimes (E_{lite(i)} - R_{b} \otimes Y_{i} );i = 1,2,...,m $$

37 $$ Y_{i} = Y_{i} + (C.R \otimes S{}_{i});C = constant = 0.5 $$

${R}_{b}$ is the randomized vector for representing the normally distributed Brownian motion. While $I$ and ${I}_{max}$ describes the present and maximum possible iteration, respectively.

#### 2nd Phase (evolution from exploration to exploitation with unit velocity)

In this phase, the space exploration is transitorily converted to exploitation and both the prey and predator alter location at similar velocity (with velocity-ratio ≈ 1.0). It occurs between one-third and two-thirds of the total iterations. However, if the prey is adopting Levy flight, then the most appropriate motion for the predator is Brownian motion, thus, the population is separated into two. Following Eq. (38), for a first and second half part, the step size and the position of prey can be mathematically expressed as Eqs. (39) to (40) and Eqs. (41) to (43), respectively.

When;

38 $$ \frac{1}{3}(I_{\max } ) < I < \frac{2}{3}(I_{\max } ) $$

For the first semi-population;

39 $$ S_{i} = R_{l} \otimes (E_{lite(i)} - R_{l} \otimes Y_{i} );i = 1,2, \ldots ,\frac{m}{2} $$

40 $$ Y_{i} = Y_{i} + (C.R \otimes S{}_{i});C = constant = 0.5 $$

For other semi-populations;

41 $$ S_{i} = R_{b} \otimes (R_{b} \otimes E_{lite(i)} - Y_{i} );i = 1,2, \ldots ,m $$

42 $$ Y_{i} = E_{lite(i)} + (C.F \otimes S{}_{i});C = constant = 0.5 $$

43 $$ CF = \left( {1 - \frac{I}{{I_{\max } }}} \right)^{{\frac{2I}{{I_{\max } }}}} $$

${R}_{l}$ is the randomized vector for representing the normally distributed Levy flight and $F$ is the adaptable variable governing the Brownian movement of predators.

#### 3rd Phase (exploitation with low velocity)

In the final stage of optimization, when the current iteration surpasses two-thirds of the total iterations, the perfect exploitation occurs. Unlike, the first phase, the predators switch their locations considerably more gradually than the prey with lower velocity-ratio. By Eq. (44); the completely altered position of predators adopting Levy flight is mathematically expressed as Eqs. (45) to (46).

if;

44 $$ I > \frac{1}{3}(t_{\max } ) $$

then;

45 $$ S_{i} = R_{l} \otimes (R_{l} \otimes E_{lite(i)} - Y_{i} );i = 1,2, \ldots ,m $$

46 $$ Y_{i} = E_{lite(i)} + (C.F \otimes S{}_{i});C = constant = 0.5 $$

#### Eddy's formation with possible impact

MPA incorporates the formation of eddy's and uses Fish Aggregating Devices (FADs) to find an alternative response to the influence of natural and environmental variables and, as a result, modify the predator behaviour^87,88^, as can be seen in Eqs. (47) to (50);

if;

47 $$ p \le (FADs = 2) $$

then;

48 $$ Y_{i} = Y_{i} + F \times [Y_{\min } + R \otimes (Y_{\max } - Y_{\min } )] \otimes X $$

if;

49 $$ p > (FADs = 0.2) $$

then;

50 $$ Y_{i} = Y_{i} + [FAD(1 - p) + p] \times (Y_{r1} - Y_{r2} ) $$

$p$ denotes the FADs probability and $X$ is the binary vector response. The subscripts $r1$ and $r2$ are representing the random locations of the prey matrix (${Y}_{i}$).

#### Marine memory

Marine predators are extremely proficient in recognizing the region of productive foraging. As a result, the marine working memory function is also assessed in the MPA optimization process^43^. The ultimate focus of this new function is to eliminate local points and recall the previous finest position to assist agents in increasing uniform convergence^89,90^.

**Figure 6:**
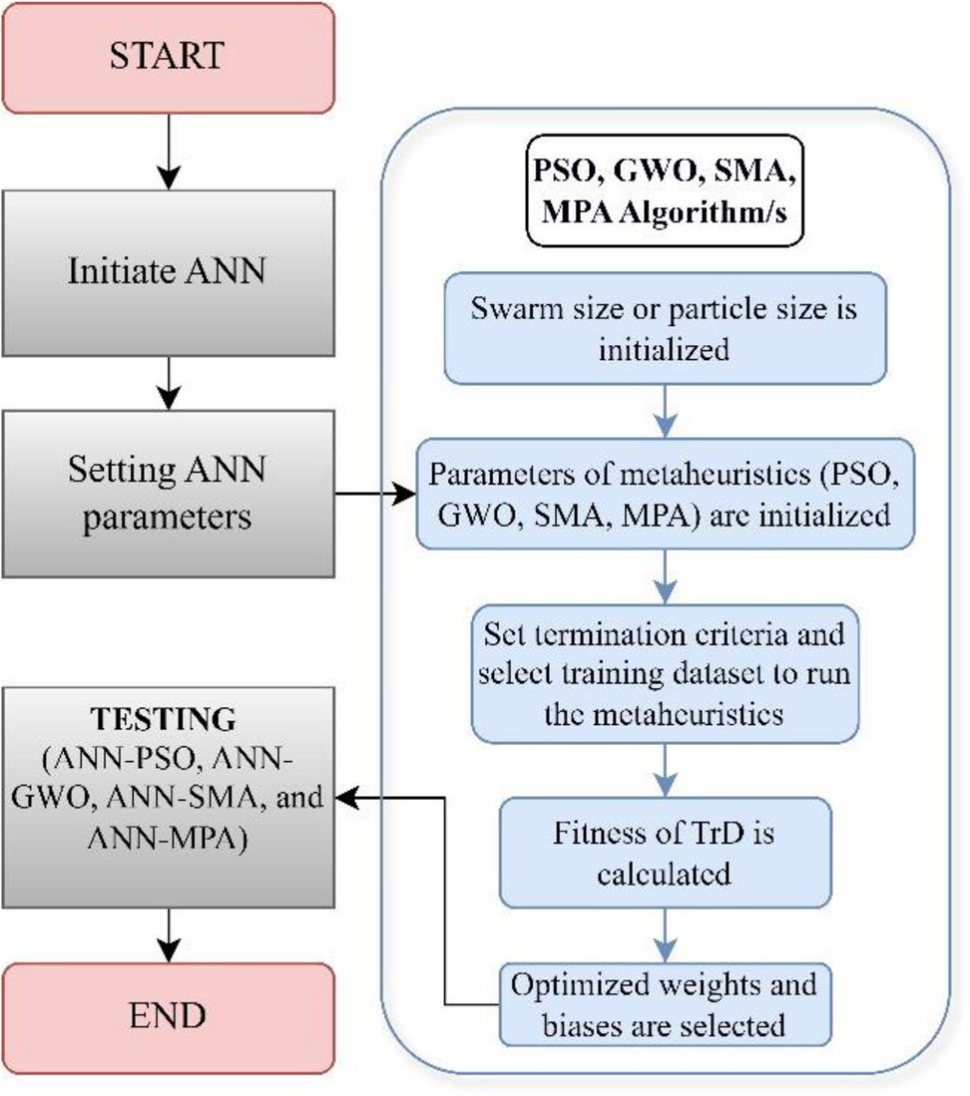
Flowchart for the mechanism of hybridization process of the metaheuristics used in the current study (With slight modifications after^92^).

As previously explained, MPA is referred to as solely a velocity-driven method, therefore introducing a binary multi-objective alternative can be a major enhancement^43,91^. Finally, Fig. 6 illustrates the construction steps of hybrid ANNs deployed in the current research to evaluate the *P*_s_*UCS*-ES. This figure outlines the process of using ANNs combined with swarm intelligence algorithms for optimizing the modelling of ES. It begins with the initialization of the swarm size and ANN parameters, followed by setting the metaheuristic parameters for algorithms such as PSO, GWO, SCA, and MPA approaches. Furthermore, this process includes testing the metaheuristics with ANN and selecting the best fit, which is then utilized to calculate the optimized weights and buses for the ANN model. Each model was ranked based on its performance in training and testing, with the highest scores given to the top performers and the lowest to the underperformers for each metric. The final score for each model was calculated by summing these individual rankings. Ultimately, the combined scores from both phases determined the model's overall ranking^32^. As a result, this leads to a sustainable construction approach by enhancing the understanding of the swell-strength nature of the problematic soils.

## Data processing and analysis

### Data preprocessing

To formulate the prognostic models, 168 and 145 observations of *P*_s_ and *UCS*, from 61 and 99 internationally published papers (Table 1), respectively, were considered. In addition, nine basic soil characteristics were collected from two separately developed databases. The original database was constructed after an extensive literature study by initially recording 250 datasets (for *P*_s_-ES) and 190 datasets (for *UCS*-ES). After that, easy-to-determine geotechnical parameters were recorded for developing models to predict the swell-strength properties of ES. After the collection of all data points, numerous ANN trials were run to evaluate the validity. The data points that diverged substantially (around 20% or more) from the general trend were ignored (i.e., 82 records for *P*_s_ and 45 records for *UCS*). Therefore, 168 observation points of *P*_s_-ES and 145 points of *UCS*-ES were finally deployed to formulate the hybrid models. The important factors affecting the *P*_s_*UCS*-ES were investigated based on a recent literature review. However, the swell percent, *MDD* and *OMC* for some cases were absent and correlations were used to determine the missing values. Similarly, an average value of G_s_ was considered for some of the datasets^31^. Additionally, the contribution of G_s_ (between 2.3 and 2.8) on the *P*_s_*UCS*-ES was negligible owing to its small range, however, it was considered by Akan and Keskin^93^ for predicting the *UCS*-ES. The information related to several other geotechnical factors was scarcely present in the existing literature for several datasets. As a result, it could significantly reduce the total number of observations. Also, it may affect the generalization capability of the predicted models. As a result, these parameters were omitted in the development of models in the current study.

**Table 1:** Researches ID and research references of the two expansive soil databases collected in this study.

ID	References	ID	References	ID	References
A. Swell pressure					
1	Taher et al., 2020	22	Zumrawi, 2015a	43	Basma et al., 1998
2	Al-Rawas et al., 2005	23	Ameta et al., 2007	44	Kumar et al., 2020
3	Soundara and Selvakumar, 2020	24	Dang et al., 2016, Hasan et al., 2016	45	Ozer et al., 2012
4	Al-Rawas, 1999	25	Shalabi et al., 2017	46	Yenes et al., 2012
5	Shi et al., 2002	26	Zumrawi and Mohammed, 2017	47	Baby et al., 2016
6	Phani Kumar and Sharma, 2004	27	Zumrawi et al., 2017b	48	Mirzababaei et al., 2017
7	Sabtan, 2005	28	Akgün et al., 2018	49	Kowalska and Ptaszek, 2019
8	Puppala et al., 2006	29	Lin and Cerato, 2014	50	Kaczyński and Grabowska-Olszewska, 1997
9	Seda et al., 2007	30	Dayioglu et al., 2017	51	Azzam, 2012
10	Yan and Wu, 2009	31	Zumrawi, 2015b	52	Carraro et al., 2010
11	Zheng et al., 2009	32	Eyo et al., 2019	53	Yazdandoust and Yasrobi, 2010
12	Abdalqadir et al., 2020	33	Syed et al., 2020	54	Trouzine et al., 2012
13	Lin and Cerato, 2011	34	Gupta and Sharma, 2016	55	Rashid, 2015
14	Sabat and Nanda, 2011	35	Parik and Patra, 2020	56	Mujtaba et al., 2018
15	Al-Mukhtar et al., 2012	36	Zumrawi et al., 2017a	57	Pedarla et al., 2019
16	Gandhi, 2013	37	Chittoori et al., 2019	58	Benyahia et al., 2020
17	Rashid et al., 2013	38	Gheris and Hamrouni, 2020	59	Mumtaz et al., 2020
18	Sabat, 2013	39	Phanikumar and Singla, 2016	60	Khennouf and Baheddi, 2020
19	Malekzadeh and Bilsel, 2014	40	He et al., 2018	61	She et al., 2020
20	Radhakrishnan et al., 2014	41	Rosenbalm and Zapata, 2017		
21	Reddy et al., 2015	42	Ramesh et al., 2012		
B. Unconfined compression strength					
1	Blayi et al., 2020	34	Zumrawi and Babikir, 2017	67	Onyelowe et al., 2016
2	Sharma and Sivapullaiah, 2016	35	Ismaiel and Abdellateef	68	Onyelowe, 2016
3	Kate, 2005	36	Ma et al., 2018	69	Signes et al., 2016
4	Modarres and Nosoudy, 2015	37	Sridevi et al., 2019	70	Gadouri et al., 2017
5	Puppala et al., 2001	38	Mazhar et al., 2018	71	Onyelowe, 2017
6	Bell, 1996	39	Ali and Mohamed, 2018	72	Shafiqu and Hasan, 2018
7	Okagbue and Onyeobi, 1999	40	Elkady, 2016	73	Tabet et al., 2018
8	Shi et al., 2002	41	Gonawala et al., 2019	74	Abd El-Aziz and Abo-Hashema, 2013
9	Phani Kumar and Sharma, 2004	42	Pooni et al., 2020	75	Akinwumi et al., 2019
10	Saride et al., 2010	43	Fattah et al., 2020	76	Almurshedi et al., 2019
11	Ene and Okagbue, 2009	44	Ikeagwuani et al., 2020	77	Emmanuel et al., 2019
12	Oriola and Moses, 2010	45	Jain and Jha, 2020	78	Irshayyid and Fattah, 2019
13	Seco et al., 2011	46	Ijaz et al., 2020	79	Carraro et al., 2010
14	Al-Mukhtar et al., 2012	47	Mazhar and GuhaRay, 2020	80	Pourakbar et al., 2016
15	Thyagaraj et al., 2012	48	Syed et al., 2020	81	Mujtaba et al., 2018
16	Rao and Thyagaraj, 2003	49	Sadeeq et al., 2015	82	Murmu et al., 2019
17	Venkara Muthyalu et al., 2012	50	Parik and Patra, 2020	83	Saputra and Putra, 2020
18	Kanawi and Kamel, 2013	51	Tran et al., 2017	84	Khalid et al., 2019
19	Khemissa and Mahamedi, 2014	52	Chittoori et al., 2019	85	Adeyanju et al., 2020
20	Dafalla et al., 2015	53	Miao et al., 2017	86	Tiwari et al., 2020
21	Modarres and Nosoudy, 2015	54	Gheris and Hamrouni, 2020	87	Baldovino et al., 2020
22	Ameta et al., 2007	55	He et al., 2018	88	Mir and Wani, 2018
23	Sivakumar Babu et al., 2008	56	Kushwaha et al., 2018	89	Eyo et al., 2020
24	Amadi and Osu, 2018	57	Öncü and Bilsel, 2018	90	Ali et al., 2020
25	Osinubi et al., 2009	58	Soğancı, 2015	91	Indiramma et al., 2020
26	Dahale et al., 2016	59	Hussain, 2020	92	Mumtaz et al., 2020
27	Dang et al., 2016	60	Al-Khashab and Thafer, 2008	93	Khodabandeh et al., 2020
28	Eissa et al., 2017	61	Solanki et al., 2009	94	Krishnan and Ravichandran, 2020
29	Shalabi et al., 2017	62	Patel, 2013	95	Lu et al., 2020
30	Zumrawi and Mohammed, 2017	63	Kolay and Rahman, 2016	96	Meeravali et al., 2020
31	Acharya et al., 2017	64	Khalid et al., 2015	97	Nikhil et al., 2020
32	Kumar et al., 2007	65	Alazigha et al., 2016	98	Niyomukiza and Setiadji, 2020
33	Harichane et al., 2018	66	Canakci et al., 2016	99	Phanikumar and Raju, 2020

### Descriptive statistics and statistical visualization

**Figure 7:**
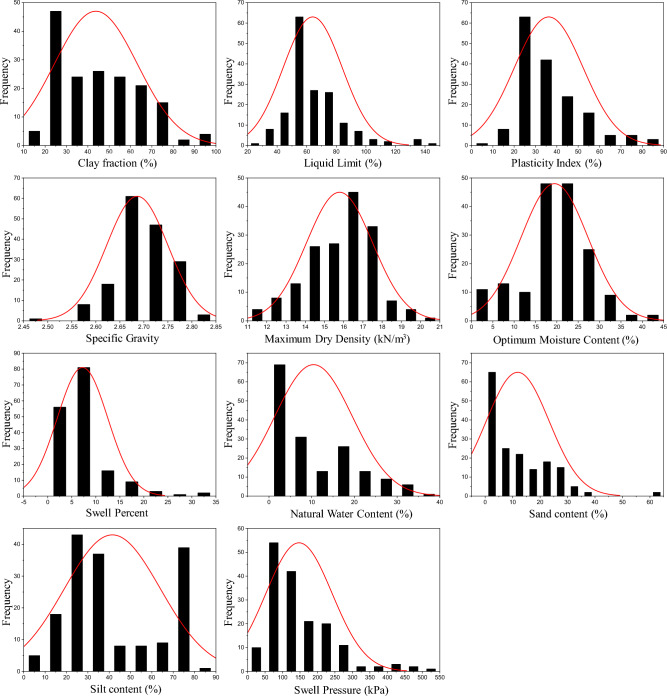
Distribution histogram of (**a**) clay fraction *CF*, (**b**) liquid limit *LL*, (**c**) plasticity index *PI*, (**d**) specific gravity *G*_s_, (**e**) maximum dry density *MDD*, (**f**) optimum moisture content *OMC*, (**g**) swell percent *SP*, (**h**) natural water content *w*_n_, (**i**) *sand*, (**j**) *silt*, and (**k**) swell pressure *P*_*s*_.

**Figure 8:**
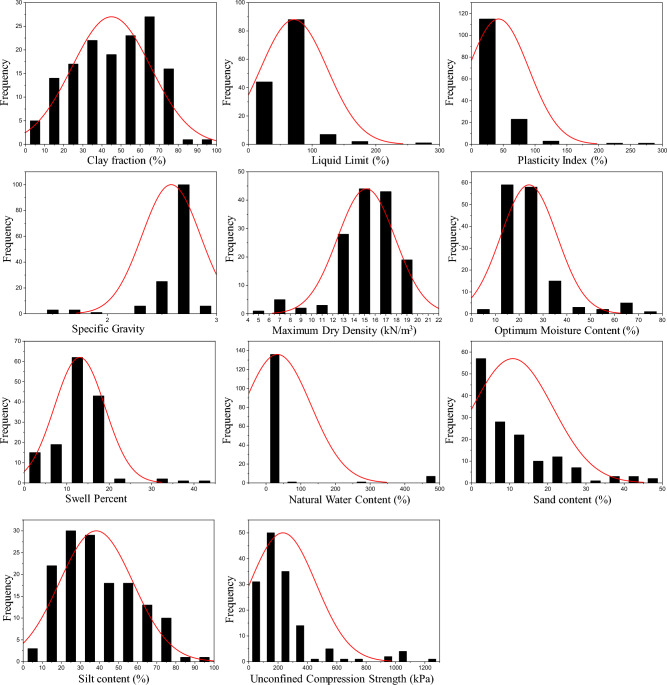
Distribution histogram of (**a**) clay fraction *CF*, (**b**) liquid limit *LL*, (**c**) plasticity index *PI*, (**d**) specific gravity *G*_s_, (**e**) maximum dry density *MDD*, (**f**) optimum moisture content *OMC*, (**g**) swell percent *SP*, (**h**) natural water content *w*_n_, (**i**) *sand*, (**j**) *silt*, and (**k**) unconfined compression strength *UCS*.

**Figure 9:**
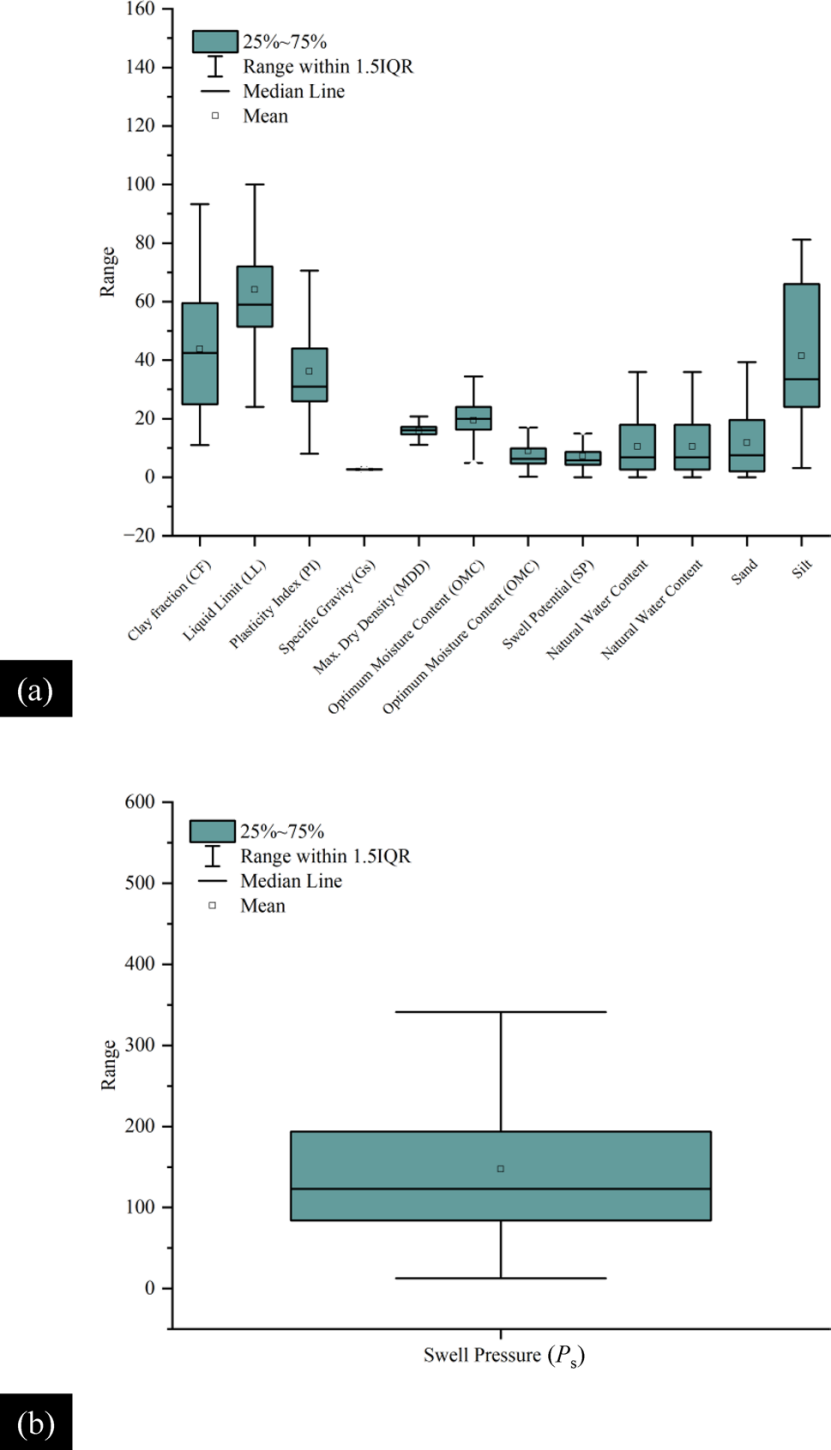
Box plots of input parameters and the output value (i.e., Swell Pressure *P*_s_).

**Figure 10:**
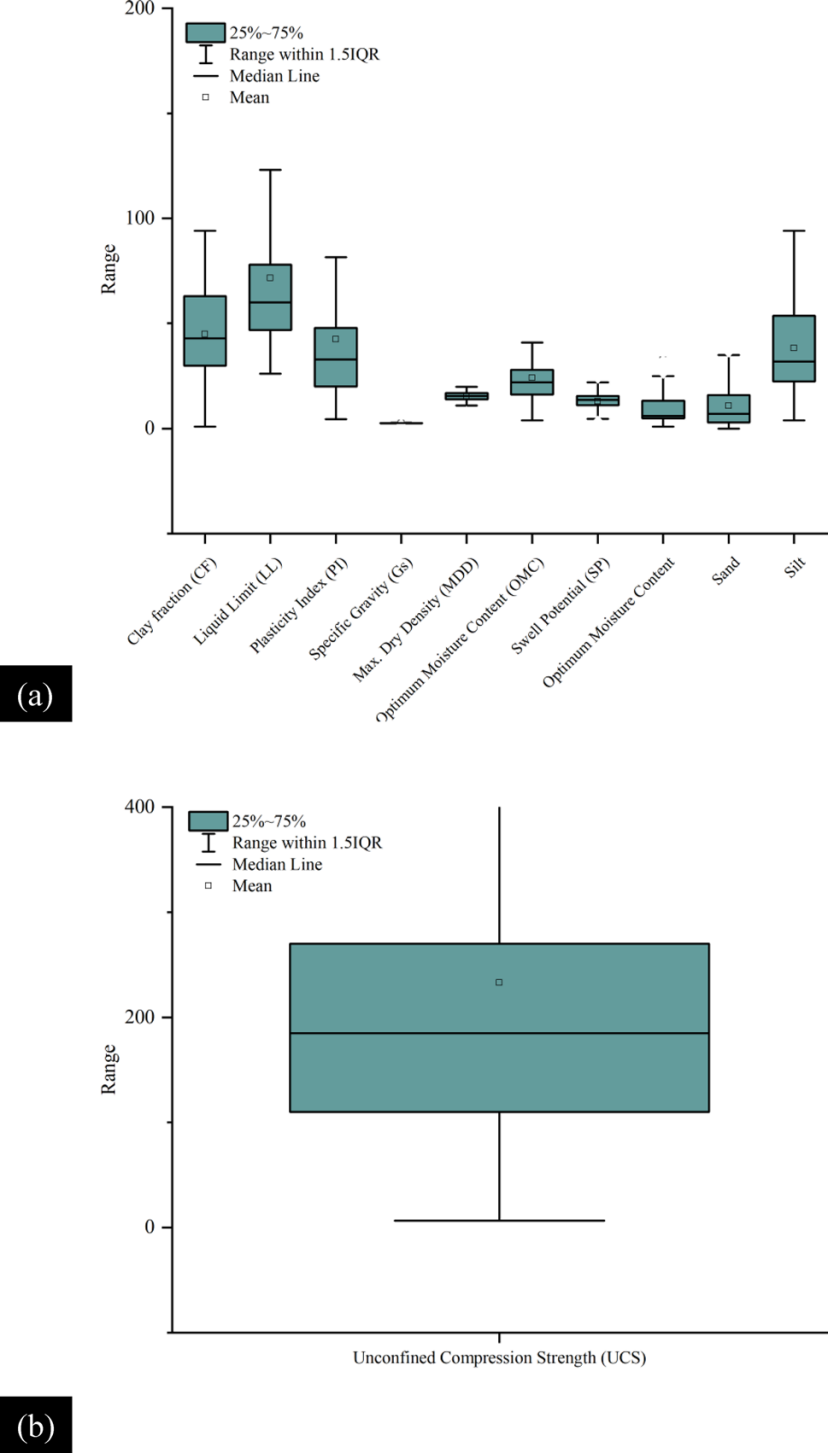
Box plots of input parameters and the output value (i.e., Unconfined compressive strength *UCS*).

Table 2 presents the descriptive statistics of the considered input as well as the two outputs such that these geotechnical indices are observed to affect the *P*_s_*UCS*-ES. It is shown in Table 2, that the *P*_s_*UCS*-ES range between 12.5 and 521 Kpa, and 6.4 and 1060 kPa, respectively. Additionally, *w*_n_ and *sand* content values for the *P*_s_ have not been included because their impact is lesser for the given range of data. Note that the *w*_n_ of the ES is different at different temperatures and drying times. However, the motive for selecting the *w*_n_ as an input parameter is due to its close association with the plastic limit and a variety of environmental factors. According to Patel^94^, the swelling capacity of ES primarily relies on its mineral composition, as well as the moisture content and density present in its natural environment. In general, clays with *PI* > 25, *LL* > 40, and *w*_n_ near the PL or less may witness higher expansion. Also, the ES are problematic owing to their mechanical behaviour which is largely hydrophilic^95^. Also, the Pearson correlation coefficient (r) calculated for the *w*_n_ was − 0.23293 for *UCS*-ES. It is known that the r-values illustrate a higher share of changes in the engineering characteristics of the ES. Moreover, the values given in Table 2 are suggested for the evaluation of *P*_s_*UCS*-ES using the aforementioned computational intelligence models in the current research study. The efficacy and robustness of the formulated models is significantly affected by the dispersal of various data points^47^. Moreover, to envisage the association among the ES input factors, graphical plots are given in Figs. 7 and 8 which depict the distribution histograms of various input factors as well as the two outputs (*P*_s_ and *UCS*), respectively The distribution of input data for *P*_s_-ES is shown as a box plot in Fig. 9a which shows the 25% to 75% data distribution alongside the visual interpretation of the mean and median of the given dataset. Similarly, the box plot for the *P*_s_-ES is manifested in Fig. 9b. Most of the data points considered in this study vary between 70 and 200. Secondly, the distribution of input data for *UCS*-ES is supplemented with a box plot shown in Fig. 10a which shows the 25% to 75% data distribution alongside the visual interpretation of the mean and median of the given dataset. Similarly, for the *UCS*-ES, the box plot is manifested as Fig. 10b. Most of the data points considered in this study vary between 100 and 300 MPa.

**Table 2:** Descriptive statistics of different input as well as output factors deployed in ANN-based formulated models (ANN-PSO, ANN-GWO, ANN-SMA)^31^.

	Swell pressure (*P*_*s*_)							Unconfined compression strength (*UCS*)							
	Min	Max	Mean	Median	SD	Kurtosis	Skewness	Min		Max	Mean	Median	SD	Kurtosis	Skewness
INPUT parameters															
Clay Fraction (%)	11	91	43.5	43	19.2	− 0.70	0.44	1		78	42.2	40	20.5	− 0.96	− 0.14
Plasticity index (%)	8	83	36.1	31.5	15.5	3.60	1.52	4.5		352	39.9	34	41.2	23.36	4.53
Specific gravity	2.3	2.8	2.7	2.7	6.6	8.61	− 1.66	1.4		2.8	2.6	2.7	0.3	11.85	− 3.41
Maximum dry density (kN/m^3^)	11.1	20.8	16.8	16	1.8	0.08	− 0.31	7.1		19.5	15	15.4	2.8	1.84	− 1.10
Optimum moisture content (%)	1.3	40	19.7	20	8.1	0.20	− 0.26	8		65	24.4	22.5	12.1	4.38	1.88
Swell percent (%)	0	32.7	7.4	5.9	5.8	6.01	2.09	2.2		32.7	12.9	13.5	4.7	5.29	1.18
Natural water content (%)	–	–	–	–	–	–	–	0.9		450	38.9	6.1	109.2	14.44	4.00
*Sand* (%)	–	–	–	–	–	–	–	0		49	11.4	7	11	1.95	1.49
*Silt* (%)	6	81.2	41.3	33	22.7	− 1.24	0.47	7		94	39.6	33	20.2	− 0.55	0.55
OUTPUT parameters															
*P*_*s*_ and *UCS*, respectively (kPa)	12.5	521	145.4	123.5	92.9	2.60	1.49	6.4		1060	226.1	188	219.6	6.77	2.51

The “–” represents missing data.

*Min* minimum, *Max*. maximum, *SD* standard deviation.

**Figure 11:**
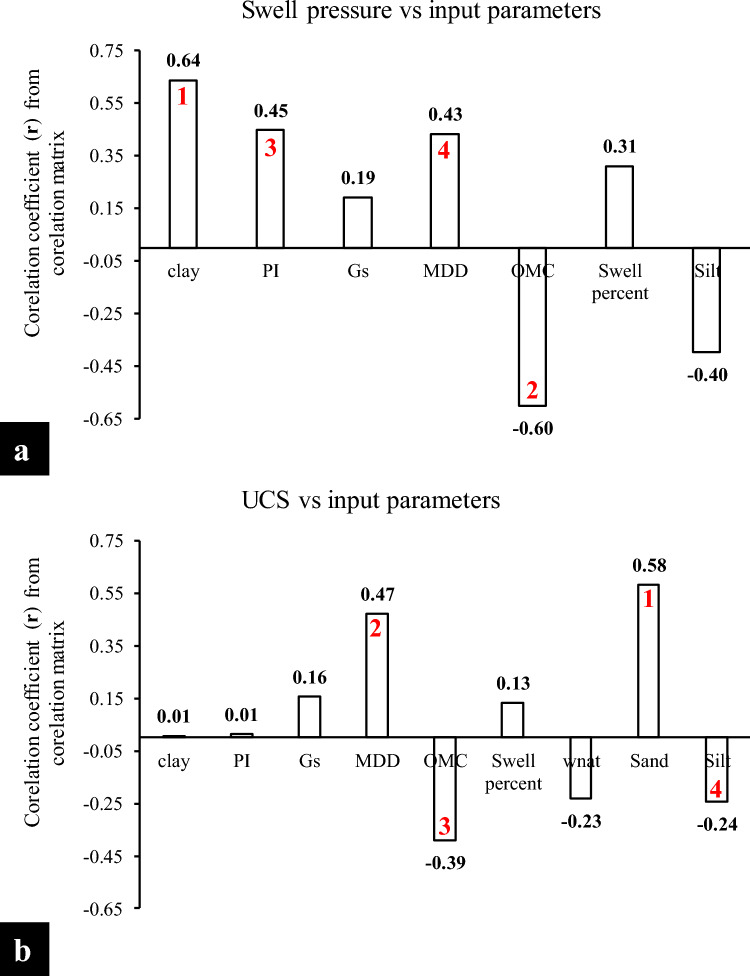
Correlation coefficient matrix results plotted for swell pressure as well as *UCS* of the expansive soils (*P*_*s*_*UCS*-ES).

The Spearman rank correlation coefficient for *P*_s_ *UCS*-ES has been plotted in Fig. 11a,b, respectively. One of the most widely employed measures of relationship is Pearson's correlation coefficient, which is generally given by r^31,96^. In the current research, nine parameters were selected to model *P*_s_ *UCS*-ES to avoid further complexity of the developed models. Note that, the *P*_s_-ES is largely governed by all parameters especially *CF* (r = 0.64), *OMC* (r = − 0.60) and *PI* (r = 0.45), while, *UCS*-ES is significantly influenced by *sand* content (r = 0.58), *MDD* (r = 0.47) and *OMC* (r = − 0.39), respectively. By and large, a high correlation prevails in the *P*_s_*UCS*-ES in the case of all input factors here.

### AI-based analysis

The collected databases (168 instances for *P*_*s*_, and 145 instances for *UCS*) were distinctly distributed as *TrD* and *TsD*. Note that the testing was performed to check the accuracy and robustness of the trained model using unseen data. Therefore, 70% of the dataset was selected randomly as the *TrD*, while the remaining 30% dataset was employed to test and validate the formulated models. Taherdangkoo et al.^97^ developed an efficient neural network model to determine the maximum *P*_s_ of clayey soils by partitioning the dataset into ratios of 70:30. Several other studies in the same field follow the same partitioning ratio^31,98,99^.

To evaluate the performance of the formulated models, commonly employed performance indices such as MAE, NSE efficiency, *P*_i_, *R*^2^, RMSE, RSR, VAF, WI, and WMAPE were determined^100–102^. The formulae of these indices can be expressed as Eqs. (51) to (59), respectively:

51 $$ MAE = \frac{{\sum\nolimits_{i = 1}^{n} {\left| {e_{i} - p_{i} } \right|} }}{n} $$

52 $$ NS = 1 - \frac{{\sum\nolimits_{i = 1}^{n} {(e_{i} - p_{i} )^{2} } }}{{\sum\nolimits_{i = 1}^{n} {(e_{i} - \overline{e}_{i} )^{2} } }} $$

53 $$ P_{i} = adj.R^{2} + 0.01VAF - RMSE $$

54 $$ R^{2} = \left( {\frac{{\sum\nolimits_{i = 1}^{n} {(e_{i} - \overline{e}_{i} )(p_{i} - \overline{p}_{i} )} }}{{\sum\nolimits_{i = 1}^{n} {(e_{i} - \overline{e}_{i} )^{2} \sum\nolimits_{i = 1}^{n} {(p_{i} - \overline{p}_{i} )^{2} } } }}} \right)^{2} $$

55 $$ RMSE = \sqrt {\frac{{\sum\nolimits_{i = 1}^{n} {(e_{i} - p_{i} )^{2} } }}{n}} $$

56 $$ RSR = \frac{RMSE}{{\frac{1}{n}\sum\nolimits_{i = 1}^{n} {(e_{i} - e_{mean} )}^{2} }} $$

57 $$ VAF(\% ) = (1 - \frac{{{\text{var}} (e_{i} - p_{i} )}}{{{\text{var}} (e_{i} )}}) \times 100 $$

58 $$ WI = 1 - \left[ {\frac{{\sum\nolimits_{i = 1}^{n} {(e_{i} - p_{i} )^{2} } }}{{\sum\nolimits_{i = 1}^{n} {\{ \left| {p_{i} - e_{mean} } \right| + \left| {e_{i} - e_{mean} } \right|\}^{2} } }}} \right] $$

59 $$ WMAPE = \frac{{\sum\nolimits_{i = 1}^{n} {\left| {\frac{{e_{i} - p_{i} }}{{e_{i} }}} \right| \times e_{i} } }}{{\sum\nolimits_{i = 1}^{n} {e_{i} } }} $$

where ${y}_{i}$ and ${\widehat{y}}_{i}$ refer to actual and predicted ith values, $n$ means data samples in a dataset, ${y}_{mean}$ refers to the average of the actual values whereas $p$ means the total input parameters.

## Results and discussion

This section presents the detailed results of the developed models to predict the *P*_s_*UCS*-ES. For both the target variables, similar nine attributes, namely, clay fraction *CF*, liquid limit *LL*, plasticity index *PI*, maximum dry density *MDD*, optimum moisture content *OMC*, swell percent *SP*, natural water content *w*_n_, *sand* and *silt* acted to be the input parameters, as mentioned earlier. As a result, 168 experimental results for *P*_s_-ES and 145 records of *UCS*-ES were employed. Initially, 70% of the data was utilized as the *TrD*, whereas the remaining data was separated into validation dataset (*VdD*) and training dataset (*TsD*). Subsequently, the performance of the formulated models was validated and tested with the help of the aforementioned performance indices. Moreover, the comparison of robustness as well as the general performance of the formulated models is also described. Finally, statistical testing and uncertainty analysis (UA) were performed to determine the overall performance of the ANN-based models.

### Configuration of ANN hybrid models

It is a desideratum to initially determine the optimum hyperparameters for the development of ANN-based models which is generally established using a trial-and-error procedure^103^. The optimum number of neurons achieved from trials for both *P*_s_-ES and *UCS*-ES models varied from 8 to 14, as listed in Table 3. The maximum number of iterations (*k*), as well as swarm size (*n*_s_), were kept constant during modelling at 500 and 50, respectively, to compare the developed models.

**Table 3:** Parametric configuration of the developed hybrid ANN models.

Parameters	*P*_*s*_-ES				*UCS*-ES			
	ANN-PSO	ANN-GWO	ANN-SMA	ANN-MPA	ANN-PSO	ANN-GWO	ANN-SMA	ANN-MPA
${n}_{h}$	10	8	12	12	11	10	13	14
${n}_{s}$	50	50	50	50	50	50	50	50
$k$	500	500	500	500	500	500	500	500
$w$	0.30	–	–	–	0.30	–	–	–
${c}_{1}$	1	–	–	–	1	–	–	–
${c}_{2}$	2	–	–	–	2	–	–	–
$z$	–	–	0.20	–	–	–	0.20	–
$AFAD$	–	–	–	0.20	–	–	–	0.20
$P$	–	–	–	0.50	–	–	–	0.50
${n}_{p}$	10	10	10	10	10	10	10	10
${O}_{w} + {O}_{b}$	121	97	145	145	133	121	157	169
Cost function	RMSE							

For developing ANN-PSO hybrid models, first of all, the ANN was initialized using RMSE as a fitness function, and then the PSO algorithm was deployed for optimizing hyperparameters of the ANN. After that, ANN was initialized with 10 input neurons, 10 neurons in the hidden layer, and one output neuron for modelling the *P*_s_-ES. On the contrary, for *UCS*-ES modelling, 11 neurons were used in the hidden layer to constitute 121 and 133 weights and biases for *P*_s_*UCS*-ES models, respectively. The optimum hyperparameters for PSO were set equal to 0.30, 1, and 2 as inertial weight (*w*), social coefficient (*c*_1_), and acceleration coefficient (*c*_2_), respectively.

In the case of ANN-GWO hybrid models, the wolf group was kept equal to 50 individuals. The number of inputs, hidden, and output neurons were adopted such that 97 and 121 weights and biases were obtained in the case of *P*_s_*UCS*-ES models. Based on the hidden neurons, the number of optimized weights as well as biases in the case of ANN-SMA and ANN-MPA are 145 and 157 for *P*_s_-ES models whereas, 145 and 169 for *UCS*-ES models, respectively. The deterministic parameter “*z*” for the ANN-SMA was adopted as 0.20, whereas for ANN-MPA, Fish Aggregating Device (FAD) and *P* were set as 0.20 and 0.50, respectively, as listed in Table 3. Note that, the process for training the metaheuristic model is identical; however, the values of weights as well as biases in the case of the developed model are not the same in each case.

**Figure 12:**
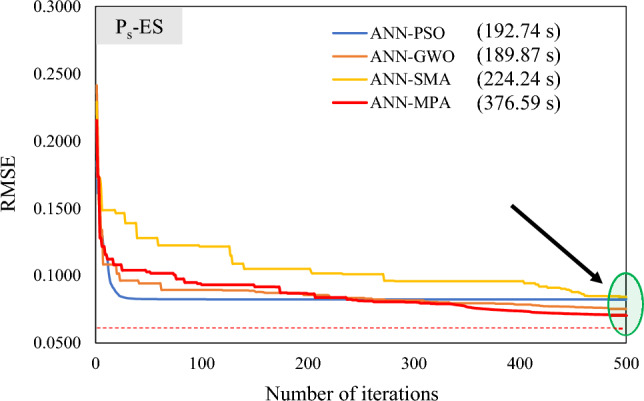
Convergence curves of hybrid ANN models in estimating *P*_*s*_-ES.

**Figure 13:**
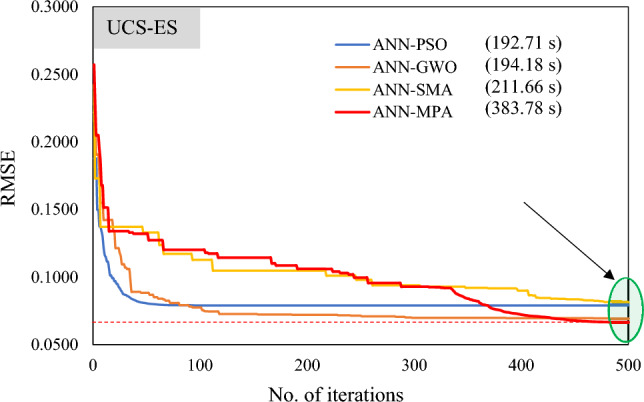
Convergence curves of hybrid ANN models in estimating *UCS*-ES.

The convergence of the algorithm in searching local optima may be trapped; therefore, it is essential to investigate the merging behaviour of the optimization algorithm in assessing the robustness of the developed model. Furthermore, Fig. 12 as well as Fig. 13 display the convergence curves in the case of developed hybrid models (*P*_s_-ES and *UCS*-ES, respectively). It is evident that ANN-PSO and ANN-GWO converge faster (almost equivalent) as compared to the other models, however, ANN-MPA surpasses other models in achieving higher accuracy. It is because the percent difference between ANN-PSO as well as ANN-GWO models is merely 1.5%, in contrast to the 15.11% and 64.58% difference in the case of ANN-SMA as well as ANN-MPA hybrid models, respectively. Moreover, the computational cost for the developed models using MATLAB was observed as 192.74 s, 189.87 s, 224.24 s, and 376.59 s in the case of ANN-PSO, ANN-GWO, ANN-SMA, as well as ANN-MPA, respectively, for 500 iterations of *P*_s_-ES models. Similarly, for *UCS*-ES models, these values were recorded as 192.71 s, 194.18 s, 211.66 s, and 383.78 s, respectively. It is also stated that the number of iterations were finalized for the sake of comparison and this is why the local results were only derived. The curves show that further iterations may not significantly alter the accuracy of formulated models.

### Performance evaluation of the formulated models

This portion evaluates the accuracy analysis of the formulated models by the statistical evaluation equations (Table 4 and Table 5)^104^. The performance evaluation of *TrD* is presented. The performance level for the developed models of *P*_s_-ES was recorded in the range of 79.54% (*R*^2^ = 0.7954) to 85.4% (*R*^2^ = 0.854) in terms of coefficient of determination. Similarly, the *UCS*-ES models yielded an accuracy of 80.07% (*R*^2^ = 0.8007) to 86.22% (*R*^2^ = 0.8622). The *TrD* of both the developed models manifested a correlation (*R* greater than 0.8 which reflects a strong fit to the observed data points^105,106^. The results of the ANN-MPA and ANN-GWO (for *P*_s_-ES), and ANN-MPA (for *UCS*-ES) were found to have *R*^2^ exceeding 0.80, and therefore they are considered to be yielding the best performance, i.e., low error indices. On the contrary, the ANN-PSO and ANN-SMA were observed to yield comparatively lower values while computing the swell-strength characteristics of the ES. The best *R*^2^ values in the case of the ANN and ANN-MPA modelling can be summarized as: (*R*^2^_train_ of ANN = 0.864 and 0.9409, *R*^2^_train_ of ANN-MPA = 0.8541 and 0.8624, and *R*^2^_test_ of ANN = 0.7832 and 0.7921, *R*^2^_test_ of ANN-MPA = 0.8796 and 0.8799). Furthermore, overfitting can be observed in ANN modelling of the *P*_s_*UCS*-ES where the testing *R*^2^ in both the cases is below 0.8. However, this issue is refined and the results have having higher degree of accuracy in ANN-MPA modelling where the training and testing *R*^2^ are almost equivalent.

**Table 4:** Details of performance indices for *P*_*s*_-ES during ANN-based modelling.

Phase	Models	MAE	NS	P_i_	*R*^2^	RMSE	RSR	VAF	WI	WMAPE
Training	ANN-PSO	0.0643	0.8035	1.5075	0.8044	0.0824	0.4433	80.3745	0.9415	0.2366
	ANN-GWO	0.0597	0.8337	1.5760	0.8337	0.0758	0.4078	83.3678	0.9528	0.2185
	ANN-SMA	0.0671	0.7950	1.4872	0.7954	0.0842	0.4528	79.5040	0.9390	0.2459
	ANN-MPA	0.0563	0.8540	1.6234	0.8541	0.0710	0.3820	85.4044	0.9592	0.2060
Testing	ANN-PSO	0.0720	0.8023	1.4096	0.8197	0.0858	0.4446	80.4509	0.9355	0.2731
	ANN-GWO	0.0624	0.8494	1.5655	0.8678	0.0749	0.3881	86.7016	0.9572	0.2365
	ANN-SMA	0.0805	0.7298	1.2156	0.7512	0.1003	0.5198	74.2384	0.9096	0.3050
	ANN-MPA	0.0568	0.8680	1.6045	0.8826	0.0701	0.3633	87.5894	0.9610	0.2153
Validation	ANN-PSO	0.0654	0.7798	1.3209	0.7805	0.0826	0.4693	77.9871	0.9363	0.2736
	ANN-GWO	0.0606	0.8251	1.4708	0.8322	0.0736	0.4182	83.2136	0.9503	0.2536
	ANN-SMA	0.0702	0.7582	1.2880	0.7735	0.0866	0.4917	76.2765	0.9197	0.2937
	ANN-MPA	0.0548	0.8594	1.5947	0.8766	0.0660	0.3750	87.2192	0.9589	0.2294

**Table 5:** Details of performance indices for *UCS*-ES during ANN-based modelling.

Phase	Models	MAE	NS	P_i_	*R*^2^	RMSE	RSR	VAF	WI	WMAPE
Training	ANN-PSO	0.0649	0.8065	1.5162	0.8090	0.0790	0.4398	80.7446	0.9469	0.3546
	ANN-GWO	0.0542	0.8508	1.6171	0.8520	0.0693	0.3862	85.0872	0.9572	0.2961
	ANN-SMA	0.0648	0.7962	1.4937	0.8007	0.0810	0.4514	79.6233	0.9373	0.3542
	ANN-MPA	0.0511	0.8622	1.6427	0.8624	0.0666	0.3712	86.2230	0.9616	0.2796
Testing	ANN-PSO	0.0914	0.5997	0.8034	0.6285	0.1133	0.6327	62.5916	0.8771	0.5025
	ANN-GWO	0.0964	0.4382	0.5304	0.5622	0.1343	0.7496	50.0359	0.8513	0.5300
	ANN-SMA	0.0903	0.5678	0.6644	0.5828	0.1178	0.6574	57.8717	0.8632	0.4962
	ANN-MPA	0.0567	0.8494	1.5142	0.8608	0.0695	0.3881	84.9584	0.9537	0.3116
Validation	ANN-PSO	0.0564	0.7665	1.3132	0.7912	0.0767	0.4832	78.8467	0.9367	0.3539
	ANN-GWO	0.0554	0.8209	1.4346	0.8289	0.0672	0.4231	82.8458	0.9486	0.3339
	ANN-SMA	0.0566	0.8180	1.4143	0.8222	0.0677	0.4266	82.1505	0.9501	0.3531
	ANN-MPA	0.0361	0.8963	1.6551	0.8990	0.0511	0.3220	89.8947	0.9719	0.2216

The values of MAE were calculated in the range of 5.63% to 6.71% and 5.11% to 6.49% for the *TrD* of *P*_s_-ES and *UCS*-ES models, respectively. RMSE values were recorded in the acceptable range of 7.10% to 8.42% and 6.66% to 8.1% for *P*_s_-ES and *UCS*-ES models, respectively. The results reveal that ANN-MPA outperforms other models from the viewpoint of correlation as well as accuracy. The maximum values of *R*^2^ were obtained for the ANN-MPA as 0.854 and 0.8624 for *P*_s_*UCS*-ES models, respectively. Moreover, the lowest MAE (5.63% and 5.11%) and RMSE (7.10% and 6.66%) were also obtained for *P*_s_*UCS*-ES, respectively, in the case of ANN-MPA models. Apart from correlation and mentioned errors, the models were also evaluated using the Nash–Sutcliffe (NS) performance index. The values for NS (in ANN-MPA models) were recorded in the range of 0.79 to 0.8622, with the maximum value of 0.854 and 0.8622 for *P*_s_*UCS*-ES, respectively. The values of NS > 0.75 are found to yield excellent performance. Hence, the currently developed models also manifest strong goodness of fit.

**Figure 14:**
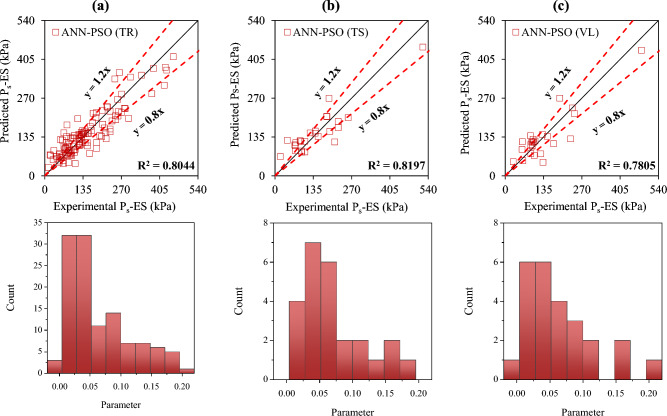
Illustration of performance through scatter plots and error histograms (ANN-PSO of *P*_*s*_-ES prediction).

**Figure 15:**
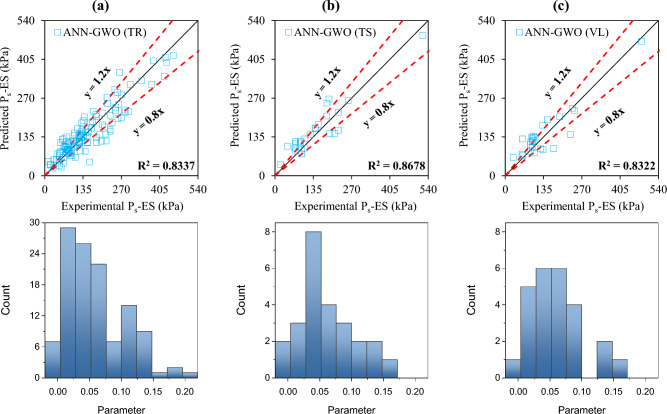
Illustration of performance through scatter plots and error histograms (ANN-GWO of *P*_*s*_-ES prediction).

**Figure 16:**
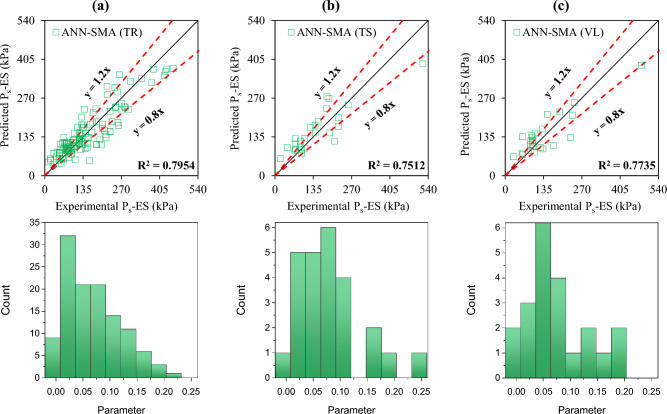
Illustration of performance through scatter plots and error histograms (ANN-SMA of *P*_*s*_-ES prediction).

**Figure 17:**
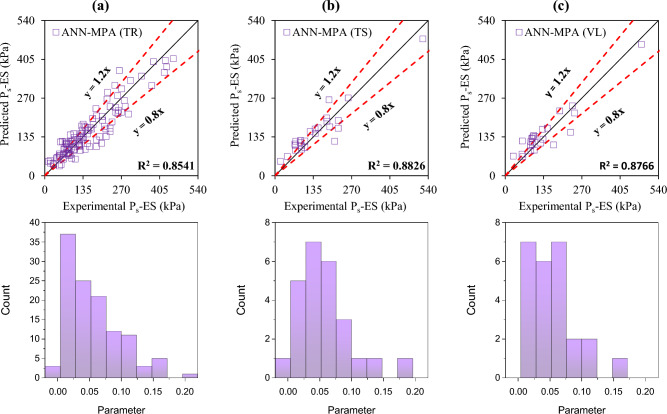
Illustration of performance through scatter plots and error histograms (ANN-MPA of *P*_*s*_-ES prediction).

**Figure 18:**
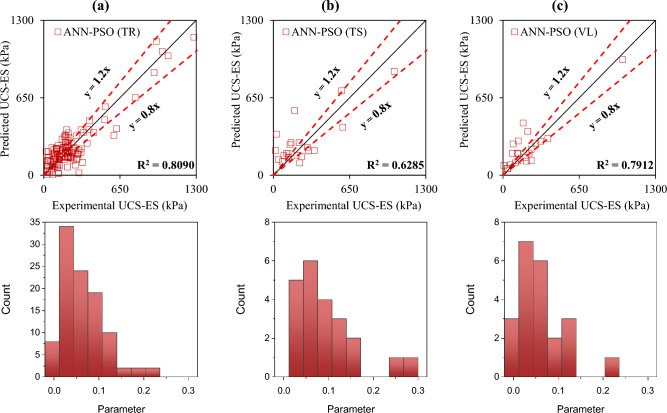
Illustration of performance through scatter plots and error histograms (ANN-PSO of *UCS*-ES prediction).

**Figure 19:**
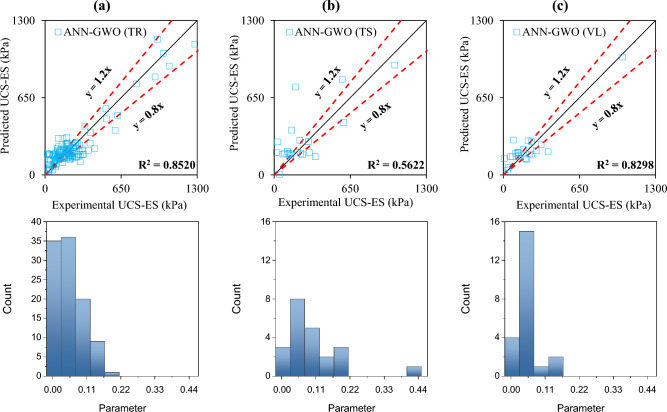
Illustration of performance through scatter plots and error histograms (ANN-GWO of *UCS*-ES prediction).

**Figure 20:**
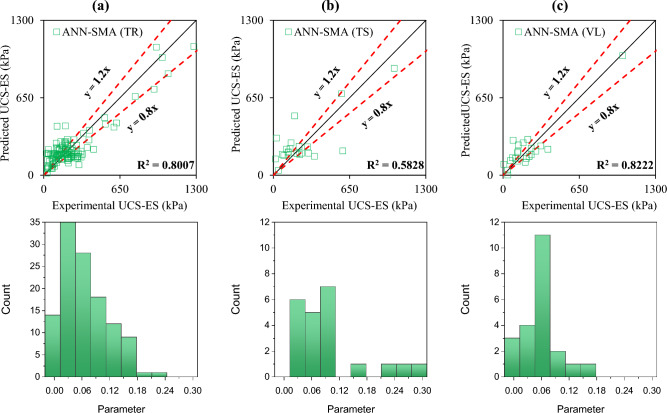
Illustration of performance through scatter plots and error histograms (ANN-SMA of *UCS*-ES prediction).

**Figure 21:**
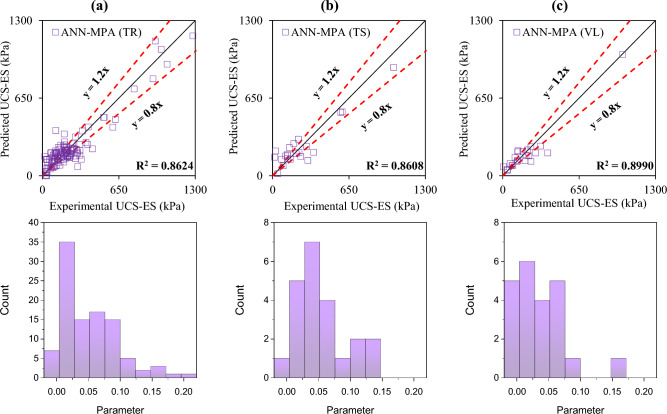
Illustration of performance through scatter plots and error histograms (ANN-MPA of *UCS*-ES prediction).

The accuracy of the formulated models was also evaluated with the help of an error histogram and slope of the regression line obtained using the plot of experimental to predicted results, as shown in Figs. 14, 15, 16, and 17 (*P*_s_-ES) and Figs. 18, 19, 20, as well as Fig. 21 (*UCS*-ES), respectively. It is evident that the scatter of data points for all the developed models mainly lies within the slope of ± 20% deviation from the best-fit line, which also represents the close agreement of predicted and actual results^31^. The error histogram showed 78%, 88%, 82%, and 85% of the *TrD* of *P*_s_-ES models within ± 10% relative error for ANN-PSO, ANN-GWO, ANN-SMA, and ANN-MPA, respectively. Similarly, *UCS*-ES models yielded 85%, 90%, 78%, and 89% of the predictions within ± 10% relative error for ANN-PSO, ANN-GWO, ANN-SMA, as well as ANN-MPA, respectively.

**Figure 22:**
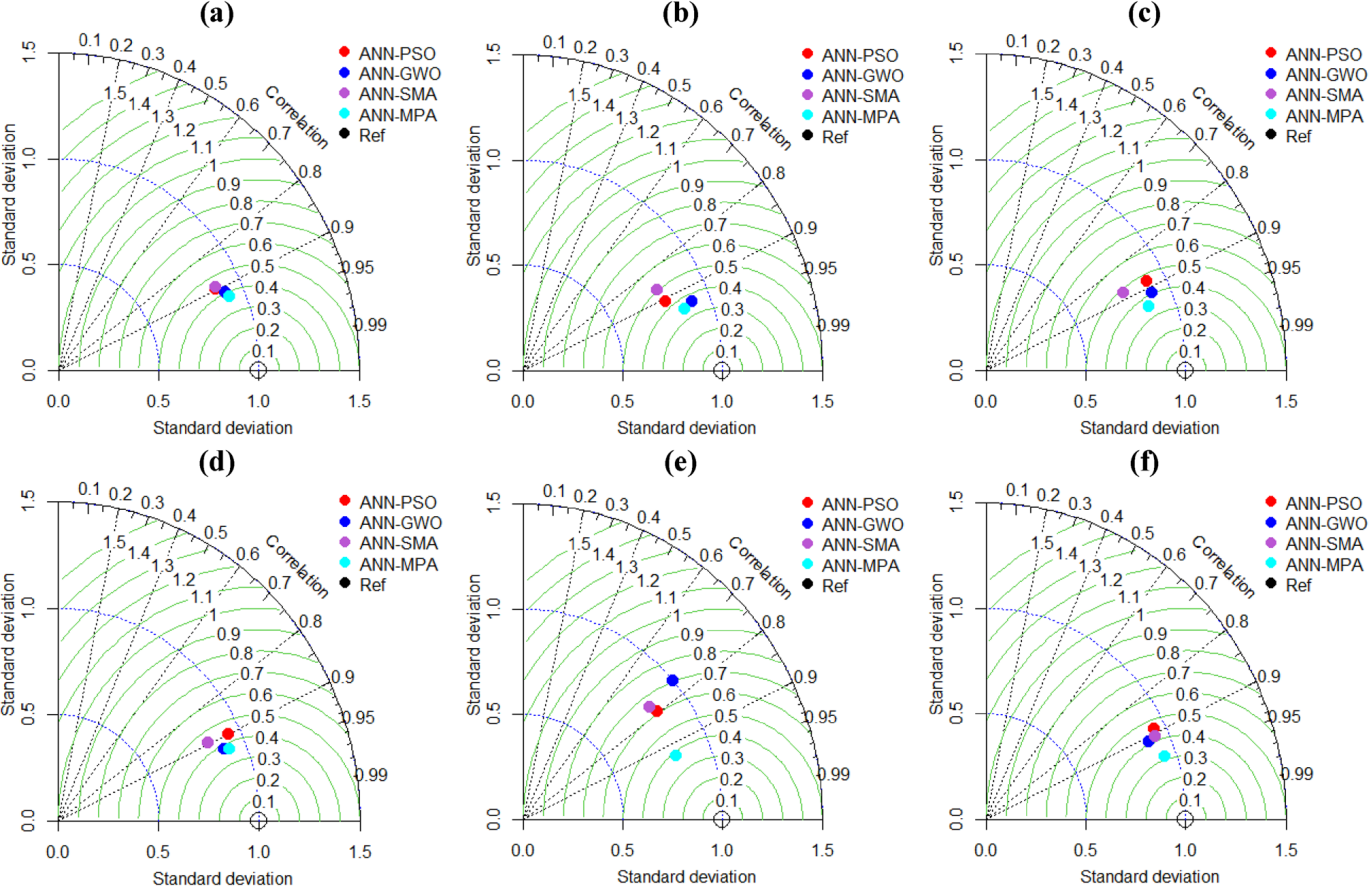
Taylor diagrams: (**a**–**c**) for *P*_*s*_-ES modelling and (**d**–**f**) for *UCS*-ES modelling.

**Figure 23:**
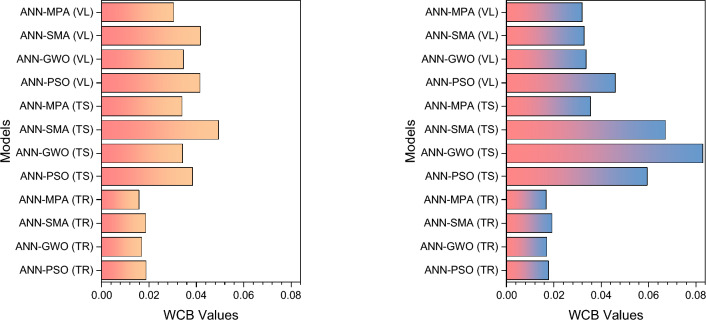
Illustration of WCB values: (**a**) for *P*_*s*_-ES modelling and (**b**) for *UCS*-ES modelling.

Furthermore, a few other visual representations, such as Taylor diagrams as well as the Accuracy matrix, are also given to assess the performance of the formulated ANN-based models. The former refers to the mathematical 2-D representation of the comparative evaluation of the model from the standpoint of root mean squared error (RMSE), *R* (between predicted and experimental values), and the ratio of their standard deviation. Each model is identified within the diagram by a marker, character, or point, which quantifies its evaluation on a linear and radial scale. The position of the marker depicts the model performance; the closer the marker is to the reference point, the higher the accuracy of the developed model. Figure 22 manifests *P*_s_-ES models with *R* values > 0.8, representing a strong agreement among observed as well as predicted values. The correlation values for *UCS*-ES models are also ≥ 0.78, depicting a good fit to experimental results (Fig. 23). The marker points of almost all the models are in proximity to reference points, however, the ANN-MPA being the closest one, represents a relatively more robust model.

**Figure 24:**
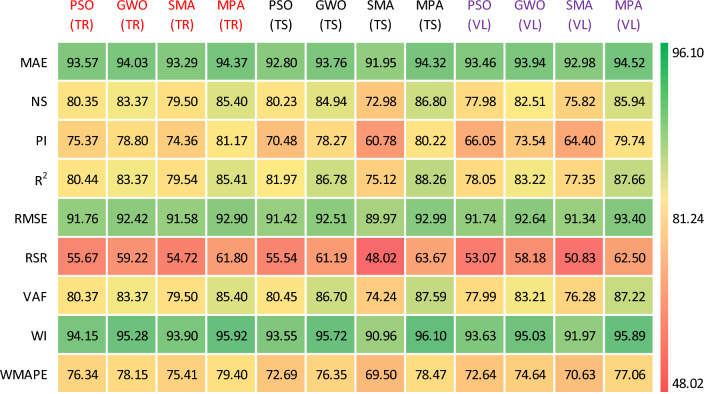
Accuracy matrix for the hybrid ANN models in predicting *P*_*s*_-ES.

**Figure 25:**
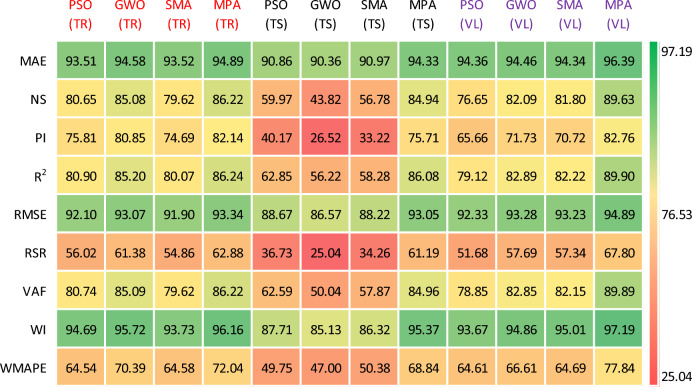
Accuracy matrix for the hybrid ANN models in predicting *UCS*-ES.

For evaluating the accuracy of the formulated models, the accuracy matrix is also presented in Figs. 24 and 25. The percentage accuracy of the model is expressed in terms of ρ relative to their ideal values.

For instance, ideal values for mean absolute error (MAE), RMSE, and *R*^2^ are 0, 0 and 1, respectively. Table 4 shows MAE, RMSE, and *R*^2^ for the ANN-MPA *P*_s_-ES model observed as 0.0563, 0.0710, and 0.8541, respectively. Hence, the accuracy of the ANN-MPA is 94.37% (100–5.63), 92.51% (100–7.10) and 85.41% from the viewpoint of MAE, RMSE, and *R*^2^, respectively. Correspondingly, the accuracy of the ANN-MPA model in the case of *UCS*-ES approaches 94.89%, 93.34%, and 86.24% in terms of MAE, RMSE, and *R*^2^, respectively.

### Validation of the developed models

The attainment of higher accuracy of the *VdD* indicates a more robust and accurate model. Therefore, in this study, the developed models were validated with the help of two levels of validation. Firstly, 30% of the unused data separated from the main dataset was divided equally among *TsD* and *VdD*. In the second level of validation, a simulated dataset was used for parametric analysis, which is presented to see the effect of variable change and its impact on the *P*_s_*UCS*-ES.

#### First level validation

A portion of the primary dataset was used in K-fold cross-validation having K = 5 to validate the ANN-based formulated models. The statistical evaluation of all the proposed models is furnished in Tables 4 and 5 for *P*_s_*UCS*-ES models, respectively. The results reveal that ANN-MPA manifest a more robust model, yielding *R*^2^ = 0.8826, RMSE = 0.0701, and MAE = 0.0568 and *R*^2^ = 0.8766, RMSE = 0.066, as well as MAE = 0.0548 for *TsD* and *VdD* respectively, for predicting *P*_s_-ES. Similarly, ANN-MPA manifested *R*^2^ = 0.8608, RMSE = 0.0695, as well as MAE = 0.0567 for test data and *R*^2^ = 0.8990, RMSE = 0.0511, and MAE = 0.0361 for *VdD*, in the case of *UCS*-ES model. It is pertinent to mention that the magnitudes of correlation are greater, whereas the magnitude of errors for the test and *VdD* lies below the *TrD*, which represents no overfitting during the training stage of the ANN-MPA. Figure 14b,c also depicts that most of the prediction of the ANN-MPA lies in between ± 20% of the deviation of the best-fit line. In the case of *P*_s_-ES models, the performance of other models is depicted in Figs. 14, 15, 16, and 17, which reflects that the accuracy of the formulated models is equivalent to ANN-MPA. ANN-GWO furnished second robust results in forecasting the *P*_s_-ES, whereas, the *UCS*-ES prediction exhibited overfitting in the training process (Figs. 18, 19, 20, and 21, respectively).

#### Uncertainty and statistical testing

The credibility evaluation of a typical AI model is necessary in the case of a prediction model to estimate the target variable for the new dataset. The current study employed UA to evaluate the quantifiable assessment of errors of the developed models to predict *P*_s_*UCS*-ES. This analysis was performed on 1^st^ level of validation, i.e., on the *TrD*, *TsD*, and *VdD*, including 118, 25 and 25 for *P*_s_-ES and 101, 22, and 22 experimental results for *UCS*-ES, as listed in Tables 6 and 7, respectively. To perform the UA, an absolute error was initially calculated between the predicted and experimental values for all three datasets. Subsequently, the mean of error (MOE) and standard deviation (SD) were computed for the said data. Furthermore, the margin of error (ME) was determined at a 95% confidence interval to yield the width of confidence bound (WCB). Upper bound (UB), lower bound (LB), as well as standard error (SE) were also determined to compute WCB. The results of WCB for the formulated models have been provided in Tables 8 and 9 for *P*_s_-ES and *UCS*-ES, respectively.

**Table 6:** Results of Uncertainty analysis (UA) for *P*_*s*_-ES during ANN-based modelling.

Phase	Models	N	MORE	SD	SE	ME	LB	WEB	WCB	Rank
Training	ANN-PSO	118	0.0643	0.0516	0.0048	0.0094	0.0549	0.0737	0.0188	4
	ANN-GWO	118	0.0597	0.0467	0.0043	0.0085	0.0512	0.0682	0.0170	2
	ANN-SMA	118	0.0671	0.0508	0.0047	0.0093	0.0578	0.0764	0.0186	3
	ANN-MPA	118	0.0563	0.0432	0.0040	0.0079	0.0484	0.0642	0.0158	1
Testing	ANN-PSO	25	0.0720	0.0466	0.0093	0.0192	0.0528	0.0912	0.0384	3
	ANN-GWO	25	0.0624	0.0414	0.0083	0.0171	0.0453	0.0795	0.0342	2
	ANN-SMA	25	0.0805	0.0599	0.0120	0.0247	0.0558	0.1052	0.0494	4
	ANN-MPA	25	0.0568	0.0411	0.0082	0.0170	0.0398	0.0738	0.0340	1
Validation	ANN-PSO	25	0.0654	0.0505	0.0101	0.0208	0.0446	0.0862	0.0416	3
	ANN-GWO	25	0.0606	0.0418	0.0084	0.0173	0.0433	0.0779	0.0346	2
	ANN-SMA	25	0.0702	0.0507	0.0101	0.0209	0.0493	0.0911	0.0418	4
	ANN-MPA	25	0.0548	0.0368	0.0074	0.0152	0.0396	0.0700	0.0304	1

**Table 7:** Results of Uncertainty analysis (UA) for *UCS*-ES during ANN-based modelling.

Phase	Models	N	MOE	SD	SE	ME	LB	UB	WCB	Rank
Training	ANN-PSO	101	0.0649	0.0450	0.0045	0.0089	0.0560	0.0738	0.0178	3
	ANN-GWO	101	0.0542	0.0433	0.0043	0.0085	0.0457	0.0627	0.0170	2
	ANN-SMA	101	0.0648	0.0487	0.0048	0.0096	0.0552	0.0744	0.0192	4
	ANN-MPA	101	0.0511	0.0427	0.0042	0.0084	0.0427	0.0595	0.0168	1
Testing	ANN-PSO	22	0.0914	0.0670	0.0143	0.0297	0.0617	0.1211	0.0594	2
	ANN-GWO	22	0.0964	0.0935	0.0199	0.0415	0.0549	0.1379	0.0830	4
	ANN-SMA	22	0.0903	0.0757	0.0161	0.0336	0.0567	0.1239	0.0672	3
	ANN-MPA	22	0.0567	0.0402	0.0086	0.0178	0.0389	0.0745	0.0356	1
Validation	ANN-PSO	22	0.0564	0.0519	0.0111	0.0230	0.0334	0.0794	0.0460	4
	ANN-GWO	22	0.0554	0.0379	0.0081	0.0168	0.0386	0.0722	0.0336	3
	ANN-SMA	22	0.0566	0.0371	0.0079	0.0164	0.0402	0.0730	0.0328	2
	ANN-MPA	22	0.0361	0.0361	0.0077	0.0160	0.0201	0.0521	0.0320	1

**Table 8:** Results of one-tailed t-test for *P*_*s*_-ES during ANN-based modelling.

Phase	Models	N	of	HMD	t-stat	t critical one-tail	H_0_
Training	ANN-PSO	118	117	0	− 2.2522	1.6580	Reject
	ANN-GWO	118	117	0	− 1.2375	1.6580	Reject
	ANN-SMA	118	117	0	− 3.3396	1.6580	Reject
Testing	ANN-PSO	25	24	0	− 2.4730	1.7109	Reject
	ANN-GWO	25	24	0	− 0.7373	1.7109	Reject
	ANN-SMA	25	24	0	− 2.2813	1.7109	Reject
Validation	ANN-PSO	25	24	0	− 1.1068	1.7109	Reject
	ANN-GWO	25	24	0	− 1.1615	1.7109	Reject
	ANN-SMA	25	24	0	− 1.8492	1.7109	Reject

**Table 9:** Results of one-tailed t-test for *UCS*-ES during ANN-based modelling.

Phase	Models	N	of	HMD	t-stat	t critical one-tail	H_0_
Training	ANN-PSO	101	100	0	− 3.3676	1.6602	Reject
	ANN-GWO	101	100	0	− 1.0312	1.6602	Reject
	ANN-SMA	101	100	0	− 4.0629	1.6602	Reject
Testing	ANN-PSO	22	21	0	− 2.8806	1.7207	Reject
	ANN-GWO	22	21	0	− 2.0476	1.7207	Reject
	ANN-SMA	22	21	0	− 2.4352	1.7207	Reject
Validation	ANN-PSO	22	21	0	− 1.7820	1.7207	Reject
	ANN-GWO	22	21	0	− 2.1390	1.7207	Reject
	ANN-SMA	22	21	0	− 2.3862	1.7207	Reject

#### Second level validation

The value of WCB for a good model shall be as small as possible; hence, the model with minimum WCB reflects a robust model.

For both cases, *P*_s_-ES and *UCS*-ES, the ANN-MPA manifested minimum WCB, therefore, it ranked first in robustness for *TrD*, *TsD*, and *VdD* data, which is also depicted in Fig. 23.

### Second-level validation

**Figure 26:**
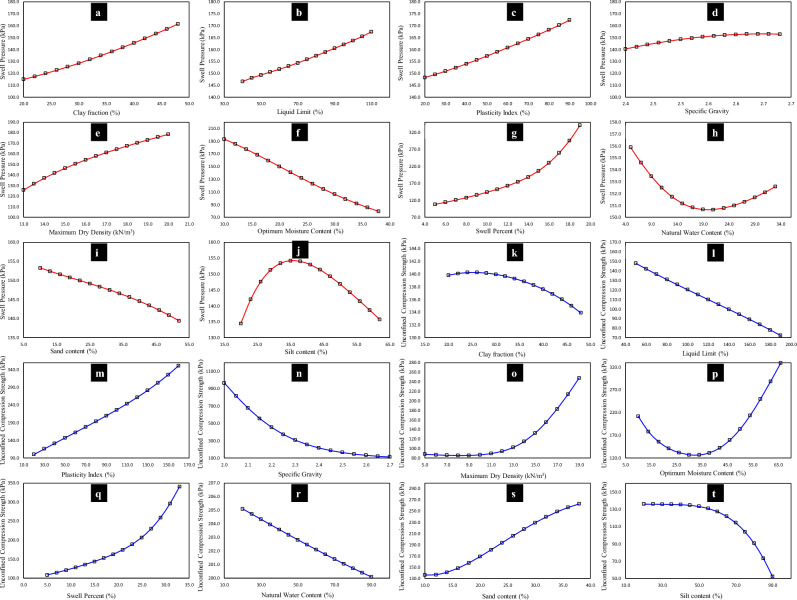
Illustration of level-2 validation phase.

Owing to the overfitting problem while formulation of AI models, the models generated in this study were validated on different sets. For this purpose, simulated datasets were created as shown in Table 10. Moreover, as depicted in Fig. 26 the effect of changing parameters has been studied by keeping remaining variables constant. The details of the parametric and sensitivity analysis are given below.

**Table 10:** Details of simulated datasets for *P*_*s*_*UCS*-ES for validation purposes.

Variable input parameters			Data points	Constant input parameters for *P*_*s*_ and *UCS*, respectively
Parameter	*P*_*s*_ Range	*UCS* Range		
*CF*	20–48	20–48	15	*LL* = 64.03,71.59 *PI* = 36.19,42.62 G_s_ = 2.69,2.59 *MDD* = 15.78,15.17 *OMC* = 19.45,24.11 *SP* = 7.23,13.04 *w*_n_ = 7.44,32.71 S: 11.84,10.82 M: 41.50,38.30
*LL*	40–110	50–190	15	*CF* = 43.77,44.97 *PI* = 36.19,42.62 G_s_ = 2.69,2.59 *MDD* = 15.78,15.17 *OMC* = 19.45,24.11 *SP* = 7.23,13.04 *w*_n_ = 7.44,32.71 S: 11.84,10.82 M: 41.50,38.30
*PI*	20–90	20–160	15	*CF* = 43.77,44.97 *LL* = 64.03,71.59 G_s_ = 2.69,2.59 *MDD* = 15.78,15.17 *OMC* = 19.45,24.11 *SP* = 7.23,13.04 *w*_n_ = 7.44,32.71 S: 11.84,10.82 M: 41.50,38.30
*G*_s_	2.4–2.68	2–2.7	15	*CF* = 43.77,44.97 *LL* = 64.03,71.59 *PI* = 36.19,42.62 *MDD* = 15.78,15.17 *OMC* = 19.45,24.11 *SP* = 7.23,13.04 *w*_n_ = 7.44,32.71 S: 11.84,10.82 M: 41.50,38.30
*MDD*	13–20	5–19	15	*CF* = 43.77,44.97 *LL* = 64.03,71.59 *PI* = 36.19,42.62 G_s_ = 2.69,2.59 *OMC* = 19.45,24.11 *SP* = 7.23,13.04 *w*_n_ = 7.44,32.71 S: 11.84,10.82 M: 41.50,38.30
*OMC*	10–38	10–66	15	*CF* = 43.77,44.97 *LL* = 64.03,71.59 *PI* = 36.19,42.62 G_s_ = 2.69,2.59 *MDD* = 15.78,15.17 *SP* = 7.23,13.04 *w*_n_ = 7.44,32.71 S: 11.84,10.82 M: 41.50,38.30
*SP*	5–19	5–33	15	*CF* = 43.77,44.97 *LL* = 64.03,71.59 *PI* = 36.19,42.62 G_s_ = 2.69,2.59 *MDD* = 15.78,15.17 *OMC* = 19.45,24.11 *w*_n_ = 7.44,32.71 S: 11.84,10.82 M: 41.50,38.30
*w*_n_	5–33	20–90	15	*CF* = 43.77,44.97 *LL* = 64.03,71.59 *PI* = 36.19,42.62 G_s_ = 2.69,2.59 *MDD* = 15.78,15.17 *OMC* = 19.45,24.11 *SP* = 7.23,13.04 S: 11.84,10.82 M: 41.50,38.30
*sand*	10–52	10–38	15	*CF* = 43.77,44.97 *LL* = 64.03,71.59 *PI* = 36.19,42.62 G_s_ = 2.69,2.59 *MDD* = 15.78,15.17 *OMC* = 19.45,24.11 *SP* = 7.23,13.04 *w*_n_ = 7.44,32.71 M: 41.50,38.30
*silt*	20–62	20–90	15	*CF* = 43.77,44.97 *LL* = 64.03,71.59 *PI* = 36.19,42.62 G_s_ = 2.69,2.59 *MDD* = 15.78,15.17 *OMC* = 19.45,24.11 *SP* = 7.23,13.04 *w*_n_ = 7.44,32.71 S: 11.84,10.82

#### Parametric analysis

Table 10 illustrates the details of the simulated datasets produced alongside the fluctuating range of the considered input parameters^107^. It is pertinent to mention that, the summation of all the input parameters had been 100% the same everywhere to simulate the real-world scenario. Moreover, the *LL*, *PI*, *G*_s_, *MDD*, *OMC*, *SP*, *w*_n_, *sand*, and *silt* were designated at their minimum, maximum, and mean entities.

It is depicted in Fig. 26 that, as anticipated, all the trends are shown by smooth curves. Figure 26a–e,g depict the expected increase in *P*_s_-ES with rising *CF*, *LL*, *PI*, *G*_s_ and *MDD*, respectively, while, Fig. 26f, i–j displays the reverse decreasing trend in *P*_s_-ES with increasing *OMC*, *w*_n_, *sand* and *silt*, respectively. These results are consistent with the R-value reflected by the given matrix in Fig. 11, as well as they are in good agreement with the findings of Jalal et al.^31^. However, the decrease in the *P*_s_-ES at higher water content is associated with larger values of *P*_s_-ES with *LL* (Fig. 26b), which is reflected by the Δw = 0.6(PI/LL)^108^. On the contrary, the forecasted *UCS*-ES elevated with increasing *PI*, *MDD*, *OMC*, and *SP*, as shown in Fig. 26m–q, while, it lowered down in the case of G_s_ and *silt* content, Fig. 26n,t, respectively, which are are in good agreement with the GEP parametric study results of Jalal et al.^31^. But, for *OMC* and *SP*, the *P*_s_-ES was observed to follow an increasing trend after some time since the *OMC* and *MDD* are significantly influenced by the particle size being fine. It is stated that the greater impact is by the content of *CF*^109^. Figure 26k–l shows that the *UCS*-ES lowered down with the increase in *CF* and *LL* of the original ES^110^. Figure 26r showed that with the increase in soil water content, the *UCS*-ES was observed to decrease^111^. Also, the trends between swell-strength characteristics and the nine aforementioned input parameters attained in the level-2 validation stage are in good agreement with the behaviour of the actual dataset (as shown in Figs. 7 and 8, respectively), which verifies the robustness of the proposed model.

#### Sensitivity analysis

Sensitivity analysis (SA) evaluates the impact on the output of a formulated model with changing input parameters. It gives an idea about the most significant input parameters, and as a result, by eliminating the relatively trivial parameters, the number of inputs could be lessened, thereby lowering the perplexity of the model alongside the time required for training a specific model. To conduct the SA for the current study on *P*_s_*UCS*-ES, the generally employed cosine amplitude technique (referred to as, CAM) was incorporated wherein the data pairs assist in the construction of data array, = [x_1_,x_2_,x_3_,…, x_i_,…,x_n_], such that the variable x_i_ in the array, X, refers to the length vector of m in the form of:

60 $$ x_{i} = [x_{i1} ,x_{i2} ,x_{i3} ,...,x_{im} ] $$

The association among A_ij_ (strength of the relation) versus the datasets of x_i_ as well as x_j_ is determined with the help of Eq. (61):

61 $$ A_{ij} = \frac{{\sum\nolimits_{k = 1}^{m} {x_{ik} x_{jk} } }}{{\sum\nolimits_{k = 1}^{m} {x^{2} ik\sum\nolimits_{k = 1}^{m} {x_{ik}^{2} } } }} $$

**Figure 27:**
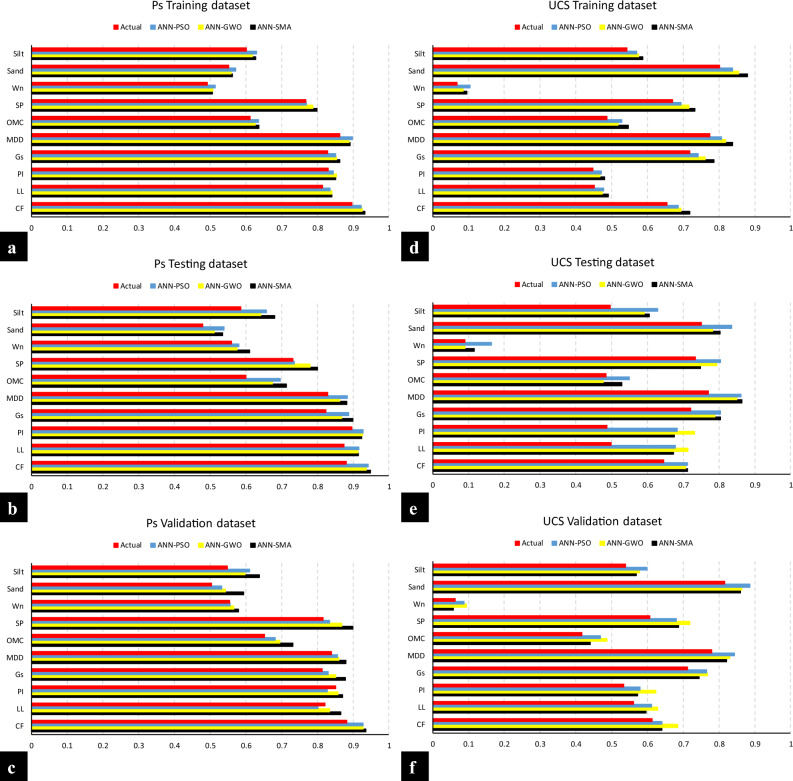
Sensitivity analysis of *P*_*s*_*UCS*-ES.

The A_ij_ values for *P*_s_*UCS*-ES versus the input parameters are depicted in Fig. 27. In the *TrD* of *P*_s_-ES, the *CF* and *MDD* are the governing parameters whose effect exceeds 0.90 whereas the w_n_, *sand* and *silt* have the lowest impact on the *P*_s_-ES. The results of ANN-SMA and ANN-PSO are higher than those of ANN-GWO for all studied input parameters except *PI* and SP. On the contrary, in the *TrD*, *TsD*, and *VdD* of *UCS*-ES, the *MDD* and *sand* appear to largely govern the strength of the ES. In the *TrD* of *UCS*-ES, the effect of *PI*, *OMC*, and *w*_n_ is recorded to be the least, respectively. Furthermore, the efficiency of results with various algorithms in the *TrD* of *UCS*-ES case follows the order: of ANN-SMA > ANN-GWO > ANN-PSO. Similarly, in the case of *TsD* and *VdD* of *P*_s_-ES, *CF* and *PI* are the most significant input parameters whereas w_n_, *sand* and *silt* are the least significant parameters. Interestingly, the efficiency of results is higher for ANN-SMA and ANN-PSO in the case of *TsD* (*P*_s_*UCS*-ES). However, ANN-PSO and ANN-SMA yield the lowest results for *P*_s_*UCS*-ES in the case of *VdD*. Hence, ANN-SMA exhibits the most reliable results for *P*_s_-ES while ANN-SMA and ANN-PSO are equally efficient algorithms in the case of *UCS*-ES.

## Summary and conclusions

In various civil engineering projects, the swell-strength properties of expansive soils (ES) are crucial for evaluating the design of structures resting on the ES. Usually, laboratory tests are conducted for computing the swell pressure as well as the unconfined compression strength of the ES (referred to as ‘*P*_s_*UCS*-ES’) which are not only time-consuming but also expensive. Thus, this study aims to find a robust and efficacious alternative to conduct the actual laboratory tests with efficient AI-based models. This would help to estimate the *P*_s_*UCS*-ES based on available experimental databases from the past literature. This study concentrates on the formulation of metaheuristics by deploying PSO, GWO, SMA, and MPA for the evaluation of the *P*_s_*UCS*-ES obtained from ANN modelling. A database of 168 *P*_s_ and 145 *UCS* observations was considered by consulting 61 and 99 internationally published papers, respectively, after a detailed literature search. 70% of the dataset was selected randomly as the *TrD*, whereas the rest of the unused dataset was deployed to test and validate the developed models. Based on the aforementioned modelling, the following conclusions are drawn:1. All the models were trained using the best hyperparameters of the ANN model resulting from PSO, GWO, SMA, and MPA. In the case of *P*_s_-ES modelling, the fixation of several neurons in the hidden layer is purely a trial-and-error method. Furthermore, the ANN models of *P*_s_*UCS*-ES using PSO were uniformly optimized with inertial weights equalling 0.3, social coefficient of unity, and acceleration coefficient of 2. The ANN-GWO metaheuristic (189.87 s) exhibited superior performance from the standpoint of computational cost, whereas PSO (192.71 s) surpassed in the case of the *UCS*-ES models.

*UCS*2. Validation of the ANN-based *P*_s_*UCS*-ES models using wide statistical indices (such as MAE, NS, ρ, *R*^2^, RMSE, RSR, VAF, WI, and WMAPE) was performed. It was recorded that all the developed models for *P*_s_-ES exhibited *R* significantly exceeding 0.8 for the *TrD*, *TsD*, and *VdD*. However, ANN-MPA excelled in yielding high *R* values and exhibited the lowest absolute error for all these three distinct.

3. The results of *UCS*-ES models performance revealed that *R* only exceeded 0.9 in the case of *TrD*, but, not for *TsD* and *VdD*. Also, the ANN-MPA model yielded higher *R* values (0.89, 0.93, and 0.94), and comparatively low MAE values (5.11%, 5.67, and 3.61%) in the case of PSO, GWO, and SMA, respectively. *UCSUCS.*

4. All the ANN-base models were also tested using the a-20 index. For all the formulated models, maximum points were recorded to lie within ± 20% error. In addition, the ANN-SMA interpreted higher accuracy in terms of the a-20 index, and its superiority was also supported by the results depicted in Taylor’s diagram and the WCB values.

5. The uncertainty analysis UA for *P*_s_-ES models showed that the ANN-MPA is observed to be the most accurate model followed by ANN-GWO, ANN-SMA, and ANN-PSO for the *TrD*. This type of trend was also recorded for the *TsD* and *VdD* except that ANN-PSO outperformed ANN-SMA. On the other hand, in the case of *UCS*-ES models, the ANN-MPA exhibited the highest accuracy followed by ANN-GWO, ANN-PSO, and ANN-SMA, for *TrD*. The parameter and sensitivity analyses of ANN-based *P*_s_*UCS*-ES models also revealed coherent variation of the considered input parameters with the outputs.

This study is limited to the range of the parameters mentioned in the available dataset considered in this paper. Also, the inherent time and cost attributed to the initial creation of the aforementioned experimental database are still challenging. The models formulated here are based on specific soil characteristics and environmental conditions. In addition, the presence of biases or inaccuracies in this database could affect the robustness of the developed models. The validation of these models is also limited to the existing database. Moreover, trial and error in model optimization, overfitting issues, and computational costs are other noteworthy limitations while developing models. It is suggested to evaluate other optimization techniques including random forest and support vector machines in future research.

## Supplementary Information

Supplementary Information.

## Data Availability

The data used in the manuscript may be provided upon requesting Fazal E Jalal (jalal@szu.edu.cn).
