# Author Correction: ANN-based swarm intelligence for predicting expansive soil swell pressure and compression strength

**DOI:** 10.1038/s41598-024-76107-4

**Published:** 2024-11-06

**Authors:** Fazal E. Jalal, Mudassir Iqbal, Waseem Akhtar Khan, Arshad Jamal, Kennedy Onyelowe, Lekhraj

**Affiliations:** 1 State Key Laboratory of Intelligent Geotechnics and Tunnelling, College of Civil and Transportation Engineering, Shenzhen University, Shenzhen, 518060 Guangdong China; 2 Key Laboratory of Coastal Urban Resilient Infrastructures (Shenzhen University), Ministry of Education, Shenzhen, China; 3 Department of Civil Engineering, University of Engineering and Technology Peshawar, Peshawar, Pakistan; 4 Department of Civil Engineering, University of Louisiana at Lafayette, Lafayette, LA 70503 USA; 5 Department of Civil Engineering, College of Engineering, Qassim University, 51452 Buraydah, Saudi Arabia; 6 Department of Civil Engineering, Kampala International University, Kampala, Uganda; 7 Department of Computer Engineering and Applications, GLA University, Mathura, 281406 India

Correction to: *Scientific Reports* 10.1038/s41598-024-65547-7, published online 25 June 2024

In the original version of this article author Arshad Jamal was incorrectly affiliated with ‘Department of Civil and Environmental Engineering, King Fahd University of Petroleum and Minerals, KFUPM, Box 5055, 31261, Dhahran, Saudi Arabia’. The correct affiliation is listed below.

Department of Civil Engineering, College of Engineering, Qassim University, Buraydah 51452, Saudi Arabia.

Also, author Mudassir Iqbal was omitted as a corresponding author. Correspondence and requests for materials should also be addressed to mudassiriqbal@uetpeshawar.edu.pk.

The original Article has been corrected.

