# Supplementary material for: ANN-based swarm intelligence for predicting expansive soil swell pressure and compression strength
Source: Sci Rep. 2024 Jun 25;14:14597. doi: 10.1038/s41598-024-65547-7 (PMC11199650; doi:10.1038/s41598-024-65547-7)
Supplement: Supplementary file 1 — Supplementary Information. [file 41598_2024_65547_MOESM1_ESM.docx]

**Modelling the swell-strength nature of expansive soils by ANN-based swarm intelligence for sustainable construction**

Supplementary Information

Table S1. Experimental database for swell pressure prediction of expansive soils.

Table S2. Experimental database for unconfined compression strength prediction of expansive soils.

|  | Swell pressure, P_s_ | | | | | | | | | | | |
| --- | --- | --- | --- | --- | --- | --- | --- | --- | --- | --- | --- | --- |
| **S. No** | **Reference** | **Clay fraction**  **(CF)** | **Liquid**  **Limit**  **(LL)** | **Plasticity**  **Index**  **(PI)** | **Specific**  **Gravity**  **(G_s_)** | **Max. Dry**  **Density**  **(MDD)** | **Optimum**  **Moisture**  **Content**  **(OMC)** | **Swell**  **Potential**  **(SP)** | **Natural**  **Water**  **Content**  **(w_n_)** | **Sand** | **Silt** | **Swell**  **Pressure**  **(P_s_)** |
|  |  | % | % | % |  | kN/m^3^ | % | % | % | % | % | KPa |
| 1 | [1] | 70 | 31 | 12.9 | 2.7 | 16.8 | 18.4 | 14.9 | 12.1 | 7.0 | 15.0 | 139 |
| 2 | [2] | 65 | 148 | 116 | 2.68 | 14.9 | 23 | 9.3 | 3.7 | 0.0 | 35.0 | 170 |
| 3 | [3] | 72.5 | 74 | 35 | 2.66 | 17.55 | 19 | 5.43 | 2.2 | 4.0 | 23.5 | 270 |
| 4 | [4] | 23 | 78 | 34 | 2.77 | 13.5 | 32 | 0 | 0.0 | 2.0 | 75.0 | 65 |
| 5 |  | 45 | 71 | 28 | 2.75 | 11.7 | 40 | 0 | 0.0 | 19.0 | 24.0 | 107 |
| 6 |  | 55 | 94 | 54 | 2.78 | 15.1 | 28 | 0.6 | 0.2 | 28.0 | 20.0 | 73 |
| 7 |  | 48 | 64 | 40 | 2.77 | 16.6 | 17 | 0.9 | 0.4 | 14.0 | 27.5 | 193 |
| 8 |  | 48 | 59 | 37 | 2.75 | 19 | 7 | 13.3 | 5.3 | 28.0 | 20.0 | 221 |
| 9 | [5] | 26 | 58.5 | 33 | 2.7 | 16.5 | 19 | 2.7 | 19.0 | 22.0 | 27.5 | 75 |
| 10 |  | 58 | 63 | 35.5 | 2.7 | 14.5 | 26 | 1.3 | 26.0 | 4.7 | 36.0 | 89 |
| 11 |  | 32 | 59.5 | 30.5 | 2.7 | 14.5 | 30 | 1.82 | 30.0 | 22.0 | 27.5 | 51 |
| 12 |  | 41 | 64 | 35.5 | 2.7 | 15 | 28.5 | 4.49 | 28.5 | 18.0 | 26.0 | 59 |
| 13 |  | 19 | 43 | 21.5 | 2.68 | 14 | 25 | 3.4 | 25.0 | 13.0 | 67.0 | 29 |
| 14 |  | 43 | 57.5 | 28.5 | 2.69 | 15.5 | 26.5 | 5.05 | 26.5 | 15.0 | 29.0 | 125 |
| 15 | [6] | 69 | 80 | 52 | 2.72 | 13.75 | 40 | 10.8 | 14.0 | 7.0 | 24.0 | 90 |
| 16 | [7] | 27 | 52 | 20 | 2.66 | 17.36 | 9.2 | 2.4 | 9.2 | 34.3 | 31.8 | 215 |
| 17 |  | 26 | 58 | 24 | 2.66 | 17.36 | 8.8 | 2.5 | 8.8 | 34.3 | 31.8 | 144 |
| 18 |  | 61 | 46 | 25 | 2.66 | 17.36 | 12.4 | 5.6 | 12.4 | 4.7 | 36.0 | 283 |
| 19 |  | 32 | 67 | 30 | 2.66 | 17.36 | 8.1 | 4.8 | 8.1 | 28.0 | 20.0 | 204 |
| 20 |  | 73 | 58 | 35 | 2.66 | 17.36 | 4.4 | 6.5 | 4.4 | 2.0 | 24.0 | 302 |
| 21 |  | 34 | 36 | 24 | 2.66 | 17.36 | 5.5 | 3.8 | 5.5 | 22.2 | 34.2 | 237 |
| 22 |  | 71 | 72 | 40 | 2.66 | 17.36 | 3.7 | 7.8 | 3.7 | 2.5 | 22.5 | 341 |
| 23 |  | 61 | 87 | 45 | 2.66 | 17.36 | 6.2 | 6.6 | 6.2 | 2.7 | 38.0 | 247 |
| 24 |  | 21 | 32 | 8 | 2.75 | 17.36 | 8.7 | 1.7 | 8.7 | 14.0 | 31.0 | 123 |
| 25 |  | 93 | 94 | 70 | 2.75 | 17.36 | 2.7 | 10.2 | 2.7 | 1.0 | 6.0 | 480 |
| 26 |  | 91 | 105 | 79 | 2.75 | 17.36 | 2.3 | 10.8 | 2.3 | 1.0 | 8.0 | 521 |
| 27 |  | 41 | 72 | 33 | 2.75 | 17.36 | 9.1 | 4.3 | 9.1 | 19.0 | 24.0 | 223 |
| 28 |  | 33 | 39 | 21 | 2.75 | 17.36 | 7.2 | 3.1 | 7.2 | 14.0 | 27.5 | 223 |
| 29 |  | 84 | 80 | 63 | 2.75 | 17.36 | 4.9 | 9.2 | 4.9 | 4.0 | 14.0 | 425 |
| 30 |  | 77 | 85 | 50 | 2.75 | 17.36 | 4.3 | 7.8 | 4.3 | 4.0 | 19.0 | 263 |
| 31 |  | 53 | 82 | 55 | 2.75 | 17.36 | 5.3 | 7.2 | 5.3 | 12.0 | 30.0 | 257 |
| 32 |  | 58 | 67 | 52 | 2.77 | 17.36 | 2.1 | 8.4 | 2.1 | 7.0 | 33.0 | 377 |
| 33 |  | 84 | 79 | 53 | 2.77 | 17.36 | 2.3 | 9.7 | 2.3 | 5.5 | 13.0 | 383 |
| 34 |  | 71 | 62 | 46 | 2.77 | 17.36 | 1.3 | 8.5 | 1.3 | 4.0 | 23.0 | 421 |
| 35 |  | 44 | 57 | 41 | 2.77 | 17.36 | 5.5 | 5.4 | 5.5 | 12.0 | 28.0 | 249 |
| 36 |  | 79 | 93 | 56 | 2.77 | 17.36 | 2.4 | 10.5 | 2.4 | 6.0 | 15.0 | 430 |
| 37 |  | 48 | 58 | 28 | 2.77 | 17.36 | 7.3 | 4.1 | 7.3 | 8.0 | 40.0 | 182 |
| 38 |  | 49 | 62 | 31 | 2.77 | 17.36 | 5.1 | 5.7 | 5.1 | 28.0 | 20.0 | 244 |
| 39 |  | 91 | 89 | 58 | 2.77 | 17.36 | 3.8 | 8.2 | 3.8 | 1.0 | 7.0 | 453 |
| 40 | [8] | 55 | 44 | 22 | 2.46 | 16.26 | 17.49 | 8.1 | 3.2 | 3.0 | 22.0 | 116 |
| 41 | [9] | 45 | 52 | 34 | 2.81 | 16.5 | 21.5 | 8.2 | 21.6 | 5.4 | 37.4 | 125 |
| 42 | [10] | 27 | 77.2 | 42.5 | 2.73 | 17.2 | 18 | 4.09 | 18.0 | 22.0 | 27.5 | 210 |
| 43 |  | 33 | 56 | 26 | 2.69 | 16.5 | 20 | 3.34 | 20.0 | 8.0 | 40.0 | 170 |
| 44 |  | 31 | 55.4 | 23.9 | 2.7 | 16.8 | 22 | 2.97 | 22.0 | 22.0 | 27.5 | 142 |
| 45 | [11] | 47 | 116 | 75 | 2.66 | 14.9 | 31.4 | 5.68 | 19.1 | 5.0 | 50.1 | 125 |
| 46 |  | 48 | 132 | 80 | 2.67 | 15 | 29.2 | 5.09 | 20.1 | 5.4 | 55.3 | 150 |
| 47 |  | 28.2 | 108 | 61 | 2.64 | 18 | 19.9 | 4.91 | 17.6 | 24.0 | 35.4 | 150 |
| 48 | [12] | 34 | 45.2 | 19.83 | 2.72 | 18.34 | 15 | 5.96 | 2.4 | 23.2 | 36.3 | 142 |
| 49 | [13] | 51 | 59 | 27 | 2.68 | 16.2 | 26.2 | 2.3 | 0.9 | 8.0 | 40.0 | 75 |
| 50 |  | 62 | 54 | 34 | 2.78 | 16.7 | 20.6 | 5.7 | 2.3 | 2.0 | 37.0 | 141 |
| 51 |  | 50 | 70 | 49 | 2.77 | 15.5 | 24.2 | 9.3 | 3.7 | 33.0 | 17.0 | 230 |
| 52 | [14] | 56 | 60 | 28 | 2.61 | 16.1 | 21 | 4.78 | 1.9 | 18.0 | 26.0 | 128 |
| 53 | [15] | 55 | 95 | 70 | 2.65 | 12.94 | 32 | 6.6 | 2.6 | 5.4 | 37.4 | 150 |
| 54 | [16] | 60 | 62 | 37 | 2.7 | 13.1 | 22.1 | 5.4 | 2.2 | 0.0 | 40.0 | 216 |
| 55 | [17] | 43 | 55 | 32 | 2.69 | 15.3 | 22 | 4.5 | 7.0 | 2.0 | 58.0 | 85 |
| 56 | [18] | 60 | 61 | 30 | 2.67 | 16.7 | 20.7 | 5.2 | 2.1 | 12.0 | 28.0 | 132 |
| 57 | [19] | 52 | 57 | 29 | 2.56 | 14.68 | 24 | 4.4 | 1.8 | 8.0 | 40.0 | 200 |
| 58 | [20] | 70 | 85.2 | 52.13 | 2.61 | 15.21 | 24.7 | 20.6 | 8.2 | 2.0 | 28.0 | 295 |
| 59 | [21] | 73 | 98 | 62 | 2.56 | 16.2 | 26 | 12 | 4.8 | 25.0 | 73.0 | 192 |
| 60 |  | 72 | 76 | 46 | 2.6 | 16 | 25 | 15 | 6.0 | 26.0 | 72.0 | 230 |
| 61 |  | 71 | 64 | 38 | 2.58 | 16.4 | 23 | 17 | 6.8 | 26.0 | 71.0 | 280 |
| 62 | [22] | 66 | 69 | 36 | 2.69 | 14.21 | 25.38 | 10.1 | 4.0 | 18.0 | 16.0 | 105 |
| 63 |  | 56 | 61 | 31 | 2.76 | 15.22 | 23.4 | 9.5 | 3.8 | 25.0 | 19.0 | 93 |
| 64 |  | 70 | 72 | 40 | 2.72 | 13.8 | 25.6 | 15 | 6.0 | 10.0 | 20.0 | 130 |
| 65 |  | 57 | 68 | 37 | 2.7 | 14.33 | 24 | 8.7 | 3.5 | 19.0 | 24.0 | 95 |
| 66 |  | 52 | 59 | 30 | 2.66 | 14.5 | 22.7 | 7 | 2.8 | 28.0 | 20.0 | 90 |
| 67 |  | 62 | 74 | 39 | 2.7 | 13.2 | 26.7 | 10.5 | 4.2 | 20.0 | 18.0 | 122 |
| 68 | [23] | 27 | 100 | 50 | 2.69 | 16.18 | 16 | 9.9 | 4.0 | 7.0 | 66.0 | 104 |
| 69 |  | 35 | 50 | 27 | 2.69 | 16.18 | 20 | 5.8 | 2.3 | 12.0 | 53.0 | 61.3 |
| 70 | [24, 25] | 65 | 86 | 49 | 2.64 | 12.65 | 36.5 | 9.8 | 30.8 | 18.3 | 16.0 | 80 |
| 71 | [26] | 22.5 | 51.9 | 24 | 2.71 | 18.02 | 15.6 | 5.44 | 2.2 | 10.3 | 64.1 | 103 |
| 72 | [27] | 63 | 77 | 49 | 2.72 | 14.51 | 26.5 | 32.7 | 13.0 | 5.0 | 33.0 | 250 |
| 73 |  | 34 | 54 | 31 | 2.76 | 15.08 | 20 | 8.5 | 3.4 | 21.8 | 36.3 | 123 |
| 74 |  | 53 | 76 | 52 | 2.64 | 14.64 | 26 | 18.5 | 7.4 | 4.7 | 36.0 | 210 |
| 75 | [28] | 62 | 63 | 45 | 2.74 | 13 | 17.45 | 5.5 | 2.2 | 8.0 | 30.0 | 124 |
| 76 | [29] | 17 | 50 | 25 | 2.71 | 14.62 | 22 | 3.2 | 1.3 | 14.0 | 75.0 | 42 |
| 77 |  | 22 | 39 | 12 | 2.69 | 16 | 23.4 | 0.2 | 22.5 | 5.0 | 66.0 | 18.4 |
| 78 |  | 37 | 70 | 34 | 2.69 | 12.1 | 28.2 | 0.5 | 25.3 | 1.0 | 51.0 | 12.5 |
| 79 |  | 48 | 62 | 31 | 2.67 | 13.1 | 26.05 | 1.8 | 27.4 | 3.0 | 32.0 | 52.1 |
| 80 | [30] | 51 | 59 | 27 | 2.8 | 15.9 | 26.2 | 2.3 | 0.9 | 4.0 | 33.0 | 75 |
| 81 | [31] | 45 | 57 | 28 | 2.67 | 17.1 | 17.6 | 4.32 | 1.7 | 0.0 | 55.0 | 230 |
| 82 | [32] | 56 | 61 | 31 | 2.69 | 14.21 | 25.38 | 9.5 | 3.8 | 25.0 | 19.0 | 90 |
| 83 |  | 70 | 72 | 40 | 2.76 | 15.22 | 23.4 | 15 | 6.0 | 10.0 | 20.0 | 130 |
| 84 |  | 52 | 54 | 29 | 2.72 | 13.8 | 25.6 | 7 | 2.8 | 28.0 | 20.0 | 95 |
| 85 |  | 62 | 70 | 42 | 2.7 | 14.33 | 24 | 10 | 4.0 | 20.0 | 18.0 | 122 |
| 86 |  | 14 | 37 | 10 | 2.66 | 14.5 | 22.7 | 1.6 | 0.6 | 64.0 | 22.0 | 28 |
| 87 |  | 21 | 45 | 17 | 2.7 | 13.2 | 26.7 | 4 | 1.6 | 61.0 | 18.0 | 50 |
| 88 | [33] | 26 | 58 | 28 | 2.6 | 15 | 17 | 13 | 5.2 | 11.0 | 74.0 | 90 |
| 89 |  | 30 | 85 | 48 | 2.65 | 13.9 | 21 | 17 | 6.8 | 5.4 | 70.0 | 102 |
| 90 |  | 35 | 130 | 82 | 2.69 | 13.5 | 23 | 28 | 11.2 | 2.1 | 65.0 | 160 |
| 91 | [34] | 50 | 52 | 32 | 2.59 | 16.5 | 24.6 | 4.4 | 1.8 | 27.0 | 22.0 | 77 |
| 92 | [35] | 43 | 62.7 | 32.3 | 2.29 | 15 | 22.8 | 4 | 1.6 | 12.0 | 28.0 | 78 |
| 93 | [36] | 35.03 | 50.3 | 26.5 | 2.65 | 15.6 | 21.1 | 3.4 | 1.4 | 2.1 | 62.8 | 43.5 |
| 94 |  | 14.69 | 45.8 | 20.5 | 2.75 | 16 | 20.8 | 1.85 | 0.7 | 3.1 | 81.2 | 32 |
| 95 | [37] | 46 | 57 | 38 | 2.66 | 14.5 | 22.7 | 4.7 | 1.9 | 3.0 | 34.0 | 55 |
| 96 | [38] | 30 | 43 | 19 | 2.6 | 15.65 | 21.5 | 2.58 | 1.0 | 22.0 | 27.5 | 70 |
| 97 | [39] | 65.2 | 52.2 | 47.97 | 2.7 | 14.22 | 31.7 | 5.04 | 31.7 | 2.0 | 33.0 | 100 |
| 98 | [40] | 60 | 79 | 53 | 2.73 | 12 | 22.7 | 21.78 | 8.7 | 14.0 | 26.0 | 152 |
| 99 | [41] | 40 | 76 | 58 | 2.84 | 13.47 | 31 | 7.1 | 2.8 | 18.0 | 26.0 | 93 |
| 100 | [42] | 32.2 | 48 | 27 | 2.72 | 17.15 | 18 | 3.46 | 1.4 | 3.0 | 32.0 | 120 |
| 101 |  | 48.6 | 65 | 42 | 2.71 | 16.48 | 19 | 5.32 | 2.1 | 11.0 | 34.0 | 230 |
| 102 | [43] | 40 | 64 | 37 | 2.75 | 17.3 | 18 | 14.5 | 5.8 | 18.0 | 40.0 | 135 |
| 103 | [44] | 20 | 56.8 | 21.9 | 2.7 | 17.5 | 21 | 8.36 | 8.9 | 14.0 | 40.0 | 182 |
| 104 | [45] | 58 | 59 | 31 | 2.68 | 15.89 | 21 | 5.9 | 30.9 | 12.0 | 30.0 | 202 |
| 105 |  | 54 | 88 | 53 | 2.72 | 16.19 | 21 | 6.75 | 30.2 | 22.0 | 24.0 | 260 |
| 106 | [46] | 35.2 | 65 | 30 | 2.7 | 13.7 | 29.1 | 2.6 | 29.1 | 23.2 | 36.3 | 19.8 |
| 107 |  | 35.1 | 68 | 35 | 2.7 | 12.9 | 23.1 | 7.7 | 23.1 | 21.8 | 36.3 | 51.1 |
| 108 |  | 35.5 | 67 | 34 | 2.7 | 12.6 | 32.7 | 5.3 | 32.7 | 22.2 | 34.2 | 57.4 |
| 109 |  | 23.2 | 51 | 23 | 2.7 | 16.7 | 17 | 4.7 | 17.0 | 39.3 | 26.7 | 71.6 |
| 110 |  | 21.8 | 50 | 23 | 2.7 | 14.9 | 23.2 | 2.6 | 23.2 | 38.5 | 31.8 | 18.8 |
| 111 |  | 26.5 | 54 | 26 | 2.7 | 15.9 | 19.6 | 5.5 | 19.6 | 34.3 | 31.8 | 72.2 |
| 112 | [47] | 33.2 | 115 | 70.57 | 2.63 | 14.51 | 27.92 | 8.35 | 27.9 | 8.0 | 12.7 | 133 |
| 113 |  | 33.1 | 100 | 64.35 | 2.63 | 12.55 | 29.76 | 6.09 | 35.9 | 5.4 | 10.0 | 83.4 |
| 114 | [48] | 50 | 60.2 | 28.2 | 2.67 | 15 | 22.8 | 4.3 | 1.7 | 26.7 | 23.2 | 124 |
| 115 | [49] | 93.3 | 74 | 47 | 2.71 | 16.2 | 16.8 | 11.5 | 4.6 | 3.6 | 3.1 | 219 |
| 116 | [50] | 33 | 71 | 39 | 2.77 | 15.6 | 22.5 | 7 | 2.8 | 5.4 | 55.3 | 120 |
| 117 | [51] | 27.5 | 35 | 13.8 | 2.69 | 18.62 | 16.5 | 3 | 1.2 | 7.0 | 66.0 | 120 |
| 118 |  | 43 | 56.5 | 28 | 2.73 | 19.32 | 23.2 | 13.2 | 5.3 | 15.0 | 29.0 | 131 |
| 119 |  | 35 | 72 | 30.5 | 2.73 | 19.26 | 16.5 | 1.8 | 0.7 | 18.0 | 26.0 | 165 |
| 120 |  | 47.5 | 80 | 47.5 | 2.74 | 20.75 | 17.5 | 2.5 | 1.0 | 28.0 | 20.0 | 182 |
| 121 |  | 59 | 54 | 32 | 2.74 | 19.47 | 19.5 | 2.2 | 0.9 | 7.0 | 33.0 | 169 |
| 122 | [52] | 40 | 52 | 30 | 2.61 | 14.8 | 13 | 30 | 13.0 | 5.0 | 55.0 | 258 |
| 123 | [53] | 35 | 42 | 26 | 2.64 | 16.3 | 18.6 | 1.6 | 19.4 | 18.0 | 72.0 | 96 |
| 124 | [54] | 30 | 65 | 41 | 2.59 | 12.72 | 34.4 | 11 | 4.4 | 24.0 | 46.0 | 65 |
| 125 |  | 53 | 71 | 48 | 2.59 | 11.95 | 36.9 | 16.4 | 6.5 | 28.0 | 19.0 | 84.4 |
| 126 |  | 73 | 96 | 67 | 2.59 | 11.1 | 31 | 22 | 8.8 | 21.0 | 6.0 | 137 |
| 127 | [55] | 55.3 | 45 | 22 | 2.55 | 15.25 | 20 | 4.6 | 1.8 | 16.0 | 17.6 | 67 |
| 128 |  | 61.2 | 133 | 83 | 2.61 | 11.5 | 20 | 15.9 | 6.3 | 20.0 | 18.8 | 148 |
| 129 | [56] | 23 | 53 | 24 | 2.69 | 15.1 | 16.5 | 4.3 | 16.5 | 2.0 | 75.0 | 64 |
| 130 |  | 23 | 51 | 28 | 2.69 | 16.1 | 23.2 | 6 | 23.2 | 2.0 | 75.0 | 95 |
| 131 |  | 23 | 58 | 28 | 2.69 | 15.4 | 17.2 | 5.4 | 17.2 | 2.0 | 75.0 | 86 |
| 132 |  | 23 | 51 | 25 | 2.69 | 16.2 | 16.5 | 5.7 | 16.5 | 2.0 | 75.0 | 102 |
| 133 |  | 24 | 51 | 26 | 2.67 | 16.3 | 17.5 | 6.9 | 17.5 | 2.0 | 74.0 | 107 |
| 134 |  | 24 | 50 | 26 | 2.67 | 17.9 | 19.5 | 7.6 | 19.5 | 2.0 | 74.0 | 134 |
| 135 |  | 24 | 54 | 27 | 2.67 | 16.2 | 14.5 | 6.5 | 14.5 | 2.0 | 74.0 | 95 |
| 136 |  | 20 | 42 | 22 | 2.67 | 16.5 | 18 | 6.6 | 18.0 | 2.0 | 74.0 | 93 |
| 137 |  | 20 | 51 | 24 | 2.67 | 14.8 | 16 | 4.2 | 16.0 | 2.0 | 74.0 | 43 |
| 138 |  | 20 | 51 | 28 | 2.67 | 16.1 | 20.5 | 6.8 | 20.5 | 2.0 | 74.0 | 102 |
| 139 |  | 20 | 56 | 28 | 2.67 | 14.9 | 10.5 | 5.4 | 10.5 | 2.0 | 74.0 | 68 |
| 140 |  | 20 | 54 | 26 | 2.67 | 16.3 | 17.5 | 6.7 | 17.5 | 2.0 | 74.0 | 105 |
| 141 |  | 22 | 51 | 23 | 2.67 | 16 | 18.3 | 5.2 | 18.3 | 2.0 | 74.0 | 90 |
| 142 |  | 21 | 42 | 21 | 2.65 | 16.8 | 11.4 | 5.8 | 11.4 | 1.0 | 78.0 | 98 |
| 143 |  | 22 | 50 | 25 | 2.65 | 15.5 | 17.9 | 5.8 | 17.9 | 1.0 | 78.0 | 76 |
| 144 |  | 22 | 42 | 21 | 2.65 | 16.5 | 18.6 | 5.4 | 18.6 | 1.0 | 78.0 | 86 |
| 145 |  | 22 | 58 | 28 | 2.65 | 16.4 | 15 | 7.5 | 15.0 | 1.0 | 78.0 | 100 |
| 146 |  | 22 | 55 | 27 | 2.65 | 14.5 | 18.2 | 3.9 | 18.2 | 1.0 | 78.0 | 62 |
| 147 |  | 22 | 52 | 25 | 2.65 | 14.6 | 15 | 4.9 | 15.0 | 1.0 | 78.0 | 55 |
| 148 |  | 22 | 56 | 28 | 2.65 | 16.1 | 18 | 6.6 | 18.0 | 1.0 | 78.0 | 105 |
| 149 |  | 21 | 52 | 26 | 2.65 | 16.2 | 22.7 | 6.7 | 22.7 | 1.0 | 78.0 | 91 |
| 150 |  | 25 | 55 | 28 | 2.7 | 14.9 | 10.1 | 4.7 | 10.1 | 1.0 | 74.0 | 71 |
| 151 |  | 21 | 46 | 23 | 2.7 | 16.2 | 18 | 5.5 | 18.0 | 1.0 | 74.0 | 83 |
| 152 |  | 21 | 37 | 21 | 2.7 | 16.8 | 22.3 | 6.7 | 22.3 | 1.0 | 74.0 | 94 |
| 153 |  | 21 | 50 | 21 | 2.7 | 16.8 | 11.5 | 6.5 | 11.5 | 1.0 | 74.0 | 105 |
| 154 |  | 42 | 50 | 25 | 2.7 | 15.6 | 25.3 | 5 | 25.3 | 1.0 | 74.0 | 81 |
| 155 |  | 42 | 50 | 23 | 2.7 | 15.2 | 22.1 | 4.2 | 22.1 | 1.0 | 74.0 | 67 |
| 156 |  | 42 | 51 | 24 | 2.7 | 15.1 | 16.5 | 5.1 | 16.5 | 1.0 | 74.0 | 60 |
| 157 | [57] | 23 | 55 | 30 | 2.65 | 18.3 | 15 | 7.6 | 11.0 | 11.0 | 74.0 | 154 |
| 158 |  | 22 | 40 | 20 | 2.62 | 17.9 | 13.5 | 5 | 9.7 | 11.0 | 74.0 | 128 |
| 159 | [58] | 22 | 55 | 37 | 2.69 | 16.33 | 19 | 8.8 | 3.5 | 34.3 | 31.8 | 183 |
| 160 |  | 25 | 63 | 42 | 2.69 | 16.49 | 19 | 12 | 4.8 | 7.0 | 66.0 | 194 |
| 161 |  | 20 | 46 | 26 | 2.66 | 16.93 | 19 | 6.2 | 2.5 | 11.0 | 65.0 | 88.3 |
| 162 |  | 11 | 24 | 12 | 2.61 | 14.62 | 27 | 9.1 | 3.6 | 13.0 | 75.0 | 158 |
| 163 | [59] | 68 | 73.3 | 54.3 | 2.6 | 16.97 | 16.4 | 7.81 | 16.4 | 4.0 | 24.5 | 271 |
| 164 |  | 64 | 74.8 | 41.5 | 2.64 | 16.19 | 22.09 | 6.6 | 22.1 | 4.2 | 32.7 | 258 |
| 165 |  | 66 | 71.7 | 52.68 | 2.6 | 15.99 | 19.39 | 6.57 | 19.4 | 2.0 | 31.0 | 236 |
| 166 | [60] | 60 | 56 | 31 | 2.74 | 18.3 | 14 | 6 | 2.4 | 3.0 | 37.0 | 180 |
| 167 | [61] | 71 | 72.3 | 43.08 | 2.74 | 17.5 | 14.1 | 5.4 | 14.1 | 4.5 | 24.5 | 190 |
| 168 | [62] | 60.4 | 63 | 38.06 | 2.7 | 18 | 16.2 | 19 | 7.6 | 3.9 | 35.7 | 183 |

|  | Unconfined compression strength, UCS | | | | | | | | | | | |
| --- | --- | --- | --- | --- | --- | --- | --- | --- | --- | --- | --- | --- |
| **S. No** | **Reference** | **Clay fraction**  **(CF)** | **Liquid**  **Limit**  **(LL)** | **Plasticity**  **Index**  **(PI)** | **Specific**  **Gravity**  **(G_s_)** | **Max. Dry**  **Density**  **(MDD)** | **Optimum**  **Moisture**  **Content**  **(OMC)** | **Swell**  **Potential**  **(SP)** | **Optimum**  **Moisture**  **Content**  **(w_n_)** | **Sand** | **Silt** | **Unconfined**  **Compression**  **Strength**  **(UCS)** |
|  |  | % | % | % |  | kN/m^3^ | % | % | % | % | % | KPa |
| 1 | [63] | 47 | 44.2 | 19.39 | 2.67 | 17.06 | 18.5 | 5.28 | 2.1 | 19.0 | 34.0 | 205 |
| 2 | [64] | 70 | 78 | 33 | 2.66 | 12.99 | 35 | 7.25 | 2.9 | 1.0 | 29.0 | 250 |
| 3 | [65] | 78 | 412 | 352 | 2.71 | 12.6 | 41 | 22 | 8.8 | 0.0 | 22.0 | 390 |
| 4 |  | 74 | 355 | 307 | 2.71 | 13.2 | 26 | 17.5 | 7.0 | 0.0 | 26.0 | 340 |
| 5 |  | 72 | 255 | 215 | 2.7 | 14 | 24 | 15.2 | 6.1 | 0.0 | 28.0 | 258 |
| 6 |  | 65 | 148 | 116 | 2.68 | 14.9 | 23 | 9.3 | 3.7 | 0.0 | 35.0 | 242 |
| 7 | [66] | 38 | 47 | 26 | 2.57 | 16.38 | 18 | 2.2 | 0.9 | 21 | 18 | 190 |
| 8 | [67] | 25 | 47 | 35 | 2.69 | 14.2 | 24 | 3 | 1.2 | 3.0 | 40.0 | 202 |
| 9 |  | 55 | 82 | 55 | 2.69 | 13.8 | 28 | 4 | 1.6 | 4.0 | 29.0 | 225 |
| 10 | [68] | 94 | 75 | 33 | 2.57 | 13.73 | 29.5 | 16.85 | 6.7 | 2.0 | 4.0 | 350 |
| 11 |  | 76 | 114 | 47 | 2.63 | 12.65 | 20 | 17.18 | 6.9 | 4.0 | 20.0 | 127 |
| 12 |  | 68 | 52 | 14 | 2.65 | 13.83 | 28.5 | 14.21 | 5.7 | 3.0 | 29.0 | 318 |
| 13 |  | 76 | 58 | 32 | 2.6 | 16.81 | 22 | 15.92 | 26.0 | 10.0 | 43.0 | 178 |
| 14 | [69] | 15 | 32.8 | 19.9 | 2.61 | 15.98 | 21.2 | 11.82 | 4.7 | 26.0 | 59.0 | 108 |
| 15 | [5] | 26 | 58.5 | 33 | 2.7 | 16.5 | 19 | 12.7 | 19.0 | 4.0 | 57.0 | 330 |
| 16 |  | 58 | 63 | 35.5 | 2.7 | 14.5 | 26 | 11.3 | 26.0 | 5.0 | 7.0 | 130 |
| 17 |  | 32 | 59.5 | 30.5 | 2.7 | 14.5 | 30 | 11.82 | 30.0 | 2.1 | 62.8 | 126 |
| 18 |  | 41 | 64 | 35.5 | 2.7 | 15 | 28.5 | 14.49 | 28.5 | 12 | 45 | 62 |
| 19 |  | 19 | 43 | 21.5 | 2.68 | 14 | 25 | 13.4 | 25.0 | 14 | 59.4 | 46 |
| 20 |  | 43 | 57.5 | 28.5 | 2.69 | 15.5 | 26.5 | 15.05 | 26.5 | 3.0 | 41.0 | 60 |
| 21 | [6] | 69 | 80 | 52 | 2.72 | 13.75 | 40 | 10.8 | 14.0 | 7.0 | 24.0 | 78.25 |
| 22 | [70] | 50 | 60 | 38 | 2.37 | 12.4 | 31 | 14.42 | 5.8 | 13.0 | 41.0 | 210 |
| 23 |  | 30 | 30 | 10 | 2.52 | 16.3 | 16.3 | 12.42 | 5.0 | 21.0 | 62.0 | 190 |
| 24 | [71] | 36 | 68 | 38 | 2.7 | 12.36 | 14 | 14.6 | 5.8 | 21 | 18 | 76 |
| 25 | [72] | 65 | 93 | 72 | 2.5 | 13.83 | 24.3 | 19 | 15.0 | 3.0 | 22.0 | 319 |
| 26 | [47, 73, 74] | 33 | 43.5 | 18.6 | 2.69 | 17.55 | 14.6 | 4.65 | 1.9 | 22.5 | 41.5 | 385 |
| 27 | [15] | 55 | 95 | 70 | 2.65 | 12.94 | 32 | 16.6 | 6.6 | 4.0 | 29.0 | 300 |
| 28 | [75] | 56 | 75 | 50 | 2.7 | 14.21 | 25 | 4.95 | 2.0 | 16.0 | 28.0 | 127 |
| 29 | [76] | 59 | 95 | 71 | 2.77 | 14.12 | 28 | 3.6 | 1.4 | 23.0 | 18.0 | 136 |
| 30 | [77] | 76 | 85 | 46 | 2.68 | 13.92 | 26.89 | 16.71 | 6.7 | 2.0 | 22.0 | 92 |
| 31 | [78] | 42 | 70.7 | 39.7 | 2.69 | 15.22 | 16 | 40 | 3.4 | 13.0 | 48.0 | 591.8 |
| 32 | [79] | 25.7 | 83.7 | 50.9 | 2.6 | 15.69 | 19.43 | 32.7 | 13.3 | 7.0 | 69.8 | 115 |
| 33 | [80] | 60 | 140 | 75 | 2.7 | 11.7 | 36 | 17 | 6.8 | 12.0 | 30.0 | 165 |
| 34 | [66] | 38 | 47 | 26 | 2.57 | 16.38 | 18 | 2.2 | 0.9 | 12 | 32 | 195 |
| 35 | [23] | 69 | 190 | 145 | 2.6 | 16.18 | 4 | 23.6 | 9.4 | 3.0 | 28.0 | 180.8 |
| 36 |  | 19 | 85 | 41 | 2.7 | 16.18 | 8 | 7.5 | 3.0 | 2.0 | 79.0 | 120.5 |
| 37 |  | 27 | 100 | 50 | 2.69 | 16.18 | 16 | 9.9 | 4.0 | 7.0 | 66.0 | 125 |
| 38 | [81] | 58 | 78 | 49 | 2.67 | 14.8 | 28.5 | 6.2 | 2.5 | 4.0 | 21.0 | 240 |
| 39 | [82] | 58 | 85 | 38.09 | 2.26 | 14.51 | 14.8 | 17 | 26.1 | 7.0 | 30.0 | 85 |
| 40 | [83] | 34.9 | 41 | 17 | 2.62 | 18.97 | 14.7 | 15.01 | 5.1 | 4.0 | 16.0 | 280 |
| 41 | [84] | 54 | 60.08 | 35.53 | 2.64 | 14.26 | 25.32 | 14.8 | 5.9 | 8.0 | 38.0 | 78.38 |
| 42 | [24, 25] | 65 | 86 | 49 | 2.64 | 12.65 | 36.5 | 9.8 | 30.8 | 18.3 | 16.0 | 149 |
| 43 | [85] | 43 | 53 | 31 | 2.69 | 18.14 | 14.3 | 14.6 | 5.8 | 18.0 | 11.0 | 598 |
| 44 | [26] | 22.5 | 51.9 | 24 | 2.71 | 18.02 | 15.6 | 5.44 | 2.2 | 10.3 | 64.1 | 382 |
| 45 | [27] | 63 | 77 | 49 | 2.72 | 14.51 | 26.5 | 32.7 | 13.0 | 5.0 | 30.0 | 370 |
| 46 |  | 53 | 76 | 52 | 2.64 | 14.64 | 26 | 18.5 | 7.4 | 7.0 | 37.0 | 294 |
| 47 | [86] | 20 | 58 | 34 | 2.71 | 14.94 | 12 | 13.52 | 5.4 | 4 | 50.0 | 125 |
| 48 |  | 18 | 30 | 13 | 2.73 | 17.33 | 18 | 11.8 | 4.7 | 2.0 | 40.0 | 112 |
| 49 | [87] | 35 | 68 | 18.35 | 2.72 | 15.89 | 29.4 | 15.64 | 6.2 | 21 | 18 | 160 |
| 50 | [88] | 60 | 84.8 | 52.02 | 2.71 | 13.8 | 28.3 | 16.02 | 32.9 | 3.0 | 31.0 | 34.6 |
| 51 |  | 65 | 47.79 | 24.56 | 2.84 | 16.9 | 15.27 | 15.5 | 13.8 | 21.0 | 16.0 | 113.2 |
| 52 | [89] | 82 | 66 | 36 | 2.7 | 14 | 27.5 | 16.4 | 6.5 | 1.0 | 17.0 | 222 |
| 53 | [90] | 57 | 55.2 | 17.1 | 2.7 | 14.22 | 24.5 | 15.1 | 4.9 | 6.0 | 31.0 | 125 |
| 54 |  | 52 | 53.6 | 16.3 | 2.65 | 14.27 | 24.2 | 14.9 | 3.1 | 5.0 | 35.0 | 240 |
| 55 | [91] | 15 | 42.8 | 20.8 | 2.71 | 17.26 | 18.9 | 12.1 | 4.8 | 14 | 59.4 | 300 |
| 56 | [92] | 21 | 59 | 32 | 2.67 | 14.91 | 23 | 12.8 | 5.1 | 29.0 | 50.0 | 16.8 |
| 57 | [93] | 42 | 52 | 32 | 2.61 | 16.5 | 24 | 14 | 5.6 | 12 | 45 | 180 |
| 58 | [94] | 43 | 330 | 287 | 2.6 | 12.16 | 40 | 38.4 | 15.3 | 12.0 | 21.0 | 525 |
| 59 | [95] | 31 | 42.6 | 16.2 | 2.81 | 16.6 | 17 | 8.5 | 3.4 | 2.1 | 62.8 | 200 |
| 60 | [96] | 70 | 62 | 39 | 2.55 | 16.28 | 17.5 | 16.5 | 6.6 | 5.0 | 25.0 | 240 |
| 61 | [97] | 10 | 48 | 30 | 2.7 | 15.89 | 22.9 | 12.5 | 5.0 | 14 | 64.7 | 105 |
| 62 | [98] | 75 | 123 | 81 | 2.84 | 12.75 | 34 | 16 | 25.0 | 2.5 | 22.5 | 278.5 |
| 63 | [99] | 70.84 | 67.1 | 43.6 | 2.66 | 15.16 | 23.4 | 17.4 | 8.7 | 4.2 | 25.0 | 152.5 |
| 64 | [100] | 76 | 49 | 19.82 | 2.38 | 15.89 | 20.83 | 15.5 | 6.2 | 11.0 | 13.0 | 218.4 |
| 65 | [101] | 21 | 61.9 | 39.6 | 2.67 | 15.89 | 22.2 | 7.95 | 3.2 | 9.0 | 70.0 | 234 |
| 66 | [102] | 76 | 63 | 38 | 2.58 | 16.6 | 24.6 | 16.4 | 6.5 | 11.0 | 13.0 | 188 |
| 67 | [34] | 50 | 52 | 32 | 2.59 | 16.5 | 24.6 | 14.4 | 5.7 | 27.0 | 22.0 | 185 |
| 68 | [103] | 56.1 | 36.32 | 15.02 | 2.67 | 16.57 | 16.8 | 15.2 | 15.8 | 7.0 | 14.8 | 134 |
| 69 | [36] | 35.03 | 50.3 | 26.5 | 2.65 | 15.6 | 21.1 | 13.4 | 5.3 | 2.1 | 62.8 | 285.6 |
| 70 | [104] | 8.5 | 46.1 | 16.7 | 2.47 | 12.85 | 13.8 | 11.7 | 4.7 | 8.1 | 83.5 | 72.98 |
| 71 | [38] | 70 | 111 | 71 | 2.53 | 11.04 | 32.6 | 17.9 | 7.1 | 5.0 | 25.0 | 155.1 |
| 72 |  | 40 | 62 | 41 | 2.66 | 13.98 | 28.5 | 9.14 | 3.6 | 21 | 18 | 88.2 |
| 73 |  | 30 | 43 | 19 | 2.6 | 15.65 | 21.5 | 2.58 | 1.0 | 12 | 56 | 69.6 |
| 74 | [105] | 31 | 84.3 | 34.8 | 2.65 | 14.22 | 24.9 | 13.45 | 5.4 | 22.2 | 46 | 85 |
| 75 | [39] | 65.2 | 52.18 | 47.97 | 2.7 | 14.22 | 31.7 | 5.04 | 31.7 | 2 | 32 | 103.9 |
| 76 | [41] | 40 | 76 | 58 | 2.84 | 13.47 | 31 | 17.1 | 6.8 | 12 | 32 | 117.2 |
| 77 | [106] | 59 | 62.5 | 31.8 | 2.58 | 15.4 | 20.54 | 15.23 | 6.1 | 6 | 8 | 127.5 |
| 78 | [107] | 43 | 65 | 29 | 2.69 | 16.9 | 18 | 5.8 | 2.3 | 7 | 50 | 280 |
| 79 | [108] | 36 | 63 | 34 | 2.58 | 14.53 | 26 | 11.8 | 4.7 | 6 | 54 | 202 |
| 80 | [109] | 60 | 69 | 38 | 2.66 | 14.32 | 24 | 16.2 | 30.0 | 7 | 33 | 175 |
| 81 | [110] | 40 | 51 | 24 | 2.72 | 16.6 | 19.7 | 5.76 | 19.7 | 7 | 53 | 20 |
| 82 | [111] | 39 | 37 | 11 | 2.61 | 16 | 23 | 12.6 | 5.0 | 3 | 60 | 200 |
| 83 | [112] | 50 | 62 | 38 | 2.59 | 16.5 | 14.6 | 15.40 | 6.1 | 28 | 28 | 520 |
| 84 |  | 41 | 54 | 31 | 2.59 | 16.8 | 14.5 | 14.45 | 5.8 | 41 | 21 | 580 |
| 85 |  | 51 | 54 | 30 | 2.59 | 17.9 | 13.6 | 15.04 | 6.0 | 20 | 20 | 620 |
| 86 |  | 51 | 43 | 18 | 2.59 | 18.55 | 11.9 | 14.43 | 5.8 | 29 | 22 | 780 |
| 87 |  | 52 | 38 | 15 | 2.59 | 19.1 | 10.7 | 14.38 | 5.7 | 30 | 41 | 940 |
| 88 |  | 29 | 48 | 27 | 2.59 | 19.3 | 10.5 | 13.73 | 5.5 | 49 | 13 | 960 |
| 89 |  | 40 | 42 | 20 | 2.59 | 19.4 | 11 | 13.94 | 5.6 | 42 | 23 | 1010 |
| 90 |  | 41 | 42 | 20 | 2.59 | 19.4 | 11.6 | 13.96 | 5.6 | 40 | 17 | 1020 |
| 91 |  | 35 | 44 | 22 | 2.59 | 19.45 | 10.6 | 13.78 | 5.5 | 36 | 14 | 1030 |
| 92 |  | 37 | 36 | 14 | 2.59 | 19.5 | 10.4 | 13.44 | 5.4 | 37 | 50 | 1060 |
| 93 |  | 40 | 35 | 14 | 2.59 | 19.9 | 10 | 13.64 | 5.4 | 46 | 15 | 1280 |
| 94 | [113] | 65.93 | 150 | 114 | 1.17 | 5.8 | 73 | 17.42 | 450.0 | 2 | 32 | 28.56 |
| 95 |  | 65 | 78 | 48.97 | 1.45 | 7.1 | 61 | 14.18 | 450.0 | 16.0 | 16.0 | 31.28 |
| 96 |  | 63.45 | 79 | 49.86 | 1.62 | 7.3 | 62 | 14.08 | 450.0 | 4.8 | 31.7 | 34.5 |
| 97 |  | 62.5 | 75 | 46.3 | 1.78 | 7.5 | 65 | 13.63 | 450.0 | 3 | 31 | 40.38 |
| 98 |  | 61.4 | 73 | 44.5 | 1.64 | 8.1 | 57 | 13.93 | 450.0 | 2 | 30 | 38.3 |
| 99 |  | 64.25 | 75 | 45 | 1.48 | 7.1 | 59 | 14.02 | 450.0 | 4 | 32 | 36.96 |
| 100 |  | 63.55 | 77 | 48.1 | 1.56 | 7.3 | 62 | 13.98 | 450.0 | 7 | 32 | 29.74 |
| 101 |  | 31.98 | 69 | 40.96 | 1.82 | 8.5 | 60 | 11.78 | 250.0 | 22.5 | 41.5 | 43.28 |
| 102 | [114] | 45 | 53 | 27 | 2.69 | 17 | 21.5 | 14.03 | 21.5 | 4 | 51 | 123 |
| 103 | [115] | 36 | 91 | 51 | 2.7 | 13.1 | 37 | 6 | 8.0 | 9 | 53.6 | 270 |
| 104 | [116] | 57.4 | 46.5 | 17.82 | 2.72 | 17.9 | 15.25 | 5.5 | 2.2 | 1.5 | 40.8 | 240 |
| 105 | [117] | 10 | 26.1 | 54.4 | 2.7 | 15.52 | 23 | 13.48 | 5.4 | 12 | 70 | 6.43 |
| 106 | [118] | 11 | 34 | 14 | 2.69 | 17.89 | 14.78 | 11.61 | 36.8 | 2.0 | 17 | 186 |
| 107 | [74] | 47.2 | 52.2 | 28.1 | 2.692 | 18.38 | 17.4 | 3.71 | 1.5 | 3.6 | 49.2 | 425 |
| 108 | [119] | 63.5 | 82.8 | 50.6 | 2.71 | 13.8 | 28.3 | 17.2 | 32.9 | 3 | 21.5 | 100 |
| 109 | [120] | 15.4 | 47 | 21.85 | 2.67 | 18.04 | 13 | 12.7 | 10.0 | 2.0 | 10 | 188.4 |
| 110 | [121] | 63 | 82 | 52 | 2.68 | 14.61 | 27.7 | 16.3 | 6.5 | 0 | 37 | 200.8 |
| 111 | [122] | 57 | 64 | 39 | 2.6 | 15.04 | 25.06 | 15.3 | 6.1 | 4 | 32 | 240 |
| 112 | [123] | 25 | 33.3 | 16.3 | 2.67 | 19.22 | 11.1 | 2.7 | 11.1 | 2 | 55.9 | 317.7 |
| 113 | [124] | 13 | 43 | 15 | 2.55 | 16.97 | 15.7 | 12.2 | 8.6 | 2.0 | 79.0 | 212.2 |
| 114 | [125] | 65 | 88 | 55 | 2.61 | 14 | 27 | 16.6 | 6.6 | 11 | 24 | 220 |
| 115 | [126] | 38.4 | 77.75 | 43.38 | 2.33 | 14.67 | 20.5 | 14.68 | 5.9 | 18.1 | 43.5 | 210.6 |
| 116 | [127] | 61 | 91 | 66 | 2.82 | 13.7 | 29 | 17.5 | 7.0 | 25.4 | 13.6 | 200 |
| 117 | [53] | 35 | 42 | 26 | 2.64 | 16.3 | 18.6 | 1.6 | 19.4 | 4 | 72 | 208 |
| 118 | [128] | 32 | 60 | 29 | 2.6 | 12.65 | 32 | 12.8 | 5.1 | 4 | 32 | 110 |
| 119 | [57] | 23 | 55 | 30 | 2.65 | 18.3 | 15 | 7.6 | 11.0 | 5 | 54 | 205 |
| 120 |  | 22 | 40 | 20 | 2.62 | 17.9 | 13.5 | 5 | 9.7 | 10.3 | 64.1 | 215 |
| 121 | [129] | 53 | 75 | 46 | 2.69 | 15.98 | 21.5 | 15.7 | 6.3 | 6 | 36 | 135 |
| 122 | [130] | 9 | 29.6 | 10.11 | 2.64 | 18.05 | 15.75 | 4 | 2.2 | 18 | 67 | 122.6 |
| 123 |  | 11 | 33.35 | 7.96 | 2.66 | 17.76 | 16 | 3 | 1.6 | 26.0 | 17 | 171.6 |
| 124 |  | 13 | 40.5 | 10.73 | 2.63 | 16.97 | 15.95 | 3 | 2.3 | 2.0 | 40.0 | 289.3 |
| 125 | [131] | 24 | 36.6 | 16.94 | 2.7 | 17.76 | 22.48 | 11.1 | 42.3 | 2 | 74 | 40 |
| 126 |  | 40 | 45.5 | 21.35 | 2.72 | 18.05 | 26.21 | 12.4 | 41.9 | 1 | 59 | 38.66 |
| 127 | [132] | 70 | 116.8 | 81.54 | 2.6 | 16.92 | 18 | 19 | 7.6 | 5.0 | 25.0 | 150 |
| 128 | [133] | 71.5 | 87 | 38 | 2.78 | 17.65 | 19.2 | 16.42 | 6.6 | 4 | 24.5 | 145 |
| 129 |  | 63.5 | 69.7 | 37.03 | 2.68 | 17.79 | 18.62 | 15.9 | 6.3 | 4.8 | 31.7 | 290 |
| 130 |  | 57.2 | 73.51 | 32.14 | 2.63 | 18.02 | 17.93 | 15.28 | 6.1 | 5.4 | 37.4 | 185 |
| 131 | [134] | 5 | 50.82 | 14.86 | 2.62 | 15.43 | 20.5 | 11 | 4.4 | 35 | 60 | 104.6 |
| 132 | [135] | 1 | 28 | 4.52 | 2.66 | 17.5 | 16 | 4 | 25.0 | 5 | 94 | 33.25 |
| 133 | [136] | 26 | 58 | 28 | 2.6 | 15 | 17 | 12.6 | 5.0 | 5 | 74 | 198 |
| 134 | [137] | 43 | 52 | 28 | 2.72 | 16.97 | 17 | 14.3 | 5.7 | 12 | 45 | 200 |
| 135 | [138] | 20 | 56 | 39 | 2.3 | 16 | 18.2 | 13.5 | 5.4 | 14 | 59.4 | 26 |
| 136 | [60] | 60 | 56 | 31 | 2.74 | 18.3 | 14 | 6 | 2.4 | 3 | 37 | 322 |
| 137 | [139] | 8 | 29 | 8 | 2.67 | 13.93 | 19.28 | 11.8 | 3.6 | 16 | 71 | 300 |
| 138 | [140] | 74 | 68.5 | 5.13 | 2.66 | 11.58 | 38.18 | 13.5 | 5.4 | 0 | 26 | 142 |
| 139 |  | 66 | 94 | 4.7 | 2.8 | 12.59 | 34.8 | 13.1 | 5.2 | 2 | 32 | 115 |
| 140 | [141] | 10 | 42.6 | 20.1 | 2.72 | 17.45 | 20.2 | 11.6 | 4.6 | 16 | 64 | 182 |
| 141 | [142] | 20 | 38 | 21 | 2.41 | 14.32 | 28 | 11.7 | 30.0 | 14 | 59.4 | 86 |
| 142 | [143] | 21 | 55 | 27 | 2.34 | 16.08 | 16.9 | 12.9 | 5.1 | 4 | 75 | 130 |
| 143 |  | 13 | 65 | 35 | 2.69 | 16.77 | 18.5 | 12.77 | 5.1 | 16 | 71 | 89 |
| 144 | [144] | 65 | 94.51 | 63.96 | 2.68 | 13.34 | 31.5 | 15.3 | 56.7 | 1 | 33.6 | 74.53 |
| 145 | [145] | 36 | 84 | 58 | 2.68 | 13.27 | 29.83 | 16.2 | 8.0 | 22.5 | 41.5 | 189 |

References

1. Taher, Z.J., J. Scalia IV, and C.A. Bareither, *Comparative assessment of expansive soil stabilization by commercially available polymers.* Transportation Geotechnics, 2020. **24**: p. 100387.

2. Al-Rawas, A.A., A. Hago, and H. Al-Sarmi, *Effect of lime, cement and Sarooj (artificial pozzolan) on the swelling potential of an expansive soil from Oman.* Building and Environment, 2005. **40**(5): p. 681-687.

3. Soundara, B. and S. Selvakumar, *Experimental Investigation on the Swelling Behavior of Expansive Soils with EPS Geofoam Inclusion.* Indian Geotechnical Journal, 2020. **50**(4): p. 519-530.

4. Al-Rawas, A.A., *The factors controlling the expansive nature of the soils and rocks of northern Oman.* Engineering Geology, 1999. **53**(3-4): p. 327-350.

5. Shi, B., et al., *Engineering geological characteristics of expansive soils in China.* Engineering Geology, 2002. **67**(1-2): p. 63-71.

6. Phani Kumar, B. and R.S. Sharma, *Effect of fly ash on engineering properties of expansive soils.* Journal of Geotechnical and Geoenvironmental Engineering, 2004. **130**(7): p. 764-767.

7. Sabtan, A.A., *Geotechnical properties of expansive clay shale in Tabuk, Saudi Arabia.* Journal of Asian Earth Sciences, 2005. **25**(5): p. 747-757.

8. Puppala, A.J., K. Punthutaecha, and S.K. Vanapalli, *Soil-water characteristic curves of stabilized expansive soils.* Journal of Geotechnical and Geoenvironmental Engineering, 2006. **132**(6): p. 736-751.

9. Seda, J.H., J.C. Lee, and J.A.H. Carraro, *Beneficial use of waste tire rubber for swelling potential mitigation in expansive soils*, in *Soil improvement*. 2007. p. 1-9.

10. Yan, K. and L. Wu. *Swelling behavior of compacted expansive soils*. in *Recent Advancement in Soil Behavior, in Situ Test Methods, Pile Foundations, and Tunneling: Selected Papers from the 2009 GeoHunan International Conference*. 2009.

11. Zheng, J.-L., R. Zhang, and H.-P. Yang, *Highway subgrade construction in expansive soil areas.* Journal of materials in civil engineering, 2009. **21**(4): p. 154-162.

12. Abdalqadir, Z.K., N.B. Salih, and S.J.H. Salih, *Using Steel Slag for Stabilizing Clayey Soil in Sulaimani City-Iraq.* Journal of Engineering, 2020. **26**(7): p. 145-157.

13. Lin, B. and A. Cerato, *The role of micro-scale properties in the study of expansive soils*, in *Geo-Frontiers 2011: Advances in Geotechnical Engineering*. 2011. p. 4129-4136.

14. Sabat, A.K. and R.P. Nanda, *Effect of marble dust on strength and durability of Rice husk ash stabilised expansive soil.* International Journal of Civil & Structural Engineering, 2011. **1**(4): p. 939-948.

15. Al-Mukhtar, M., S. Khattab, and J.-F. Alcover, *Microstructure and geotechnical properties of lime-treated expansive clayey soil.* Engineering Geology, 2012. **139**: p. 17-27.

16. Gandhi, K.S., *Stabilization of expansive soil of Surat region using rice husk ash and marble dust.* International Journal of Current Engineering and Technology, 2013. **3**(4): p. 1516-1521.

17. Rashid, I., et al., *TREATMENT OF EXPANSIVE CLAYS THROUGH COMPACTION CONTROL.* Pakistan Journal of Science, 2013. **65**(1).

18. Sabat, A.K., *Engineering Properties of an Expansive soil Stabilized with Rice husk ash and Lime sludge.* International Journal of Engineering and Technology, 2013. **5**(6): p. 4826-4833.

19. Malekzadeh, M. and H. Bilsel, *Hydro-mechanical behavior of polypropylene fiber reinforced expansive soils.* KSCE Journal of Civil Engineering, 2014. **18**(7): p. 2028-2033.

20. Radhakrishnan, G., M.A. Kumar, and G. Raju, *Swelling properties of expansive soils treated with chemicals and fly ash.* Am J Eng Res, 2014. **3**(4): p. 245-250.

21. Reddy, N.G., J. Tahasildar, and B.H. Rao, *Evaluating the influence of additives on swelling characteristics of expansive soils.* International Journal of Geosynthetics and Ground Engineering, 2015. **1**(1): p. 7.

22. Zumrawi, M.M., *Geotechnical aspects for roads on expansive soils.* International Journal of Scientific Research, 2015. **4**: p. 896-902.

23. Ameta, N., D. Purohit, and A.S. Wayal, *Characteristics, problems and remedies of expansive soils of Rajasthan, India.* EJGE, 2007. **13**: p. 1-7.

24. Dang, L.C., B. Fatahi, and H. Khabbaz, *Behaviour of expansive soils stabilized with hydrated lime and bagasse fibres.* Procedia engineering, 2016. **143**: p. 658-665.

25. Hasan, H., et al., *Remediation of expansive soils using agricultural waste bagasse ash.* Procedia engineering, 2016. **143**: p. 1368-1375.

26. Shalabi, F.I., I.M. Asi, and H.Y. Qasrawi, *Effect of by-product steel slag on the engineering properties of clay soils.* Journal of King Saud University-Engineering Sciences, 2017. **29**(4): p. 394-399.

27. Zumrawi, M.M. and M.H. Mohammed, *Effect of Fly Ash on the Characteristics of Expansive Soils in Sudan.* University Of Khartoum Engineering Journal, 2017.

28. Zumrawi, M.M., A.M. Mahjoub, and I.M. Alnour, *Effect of Some Chloride Salts on Swelling Properties of Expansive Soil.* University Of Khartoum Engineering Journal, 2017. **6**(2).

29. Akgün, H., et al., *Assessment of the effect of mineralogy on the geotechnical parameters of clayey soils: A case study for the Orta County, Çankırı, Turkey.* Applied Clay Science, 2018. **164**: p. 44-53.

30. Lin, B. and A.B. Cerato, *Applications of SEM and ESEM in microstructural investigation of shale-weathered expansive soils along swelling-shrinkage cycles.* Engineering geology, 2014. **177**: p. 66-74.

31. Dayioglu, M., B. Cetin, and S. Nam, *Stabilization of expansive Belle Fourche shale clay with different chemical additives.* Applied Clay Science, 2017. **146**: p. 56-69.

32. Zumrawi, M.M., *Investigating causes of pavement deterioration in Khartoum state, Sudan.* International Journal of Civil and Environmental Engineering, 2015. **9**(11): p. 1450-1455.

33. Eyo, E.U., S. Ng'ambi, and S. Abbey, *Effect of intrinsic microscopic properties and suction on swell characteristics of compacted expansive clays.* Transportation Geotechnics, 2019. **18**: p. 124-131.

34. Syed, M., A. GuhaRay, and A. Kar, *Stabilization of Expansive Clayey Soil with Alkali Activated Binders.* Geotechnical and Geological Engineering, 2020: p. 1-21.

35. Gupta, C. and R.K. Sharma, *Black cotton soil modification by the application of waste materials.* Periodica polytechnica civil engineering, 2016. **60**(4): p. 479-490.

36. Parik, P. and N.R. Patra. *Static and Dynamic Properties of Expansive Soil Stabilised with Industrial Waste*. in *Geo-Congress 2020: Foundations, Soil Improvement, and Erosion*. 2020. American Society of Civil Engineers Reston, VA.

37. Zumrawi, M.M., A.O. Abdelmarouf, and A.E. Gameil, *Damages of Buildings on Expansive Soils: Diagnosis and Avoidance.* International Journal of Multidisciplinary and Scientific Emerging Research, 2017. **6**(2): p. 108-116.

38. Chittoori, B.C., et al. *Evaluating shallow mixing protocols as application methods for microbial induced calcite precipitation targeting expansive soil treatment*. in *Geo-Congress 2019: Soil Improvement*. 2019. American Society of Civil Engineers Reston, VA.

39. Gheris, A. and A. Hamrouni, *Treatment of an expansive soil using vegetable (DISS) fibre.* Innovative Infrastructure Solutions, 2020. **5**(1): p. 1-17.

40. Phanikumar, B. and R. Singla, *Swell-consolidation characteristics of fibre-reinforced expansive soils.* Soils and Foundations, 2016. **56**(1): p. 138-143.

41. He, S., et al., *Expansive soil treatment with liquid ionic soil stabilizer.* Transportation Research Record, 2018. **2672**(52): p. 185-194.

42. Rosenbalm, D. and C.E. Zapata, *Effect of wetting and drying cycles on the behavior of compacted expansive soils.* Journal of Materials in Civil Engineering, 2017. **29**(1): p. 04016191.

43. Ramesh, P., N. Rao, and K. Murthy, *Efficacy of sodium carbonate and calcium carbonate in stabilizing a black cotton soil.* International Journal of Emerging Technology and Advanced Engineering, 2012. **2**: p. 197-201.

44. Basma, A.A., et al., *Stabilization of expansive clays in Oman.* Environmental & Engineering Geoscience, 1998. **4**(4): p. 503-510.

45. Kumar, T.A., et al., *A Rapid Method of Determination of Swell Potential and Swell Pressure of Expansive Soils Using Constant Rate of Strain Apparatus.* Geotechnical Testing Journal, 2020. **43**(6).

46. Ozer, M., R. Ulusay, and N.S. Isik, *Evaluation of damage to light structures erected on a fill material rich in expansive soil.* Bulletin of Engineering Geology and the Environment, 2012. **71**(1): p. 21-36.

47. Yenes, M., et al., *Shallow foundations on expansive soils: a case study of the El Viso Geotechnical Unit, Salamanca, Spain.* Bulletin of Engineering Geology and the Environment, 2012. **71**(1): p. 51-59.

48. Baby, M., et al., *Experimental study of expansive soil stabilized with TerraZyme.* Int. J. Eng. Res. Technol, 2016. **5**(1): p. 897-899.

49. Mirzababaei, M., A. Arulrajah, and M. Ouston, *Polymers for stabilization of soft clay soils.* Procedia engineering, 2017. **189**: p. 25-32.

50. Kowalska, M. and M. Ptaszek, *Influence of Rubber and Mineral Admixtures on Selected Swelling Properties of Red Clay.* MS&E, 2019. **471**(4): p. 042012.

51. Kaczyński, R. and B. Grabowska-Olszewska, *Soil mechanics of the potentially expansive clays in Poland.* Applied Clay Science, 1997. **11**(5-6): p. 337-355.

52. Azzam, W., *Reduction of the shrinkage–swelling potential with polymer nanocomposite stabilization.* Journal of applied polymer science, 2012. **123**(1): p. 299-306.

53. Carraro, J.A.H., J. Dunham-Friel, and M. Smidt, *Beneficial use of scrap tire rubber in low-volume road and bridge construction with expansive soils*. 2010, Colorado State University. Dept. of Civil and Environmental Engineering.

54. Yazdandoust, F. and S.S. Yasrobi, *Effect of cyclic wetting and drying on swelling behavior of polymer-stabilized expansive clays.* Applied Clay Science, 2010. **50**(4): p. 461-468.

55. Trouzine, H., M. Bekhiti, and A. Asroun, *Effects of scrap tyre rubber fibre on swelling behaviour of two clayey soils in Algeria.* Geosynthetics International, 2012. **19**(2): p. 124-132.

56. Rashid, I., *Characterization and Mapping of Expansive soils of Punjab*. 2015, University of Engineering & Technology, Lahore.

57. Mujtaba, H., et al., *Improvement in engineering properties of expansive soils using ground granulated blast furnace slag.* Journal of the Geological Society of India, 2018. **92**(3): p. 357-362.

58. Pedarla, A., et al., *A Semi-empirical Approach-Based Model for Swell Characterization of Expansive Clays.* Geotechnical and Geological Engineering, 2019. **37**(6): p. 5371-5381.

59. Benyahia, S., et al., *Swelling properties and lime stabilization of N'Gaous expansive marls, NE Algeria.* Journal of African Earth Sciences, 2020: p. 103895.

60. Mumtaz, J., I. Rashid, and J. Israr, *Laboratory modelling of strength and deformation characteristics of a high swelling soil treated with industrial wastes.* Arabian Journal of Geosciences, 2020. **13**(16): p. 1-12.

61. Khennouf, A. and M. Baheddi, *Heave analysis of shallow foundations founded in swelling clayey soil at N’Gaous city in Algeria.* Studia Geotechnica et Mechanica, 2020. **1**(ahead-of-print).

62. She, J., et al., *Experimental study on the engineering properties of expansive soil treated with Al 13.* Scientific Reports, 2020. **10**(1): p. 1-9.

63. Blayi, R.A., et al., *Strength improvement of expansive soil by utilizing waste glass powder.* Case Studies in Construction Materials, 2020. **13**: p. e00427.

64. Sharma, A.K. and P. Sivapullaiah, *Ground granulated blast furnace slag amended fly ash as an expansive soil stabilizer.* Soils and Foundations, 2016. **56**(2): p. 205-212.

65. Kate, J., *Strength and volume change behavior of expansive soils treated with fly ash*, in *Innovations in Grouting and Soil Improvement*. 2005. p. 1-15.

66. Modarres, A. and Y.M. Nosoudy, *Clay stabilization using coal waste and lime—Technical and environmental impacts.* Applied clay science, 2015. **116**: p. 281-288.

67. Puppala, A., et al., *Fiber and fly ash stabilization methods to treat soft expansive soils*, in *Soft ground technology*. 2001. p. 136-145.

68. Bell, F., *Lime stabilization of clay minerals and soils.* Engineering geology, 1996. **42**(4): p. 223-237.

69. Okagbue, C. and T. Onyeobi, *Potential of marble dust to stabilise red tropical soils for road construction.* Engineering Geology, 1999. **53**(3-4): p. 371-380.

70. Saride, S., et al., *Effects of organics on stabilized expansive subgrade soils*, in *Ground Improvement and Geosynthetics*. 2010. p. 155-164.

71. Ene, E. and C. Okagbue, *Some basic geotechnical properties of expansive soil modified using pyroclastic dust.* Engineering Geology, 2009. **107**(1-2): p. 61-65.

72. Oriola, F. and G. Moses, *Groundnut shell ash stabilization of black cotton soil.* Electronic Journal of Geotechnical Engineering, 2010. **15**(1): p. 415-28.

73. Seco, A., et al., *Stabilization of expansive soils for use in construction.* Applied Clay Science, 2011. **51**(3): p. 348-352.

74. Signes, C.H., et al., *Swelling potential reduction of Spanish argillaceous marlstone Facies Tap soil through the addition of crumb rubber particles from scrap tyres.* Applied Clay Science, 2016. **132**: p. 768-773.

75. Thyagaraj, T., et al., *Laboratory studies on stabilization of an expansive soil by lime precipitation technique.* Journal of Materials in Civil Engineering, 2012. **24**(8): p. 1067-1075.

76. Rao, S.M. and T. Thyagaraj, *Lime slurry stabilisation of an expansive soil.* Proceedings of the Institution of Civil Engineers-Geotechnical Engineering, 2003. **156**(3): p. 139-146.

77. Venkara Muthyalu, P., K. Ramu, and G. Prasada Raju, *Study on performance of chemically stabilized expansive soil.* International Journal of Advances in Engineering and Technology, 2012. **2**(1): p. 139-148.

78. Kanawi, M. and A.O. Kamel, *Durability of expansive soil treated by chemical additives.* International Journal of Engineering and Innovative Technology, 2013. **3**(1): p. 315-319.

79. Khemissa, M. and A. Mahamedi, *Cement and lime mixture stabilization of an expansive overconsolidated clay.* Applied Clay Science, 2014. **95**: p. 104-110.

80. Dafalla, M., E. Mutaz, and M. Al-Shamrani. *Compressive strength variations of lime-treated expansive soils*. in *International Foundations Congress and Equipment Expo*. 2015.

81. Sivakumar Babu, G., A. Vasudevan, and M. Sayida, *Use of coir fibers for improving the engineering properties of expansive soils.* Journal of Natural Fibers, 2008. **5**(1): p. 61-75.

82. Amadi, A. and A. Osu, *Effect of curing time on strength development in black cotton soil–Quarry fines composite stabilized with cement kiln dust (CKD).* Journal of King Saud University-Engineering Sciences, 2018. **30**(4): p. 305-312.

83. Osinubi, K., T.S. Ijimdiya, and I. Nmadu. *Lime stabilization of black cotton soil using bagasse ash as admixture*. in *Advanced Materials Research*. 2009. Trans Tech Publ.

84. Dahale, P., P. NAGARNAIK, and A. Gajbhiye, *EFFECT OF FLYASH AND LIME ON STABILIZATION OF EXPANSIVE SOIL.* i-Manager's Journal on Civil Engineering, 2016. **6**(2): p. 8.

85. Eissa, A., et al. *Effect of kerosene contamination on geotechnical properties of clayey soil*. in *International Conference on Advances in Structural and Geotechnical Engineering. ICASGE*. 2017.

86. Acharya, R., et al., *Assessment of guar gum biopolymer treatment toward mitigation of desiccation cracking on slopes built with expansive soils.* Transportation Research Record, 2017. **2657**(1): p. 78-88.

87. Kumar, A., B.S. Walia, and A. Bajaj, *Influence of fly ash, lime, and polyester fibers on compaction and strength properties of expansive soil.* Journal of materials in civil engineering, 2007. **19**(3): p. 242-248.

88. Harichane, K., M. Ghrici, and S. Kenai, *Stabilization of Algerian clayey soils with natural pozzolana and lime.* Periodica Polytechnica Civil Engineering, 2018. **62**(1): p. 1-10.

89. Zumrawi, M.M. and A.A.-A.A. Babikir, *Laboratory Study of Steel Slag Used for Stabilizing Expansive Soil.* University Of Khartoum Engineering Journal, 2017. **6**(2).

90. Ismaiel, H.A. and S.Y. Abdellateef, *Chemical Stabilization of Expansive Esna Shale, Qena Region, Egypt.*

91. Ma, Q.-y., Z.-m. Cao, and P. Yuan, *Experimental research on microstructure and physical-mechanical properties of expansive soil stabilized with fly ash, sand, and basalt fiber.* Advances in Materials Science and Engineering, 2018. **2018**.

92. Sridevi, G., S. Sahoo, and S. Sen, *Stabilization of Expansive Soil with Red Mud and Lime*, in *Ground Improvement Techniques and Geosynthetics*. 2019, Springer. p. 259-268.

93. Mazhar, S., et al. *Stabilization of expansive black cotton soils with alkali activated binders*. in *Proceedings of China-Europe Conference on Geotechnical Engineering*. 2018. Springer.

94. Ali, H. and M. Mohamed, *The effects of lime content and environmental temperature on the mechanical and hydraulic properties of extremely high plastic clays.* Applied Clay Science, 2018. **161**: p. 203-210.

95. Elkady, T.Y., *The effect of curing conditions on the unconfined compression strength of lime-treated expansive soils.* Road Materials and Pavement Design, 2016. **17**(1): p. 52-69.

96. Gonawala, R.J., R. Kumar, and K.A. Chauhan. *Impact of Stabilization of Expansive Clay with Corex Slag and Lime*. in *Geo-Congress 2019: Geotechnical Materials, Modeling, and Testing*. 2019. American Society of Civil Engineers Reston, VA.

97. Pooni, J., et al., *Stabilisation of expansive soils subjected to moisture fluctuations in unsealed road pavements.* International Journal of Pavement Engineering, 2020: p. 1-13.

98. Fattah, M.Y., N.M. Salim, and E.J. Irshayyid, *Swelling behavior of unsaturated expansive soil.* Transportation Infrastructure Geotechnology, 2020: p. 1-22.

99. Ikeagwuani, C.C., et al., *Additives optimization for expansive soil subgrade modification based on Taguchi grey relational analysis.* International Journal of Pavement Research and Technology, 2020: p. 1-15.

100. Jain, A.K. and A.K. Jha, *Geotechnical behaviour and micro-analyses of expansive soil amended with marble dust.* Soils and Foundations, 2020.

101. Ijaz, N., et al., *Integrating lignosulphonate and hydrated lime for the amelioration of expansive soil: A sustainable waste solution.* Journal of Cleaner Production, 2020. **254**: p. 119985.

102. Mazhar, S. and A. GuhaRay, *Stabilization of expansive clay by fibre-reinforced alkali-activated binder: an experimental investigation and prediction modelling.* International Journal of Geotechnical Engineering, 2020: p. 1-17.

103. Sadeeq, J., et al., *Effect of bagasse ash on lime stabilized lateritic soil.* Jordan Journal of Civil Engineering, 2015. **9**(2).

104. Tran, K.Q., T. Satomi, and H. Takahashi. *Study on Effect of Cornsilk Fiber in Cemented Soil Stabilization*. in *Congrès International de Géotechnique–Ouvrages–Structures*. 2017. Springer.

105. Miao, S., et al., *Stabilization of highly expansive black cotton soils by means of geopolymerization.* Journal of Materials in Civil Engineering, 2017. **29**(10): p. 04017170.

106. Kushwaha, S., D. Kishan, and N. Dindorkar, *Stabilization of expansive soil using eko soil enzyme for highway embankment.* Materials today: proceedings, 2018. **5**(9): p. 19667-19679.

107. Öncü, Ş. and H. Bilsel, *Utilization of waste marble to enhance volume change and strength characteristics of sand-stabilized expansive soil.* Environmental earth sciences, 2018. **77**(12): p. 461.

108. Soğancı, A., *The effect of polypropylene fiber in the stabilization of expansive soils.* Int. J. Environ. Chem. Ecol. Geol. Geophys. Eng, 2015. **9**(8): p. 994-997.

109. Hussain, M., *Effect of Lime and Cement on Strength and Volume Change Behavior of Black Cotton Soil*, in *Advances in Computer Methods and Geomechanics*. 2020, Springer. p. 651-663.

110. Al-Khashab, M.N. and A.-H.M. Thafer, *Treatment of Expansive Clayey Soil with Crushed Limestone.* Civil Eng. Dept. Collage of Eng. Mosul University. Engineering & Technology Journal, 2008. **26**: p. 376-386.

111. Solanki, P., N. Khoury, and M.M. Zaman, *A comparative evaluation of various additives used in the stabilization of sulfate bearing lean clay.* Journal of ASTM International, 2009. **6**(8): p. 1-18.

112. Patel, H., *Laboratory assessment to correlate strength parameter from physical properties of subgrade.* Procedia Engineering, 2013. **51**: p. 200-209.

113. Kolay, P.K. and M.A. Rahman, *Physico-geotechnical properties of peat and its stabilisation.* Proceedings of the Institution of Civil Engineers-Ground Improvement, 2016. **169**(3): p. 206-216.

114. Khalid, U., et al., *Prediction of unconfined compressive strength from index properties of soils.* Sci Int (Lahore), 2015. **27**(5): p. 4071-4075.

115. Alazigha, D.P., et al., *The swelling behaviour of lignosulfonate-treated expansive soil.* Proceedings of the Institution of Civil Engineers-Ground Improvement, 2016. **169**(3): p. 182-193.

116. Canakci, H., A. Aram, and F. Celik, *Stabilization of clay with waste soda lime glass powder.* Procedia engineering, 2016. **161**: p. 600-605.

117. Onyelowe, K., et al., *Comparison between the strength characteristics of pozzolan stabilized lateritic soil of coconut shell husk ash and palm kernel shell husk ash admixtures.* Am Res J Civil Struct Eng, 2016. **1**: p. 1-8.

118. Onyelowe, K., *Effect of temperature changes on the unconfined compressive strength of OPC stabilized engineering soil with palm bunch ash, PBA as admixture.* IISTE J Civil Environ Res, 2016. **8**: p. 20-7.

119. Gadouri, H., K. Harichane, and M. Ghrici, *Effect of calcium sulphate on the geotechnical properties of stabilized clayey soils.* Periodica Polytechnica Civil Engineering, 2017. **61**(2): p. 256-271.

120. Onyelowe, K., *Nanostructured waste paper ash stabilization of lateritic soils for pavement base construction purposes.* Electronic Journal of Geotechnical Engineering, 2017. **22**(09): p. 3633-3647.

121. Shafiqu, Q.S.M. and S.H. Hasan. *Improvement an Expansive Soil using Polymethacrylate Polymer*. in *IOP Conference Series: Materials Science and Engineering*. 2018. IOP Publishing.

122. Tabet, W.E., et al., *Characterization of Hydration Products’ Formation and Strength Development in Cement-Stabilized Kaolinite Using TG and XRD.* Journal of Materials in Civil Engineering, 2018. **30**(10): p. 04018261.

123. Abd El-Aziz, M.A. and M.A. Abo-Hashema, *Measured effects on engineering properties of clayey subgrade using lime–Homra stabiliser.* International Journal of Pavement Engineering, 2013. **14**(4): p. 321-332.

124. Akinwumi, I.I., et al., *Investigation of Calcium Carbide Residue as a Stabilizer for Tropical Sand used as Pavement Material.* WIT Transactions on The Built Environment, 2019. **187**: p. 285-294.

125. Almurshedi, A.D., J.K. Thajeel, and H.C. Dekhn. *Swelling Control of Expansive Soils Using Cement Dust*. in *IOP Conference Series: Materials Science and Engineering*. 2019. IOP Publishing.

126. Emmanuel, E., et al., *Stabilization of a soft marine clay using halloysite nanotubes: A multi-scale approach.* Applied Clay Science, 2019. **173**: p. 65-78.

127. Irshayyid, E.J. and M.Y. Fattah. *The Performance of Shear Strength and Volume Changes of Expansive Soils Utilizing Different Additives*. in *IOP Conference Series: Materials Science and Engineering*. 2019. IOP Publishing.

128. Pourakbar, S., et al., *Model study of alkali-activated waste binder for soil stabilization.* International Journal of Geosynthetics and Ground Engineering, 2016. **2**(4): p. 35.

129. Murmu, A.L., A. Jain, and A. Patel, *Mechanical properties of alkali activated fly ash geopolymer stabilized expansive clay.* KSCE Journal of Civil Engineering, 2019. **23**(9): p. 3875-3888.

130. Saputra, N.A. and R. Putra. *The Correlation Between CBR (California Bearing Ratio) and UCS (Unconfined Compression Strength) Laterite Soils in Palangka Raya as Heap Material*. in *IOP Conference Series: Earth and Environmental Science*. 2020. IOP Publishing.

131. Khalid, U., et al., *A simple experimental method to regain the mechanical behavior of naturally structured marine clays.* Applied Ocean Research, 2019. **88**: p. 275-287.

132. Adeyanju, E., et al., *Subgrade Stabilization using Rice Husk Ash-based Geopolymer (GRHA) and Cement Kiln Dust (CKD).* Case Studies in Construction Materials, 2020: p. e00388.

133. Tiwari, N., N. Satyam, and S.K. Shukla, *An experimental study on micro-structural and geotechnical characteristics of expansive clay mixed with EPS granules.* Soils and Foundations, 2020.

134. Baldovino, J.d.J.A., et al., *Geopolymers Based on Recycled Glass Powder for Soil Stabilization.* Geotechnical and Geological Engineering, 2020. **38**(4): p. 4013-4031.

135. Mir, B. and K.M.N.S. Wani, *Mechanical Behavior of Boulder Crusher Dust (BCD)-Stabilized Dredged Soil*, in *Problematic Soils and Geoenvironmental Concerns*. 2018, Springer. p. 421-432.

136. Eyo, E.U., et al., *Incorporation of a nanotechnology-based product in cementitious binders for sustainable mitigation of sulphate-induced heaving of stabilised soils.* Engineering Science and Technology, an International Journal, 2020.

137. Ali, H.E., et al., *Effect of sodium compounds additives on the strength of cement-stabilized soils.* Engineering and Applied Science Research, 2020. **47**(3): p. 287-296.

138. Indiramma, P., C. Sudharani, and S. Needhidasan, *Utilization of fly ash and lime to stabilize the expansive soil and to sustain pollution free environment–An experimental study.* Materials Today: Proceedings, 2020. **22**: p. 694-700.

139. Khodabandeh, M.A., et al., *The Effect of Acidic and Alkaline Chemical Solutions on the Behavior of Collapsible Soils.* Periodica Polytechnica Civil Engineering, 2020. **64**(3): p. 939-950.

140. Krishnan, K.D. and P. Ravichandran. *Investigation on Industrial Waste Material for Stabilizing the Expansive Soil*. in *IOP Conference Series: Materials Science and Engineering*. 2020. IOP Publishing.

141. Lu, Y., et al., *Freeze-thaw performance of a cement-treated expansive soil.* Cold Regions Science and Technology, 2020. **170**: p. 102926.

142. Meeravali, K., et al., *An analysis of freeze-thaw cycles on geotechnical properties of soft-soil.* Materials Today: Proceedings, 2020.

143. Nikhil, P.S., P. Ravichandran, and K.D. Krishnan, *Stabilisation and characterisation of soil using wollastonite powder.* Materials Today: Proceedings, 2020.

144. Niyomukiza, J. and S.R.a.B. Setiadji, *The influence of Keruing sawdust on the geotechnical properties of expansive Soils.* E&ES, 2020. **448**(1): p. 012040.

145. Phanikumar, B. and E.R. Raju, *Compaction and strength characteristics of an expansive clay stabilised with lime sludge and cement.* Soils and Foundations, 2020.
